# Mechanochemically Triggered Topology Changes in Expanded Porphyrins

**DOI:** 10.1002/chem.202003869

**Published:** 2021-01-18

**Authors:** Tom Bettens, Marvin Hoffmann, Mercedes Alonso, Paul Geerlings, Andreas Dreuw, Frank De Proft

**Affiliations:** ^1^ Eenheid Algemene Chemie (ALGC) Vrije Universiteit Brussel (VUB) Pleinlaan 2 1050 Brussels Belgium; ^2^ Interdisciplinary Center for Scientific Computing Ruprecht-Karls University Im Neuenheimer Feld 205A 69120 Heidelberg Germany

**Keywords:** expanded porphyrins, JEDI analysis, mechanochemistry, molecular switches, topology

## Abstract

A hitherto unexplored class of molecules for molecular force probe applications are expanded porphyrins. This work proves that mechanical force is an effective stimulus to trigger the interconversion between Hückel and Möbius topologies in [28]hexaphyrin, making these expanded porphyrins suitable to act as conformational mechanophores operating at mild (sub‐1 nn) force conditions. A straightforward approach based on distance matrices is proposed for the selection of pulling scenarios that promote either the planar Hückel topology or the three lowest lying Möbius topologies. This approach is supported by quantum mechanochemical calculations. Force distribution analyses reveal that [28]hexaphyrin selectively allocates the external mechanical energy to molecular regions that trigger Hückel–Möbius interconversions, explaining why certain pulling scenarios favor the Hückel *two‐sided* topology and others favor Möbius *single‐sided* topologies. The *meso*‐substitution pattern on [28]hexaphyrin determines whether the energy difference between the different topologies can be overcome by mechanical activation.

## Graphical abstract

**Use the force**: The Hückel and Möbius topology of [28]hexaphyrin can be mechanically locked by applying an external pulling force to different *meso* positions in a *sub*‐nano‐Newton force regime. The mechanical energy is distributed to a specific region in the molecule to trigger the interconversion.

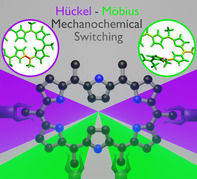

## 1 Introduction

Mechanochemistry has attracted increased attention in the past decade with the development of techniques to distort single molecules.^[1]^ In parallel to the experimental progress in this field—sometimes called the fourth subclass in chemistry besides thermochemistry, photochemistry, and electrochemistry—theoretical investigations are crucial to understand the often unique reaction channels promoted by mechanical activation, resulting in several combined experimental/theoretical investigations.[^2^, ^3^, ^4^] Computational methods become particularly relevant when scaling down the system size to individual molecules or even *mechanophores*, that is, molecules or part of molecules that respond to mechanical activation. Several quantum mechanochemical methods have been developed to model external forces quantum chemically,[^2a^, ^5^] but the role of theoretical methods is not limited to computing reaction pathways under force.

The directional character of an external mechanical force drastically impacts the molecular geometry, and the distribution of mechanical energy within a molecule can be quantified by means of the JEDI (judgement of energy distribution) analysis,^[6]^ revealing to what extent different pulling scenarios deform different regions in a molecule.^[7]^ Recently, the concept of ring strain was also re‐established from a mechanical viewpoint by using this strain analysis tool.^[8]^ The alteration of bond lengths and angles by mechanical force was shown to be an efficient way to modify chemical reactivity properties in an extended conceptual DFT framework.^[9]^ In particular, redox properties as well as the nucleophilic/electrophilic character of molecular regions can be fine‐tuned by mechanical strain.^[10]^

Molecular force probes are a special type of mechanophores that allow for the detection and quantification of local mechanical stress in a single molecule or material owing to a measurable change in physical properties—mostly spectroscopic properties of *mechanochromic* compounds.^[11]^ Such exciting molecular force probes can be designed by using theoretical approaches, closing the gap between quantum chemical calculations and real‐life applications.[^11e^, ^12^] These species rely on the transfer of mechanical energy to trigger a conformational change, leading to different measurable properties. The most intensively investigated force probe is based on the mechanically triggered spiropyran‐merocyanine isomerization in polymers, which relies on the rupture of a labile spiro C−O bond in the mechanochromic spiropyran.[^13^, ^14^] This bond is not directly broken but strained (elongated), triggering a 6π ring‐opening to the merocyanine form. However, the activation of one bond usually competes with the activation of other bonds, and the rupture of other bonds is undesirable as it is likely to cause degradation of the mechanophore, avoiding which is one of the major challenges in the design of molecular force probes.

Conformational mechanophores, on the contrary, do not rely on the scission of a (labile) bond and have attracted more attention in recent years.^[15]^ Conformational changes, which are essentially triggered by rotations around bonds, typically require less energy than bond length elongations and can be realized at lower force conditions. Indeed, molecules are known to absorb force by first distorting soft modes before significant bond length elongation occurs.^[16]^ Also, a mechanophore is more likely to recover from a dihedral angle inversion than from an undesirably broken bond. Therefore, conformational mechanophores offer two windows of opportunity to improve molecular force sensors.

**Figure 1:**
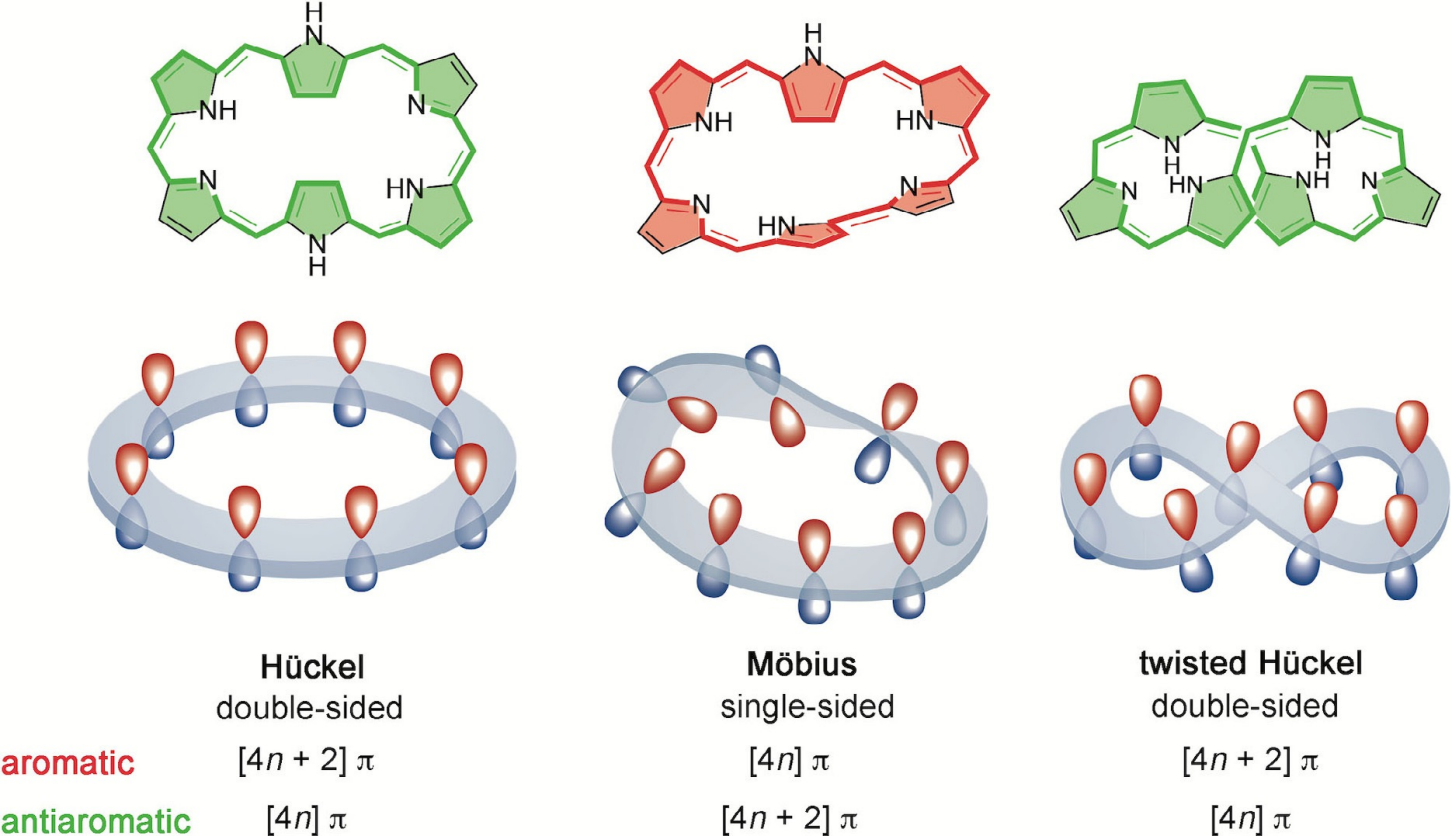
Schematic representation of different π‐conjugation topologies of [28]hexaphyrin and their expected aromaticity as a function of the number of π‐electrons.

A hitherto unexplored class of molecules in this context is represented by expanded porphyrins, the larger analogs of porphyrin, consisting of more than four pyrrole rings or alternative heterocyclic subunits connected either directly or by bridging atoms.^[17]^ These extended π‐macrocycles are promising building blocks for multiple applications taking advantage of their large conformational flexibility, multiple oxidation states, and versatile aromaticity.^[18]^ One of the most appealing features of expanded porphyrins is their ability to switch between different π‐conjugation topologies, each with distinct optoelectronic properties and aromaticity.^[19]^ The different topologies are labeled as Hückel or Möbius owing to the fulfilment of the corresponding aromaticity rules (Figure 1).^[20]^ The change of topology is achieved by variation of one internal dihedral angle and, if properly controlled, can provide access to molecular switches with unique optical, transport, and magnetic properties.^[21]^ Indeed, upon topology and redox interconversions, expanded porphyrins have recently been demonstrated to act as efficient multi‐level molecular switches for challenging nanoelectronic applications, including conductance switching,^[22]^ bithermoelectric devices,^[23]^ and nonlinear optical switches.^[24]^ Novel structure–property relationships in the field of molecular optoelectronics have been derived^[25]^ and show how the concept of aromaticity and molecular topology can be exploited to fine‐tune the quantum interference effects in charge transport through single‐molecule junctions.^[26]^

This novel type of molecular switches can be triggered by different external stimuli, such as light,^[27]^ metalation,^[28]^ solvent,^[29]^ and pH.^[30]^ An external force might be an alternative way to activate Hückel–Möbius switches, but no studies have been reported so far for the mechanical activation of expanded porphyrins. Nevertheless, recent research has demonstrated the potential of mechanical forces to induce tautomerization of porphycene, a structural isomer of porphyrin.^[31]^ By a combination of scanning probe microscopy and DFT calculations, it has been shown that mechanical activation constitutes a new way to operate a single‐molecular switch.

This work explores for the first time the role of external forces on topology interconversions and investigates how mechanical force influences the conformation of expanded porphyrins. In particular, we focus on [28]hexaphyrin, for which aromatic Möbius and antiaromatic Hückel structures coexist in dynamic equilibrium, as proven by experimental^[32]^ and theoretical^[33]^ studies. Among porphyrinoids, hexaphyrins consisting of six pyrrolic units have shown exceptional properties for molecular switching devices,[^21^, ^27^, ^28^, ^34^] exhibiting very large conductance ratios and NLO contrasts upon redox and topology interconversions.[^22^, ^35^] In this work, we put forward a simple yet very effective approach based on distance matrices to select optimal pulling conditions for mechanically locking a desired conformer of [28]hexaphyrin. By means of ab initio calculations and force distribution analysis, we show expanded porphyrins to selectively absorb mechanical energy triggering Hückel–Möbius interconversions.

## 2 Computational Methods

### 2.1 EFEI approach and JEDI analysis

External forces were modeled by using the EFEI (external forces explicitly included) ansatz.[^5a^, ^5b^] In this approach, the force is explicitly included in the gradient during the geometry optimization, in contrast to non‐explicit methods such as the COGEF (constrained geometries simulate external forces) approach,^[5d]^ which is sometimes used for constraining interatomic distances and, recently, extended for also constraining bond angles.^[10a]^ Using a constant external force $F_{ext}$ , the external mechanical energy is directly proportional to the distance between the pulling positions.

(1) $$V_{EFEI}\left(x,F_{ext}\right)=\;V_{BO}\left(x\right)-\;F_{ext}\;r\left(x\right)$$

In this equation, r is the distance between the pulling points (atoms) and $V_{BO}$ is the energy on the Born–Oppenheimer (BO) potential energy surface for a set of cartesian coordinates x , which is obtained by performing a geometry optimization under force. Thus, also r should be computed when the external force is active (see below). The vector corresponding to $F_{ext}$ is aligned with the vector connecting the two pulling points, justifying the simple product form of the force‐dependent term.

Applying Equation (1) to a Hückel–Möbius (**H**‐**M**) equilibrium of [28]hexaphyrin gives in the absence of an external force

(2) $$\Delta V\left(F_{ext}=0\right)=\;V_{BO}\left(M,F_{ext}=0\right)-\;V_{BO}\left(H,F_{ext}=0\right)$$

and with an external force

(3) $$\Delta V\left(F_{ext}\neq 0\right)=\;V_{BO}\left(M,F_{ext}\neq 0\right)-\;V_{BO}\left(H,F_{ext}\neq 0\right)-\;F_{ext}\;\left[r\left(M,F_{ext}\neq 0\right)-r\left(H,F_{ext}\neq 0\right)\right]$$

Then, the shift of the **H**‐**M** equilibrium owing to the external force, ΔΔV , can be written as a sum of a term that depends on the energy of the Hückel and Möbius structures on the BO potential energy surface and a term that depends on the constant external force and the distance between the pulling points in the Hückel and Möbius structures:

(4) $$\Delta \Delta V=\;\Delta \Delta V_{BO}-F_{ext}\;\Delta r$$

with

(5) $$\Delta \Delta V_{BO}=\;V_{BO}\left(M,F_{ext}\neq 0\right)-\;V_{BO}\left(H,F_{ext}\neq 0\right)-\left[V_{BO}\left(M,F_{ext}=0\right)-\;V_{BO}\left(H,F_{ext}=0\right)\right]$$

(6) $$F_{ext}\;\Delta r\;=\;F_{ext}\;\left[r\left(M,F_{ext}\neq 0\right)-r\left(H,F_{ext}\neq 0\right)\right]$$

We computed the distribution of the mechanical energy in [28]hexaphyrin owing to $F_{ext}$ , which is typically not uniform, by means of the JEDI (judgement of energy distribution) analysis,^[6]^ expressed by Equation 7. This tool is based on the harmonic approximation and quantifies the change in energy Δ*E_i_* owing to the deformation of each redundant internal coordinate i (bond length, bond angle, and dihedral angle) in a molecule.

(7) $${\Delta E}_{i}=\;\frac{1}{2}\sum_{j}^{M}{\left.\frac{\partial^{2}V\left(q\right)}{\partial q_{i}\partial q_{j}}\right|}_{q=q_{0}}\Delta q_{i}\Delta q_{j}$$

Here, $\frac{\partial^{2}V\left(q\right)}{\partial q_{i}\partial q_{j}}$ is a Hessian matrix element at the equilibrium geometry in redundant internal coordinates and Δ*q_i_* is the change in redundant internal coordinate i upon mechanical deformation. Importantly, the harmonic approximation works best close to the equilibrium where the perturbation is small and, thus, for small external forces. The approximation becomes particularly troublesome when dihedral angles flip, resulting in unphysically large contributions to Δ*E*_JEDI_, which is the sum of all Δ*E_i_* terms.^[6]^ A total of three calculations are required to carry out JEDI analyses: a geometry optimization and Hessian calculation of the unperturbed system and a geometry optimization of the (mechanically) distorted system. The interested reader is referred to reference [7e] for an in‐depth treatment of the mathematical foundation of the JEDI approach. An in‐house code interfaced with the Q‐chem program was used to perform the required matrix transformations.

### 2.2 Computational details

All calculations were carried out at the M06‐2X/6–311G(d,p)//M06‐2X/6‐31G level of theory by using the Q‐Chem (version 5.1) software.[^36^, ^37^] In a recent benchmark study against canonical CCSD(T)/CBS reference energies, it was demonstrated that an accurate description of the relative energies of Hückel–Möbius interconversions in expanded porphyrins is difficult for most of the density functionals, wavefunction methods, and even localized orbital coupled cluster methods.^[38]^ Among fifty exchange‐correlation functionals tested, the *meta*‐GGA functional M06‐2X was shown to provide accurate relative energies for topology interconversions in hexaphyrins, in addition to computationally more demanding range‐separated double hybrids.^[39]^ These benchmarking studies also report a small basis set dependence on relative Hückel–Möbius energies of [28]hexaphyrin. In particular, the expansion from the 6‐31G basis to 6–311G(d,p) was found to have a small influence on relative energies.^[33b]^ The basis set dependence was investigated for a set of Hückel–Möbius interconversions (see below). The nature of stationary points was verified through vibrational analysis to ensure that optimized geometries correspond to minima on the potential energy surface. Vibrational frequencies, which are required to compute the change in energy owing to redundant internal coordinates according to Equation (7), were computed numerically. On pulling positions, CH_3_ substituents were used to represent a polymer backbone or lever arm in experimental setups. External forces, which were applied to one of the H atoms of the CH_3_ substituents, were included by using the EFEI approach, as implemented in Q‐Chem.

## 3 Results and Discussion

### 3.1 Selection of appropriate pulling positions

**Figure 2:**
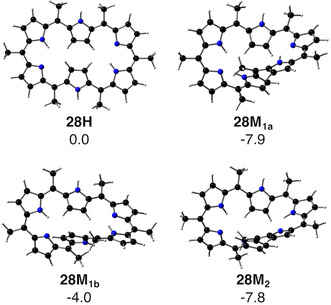
The Hückel (**28 H)** and Möbius structures (**28 M_1a_**, **28 M_1b_**, and **28 M_2_**) of [28]hexaphyrin (black=C, blue=N, white=H). Relative electronic energies are in kcal mol^−1^.

**Figure 3:**
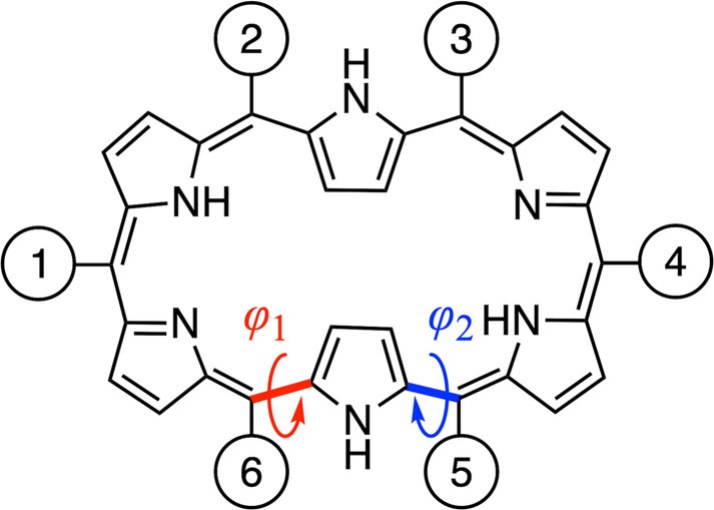
Numbering of the *meso* positions of the **28 H** hexaphyrin macrocycle in this work. This Hückel structure interconverts to **28 M_1a_** or **28 M_1b_** by rotating the dihedral angle *ϕ*_1_ or *ϕ*_2_, respectively.

To select appropriate pulling conditions for mechanically forcing the conformation of [28]hexaphyrin, we first analyze the structures and relative energies of the most stable conformers of the relaxed macrocycle, that is, in the absence of external force. Previous studies have shown that [28]hexaphyrin undergoes fast conformational dynamics between several twisted Möbius conformers and planar Hückel structures, with the Möbius topology being thermodynamically most stable.[^32b^, ^33a^] The exhaustive study of the reaction mechanism performed by Torrent‐Sucarrat and co‐workers^[33b]^ showed the existence of two competing pathways, labeled **a** and **b**, for the interconversion between the planar Hückel (**28 H)** and the Möbius structure (**28 M_2_** in Figure 2). The two pathways differ in the rotating carbon–carbon bond leading to the intermediate Möbius structures (**28 M_1a_** and **28 M_1b_**). Mechanism A involves the inversion of the *ϕ*_1_ dihedral angle (i.e., rotation around the red bond in Figure 3), whereas mechanism B involves the inversion of the *ϕ*_2_ angle (i.e., rotation around the blue bond in Figure 3). The second step in both mechanisms corresponds to proton transfer between two nitrogen atoms, leading to the final tautomer of the Möbius structure (**28 M_2_**). In these mechanisms, the rate‐determining step can be either the bond rotation or the proton transfer, depending on the rotating bond and the *meso*‐substituents.^[33b]^ The low activation energy barriers and the small energy difference between the Hückel and Möbius conformers reported in the literature corroborate that both conformers are easily and rapidly interconverting at room temperature (see, for example, Figure 6 in Ref [39]).

Figure 2 shows the 3D structures of the lowest‐energy conformers of [28]hexaphyrin with methyl‐substituents on all *meso* positions, namely a Hückel topology (**28 H)** as well as three Möbius topologies (**28 M_1a_**, **28 M_1b_**, and **28 M_2_**). According to Figure 2, the Möbius topologies are lower in energy than the Hückel topology with **28 M_1a_** and **28 M_2_** being the most stable conformers, in good agreement with experimental observations for *meso*‐aryl [28]hexaphyrins.^[32]^ Importantly, in the case of all *meso*‐CH_3_ substituents, the lowest electronic energy was found for the twisted figure‐of‐eight topology (see Figure S1 in the Supporting Information) with an energy of −11.0 kcal mol^−1^, relative to the **28 H** conformer, similar to *meso*‐CF_3_‐[28]hexaphyrin.^[40]^ However, the stability of this conformer is specific for the *meso*‐CX_3_ substitution pattern and strongly dependent on temperature and solvent.^[41]^ Therefore, only the planar Hückel and Möbius topologies were considered herein.

To invert the conformational equilibrium, a pulling position should be identified that favors **28 H** over **28 M_1a_**, **28 M_1b_**, and **28 M_2_**. Peripheral functionalization in hexaphyrins has been achieved by substitution at the β‐ and/or *meso* positions of the macrocycle with various substituents and the conformations of hexaphyrins were shown to be heavily dependent on these peripheral substituents.^[17]^ As *meso* substitution retains the central macrocyclic framework, we decided to consider the six *meso* positions as pulling positions (see Figure 3). In particular, we use methyl groups at each *meso* position rather than hydrogen atoms, as the synthesized hexaphyrins commonly bear substituents at *meso* positions, such as pentafluorophenyl groups, 2,6‐disubstituted aryl groups, or trifluoromethyl substituents.[^32b^, ^40^, ^41^] The use of a methyl substituent rather than a H atom also makes the structures more realistic as a side‐chain is required to perform any laboratory experiment.

In mechanochemistry, the efficiency of mechanical activation strongly depends on the pulling positions and the applied force. We identified appropriate pulling positions by using a very straightforward approach: *the expression in Equation (4) indicates that the shift of the **H**‐**M** equilibrium will be larger if the distance between the pulling positions is significantly larger in one structure than in the other for* $F_{ext}\neq 0$ . Therefore, the distance matrices of the Hückel and Möbius structures contain valuable information for inverting the Hückel–Möbius equilibrium by mechanical force.

Table 1 shows the fraction of the total distance matrices containing the *meso* C atoms of **28 H**, **28 M_1a_**, **28 M_1b_** and **28 M_2_** and Figure 3 illustrates the numbering of the *meso* positions. The **28 H** structure exhibits *C_i_* symmetry; hence, the centrosymmetric distances are identical. Conveniently, a total of six *meso* carbon–carbon distances are larger in the Hückel than all of the Möbius conformers (see Table 1), namely C^1^−C^4^, C^1^−C^5^, C^2^−C^5^, C^2^−C^6^, C^3^−C^5^, and C^3^−C^6^. Because the Möbius conformers have no inversion center, C^2^−C^6^ and C^3^−C^5^ are different. Thus, based on this distance matrix approach, each of these pulling scenarios is hypothesized to favor the Hückel conformer over the Möbius conformer. Moreover, one distance (C^5^−C^6^) is always larger in the Möbius structures. Following the same reasoning, pulling at these positions is expected to cause the opposite effect, namely locking the Möbius topologies in even deeper minima on the potential energy surface with respect to the Hückel topology.

**Table 1:** The fraction of the distance matrix of **28 H**, **28 M_1a_**, **28 M_1b_**, and **28 M_2_** containing the *meso* C atoms, in Å.

		C^1^	C^2^	C^3^	C^4^	C^5^	C^6^
**28 H**	C^1^	0					
	C^2^	7.146	0				
	C^3^	10.956	4.751	0			
	C^4^	14.821	10.953	7.021	0		
	C^5^	10.953	11.064	9.905	7.146	0	
	C^6^	7.021	9.905	10.908	10.956	4.751	0
							
		C^1^	C^2^	C^3^	C^4^	C^5^	C^6^
**28 M_1a_**	C^1^	0					
	C^2^	7.093	0				
	C^3^	11.159	5.163	0			
	C^4^	13.735	10.465	7.084	0		
	C^5^	7.179	7.441	8.267	7.229	0	
	C^6^	7.132	8.907	9.577	10.570	6.731	0
							
		C^1^	C^2^	C^3^	C^4^	C^5^	C^6^
**28 M_1b_**	C^1^	0					
	C^2^	7.186	0				
	C^3^	10.301	5.118	0			
	C^4^	13.823	11.041	7.062	0		
	C^5^	10.658	9.568	9.142	7.205	0	
	C^6^	7.193	8.740	8.004	7.672	6.439	0
							
		C^1^	C^2^	C^3^	C^4^	C^5^	C^6^
**28 M_2_**	C^1^	0					
	C^2^	7.170	0				
	C^3^	10.126	5.154	0			
	C^4^	13.633	11.097	7.162	0		
	C^5^	10.660	9.397	9.055	7.152	0	
	C^6^	7.198	8.674	7.727	7.296	6.525	0

Importantly, these distance matrices were computed without any external force acting on the molecule, whereas the distance $r\left(x\right)$ in the EFEI‐term in Equation (1) is under force, as detailed above. Therefore, once the force is applied, the correct conformer will only be favored if the attachment point distances remain larger under force in one conformer than the other under force. We thus use the distances in Table 1 only as guidelines to identify appropriate pulling positions. In other words, our approach based on distance matrices of relaxed structures will only be valid if the pattern in the *meso* distances is conserved, independent of the pulling scenario. In addition, we assume that $\Delta \Delta V_{BO}$ in Equation (4)—as a result of moving away from the equilibrium on the potential energy surface of the relaxed molecule owing to the external force—has only a negligible influence on the relative Hückel–Möbius energies. Below, we investigate the validity of using distance matrices to select appropriate pulling positions in detail.

### 3.2 Forcing the topology of [28]hexaphyrin

From the distance matrices of the Hückel and Möbius conformers, six pulling scenarios can be identified probably favoring the Hückel structure and one pulling scenario was recognized to favor the Möbius structures. Table 2 summarizes the carbon–carbon *meso* distances in the Hückel and Möbius structures along the selected pulling coordinates above. It is important to remark that **28 H** loses the *C_i_* symmetry in the 1–5 and 5–6 pulling scenarios when a force of 0.333 nn is applied. At 1.0 nn, all Hückel structures have *C*_1_ symmetry. The cartesian coordinates of each structure are provided in the Supporting Information. As anticipated, the *meso* distances in **28 H** remain larger than in **28 M_1a_**, **28 M_1b_** and **28 M_2_** for the 1–4, 1–5, 2–5, 2–6, 3–5, and 3–6 pulling scenarios at 0.333 nn and 1.0 nn. However, there is one exception: the C^1^−C^5^ distance becomes slightly larger in the **28 M_1b_** and **28 M_2_** structures than in **28 H** for $F_{ext}$ =1.0 nn. The *meso* distance is smaller in the Hückel structure than in the Möbius structures for the 5–6 pulling scenario. Therefore, the selection of appropriate pulling positions in the unperturbed structures is viable for [28]hexaphyrin. It is also worth noticing that the *meso* distances in the strained structures in Table 2 are larger than for the unperturbed structures in Table 1, as an external pulling force will generally increase the distance between the pulling points.

**Table 2:** *Meso* carbon–carbon distances (in Å) in all [28]hexaphyrin conformers when an external force is applied. The external force was applied to one of the H atoms of the methyl substituents on these *meso* positions.

*Meso* distance	**28 H**		**28 M_1a_**		**28 M_1b_**		**28 M_2_**	
	0.333 nn	1.0 nn	0.333 nn	1.0 nn	0.333 nn	1.0 nn	0.333 nn	1.0 nn
C^1^−C^4^	14.907	16.369	13.924	14.316	14.015	14.487	13.779	14.191
C^1^−C^5^	11.067	11.285	8.328	10.603	10.912	11.429^[a]^	10.890	11.326^[a]^
C^2^−C^5^	11.276	11.660	9.166	–	10.388	11.473	10.364	11.465
C^2^−C^6^	9.978	10.302	9.201	–	8.948	9.426	8.983	9.338
C^3^−C^5^	9.997	10.302	8.733	8.878	9.431	9.917	9.349	9.838
C^3^−C^6^	11.144	11.572	10.369	–	9.356	–	9.023	–
C^5^−C^6^	5.217	–	6.810	6.992	6.629	6.864	6.669	6.967

[a] The *meso* distance is larger in the Möbius topologies.

In the stronger force regime (1.0 nn), geometry convergence issues occurred because of the low activation energy barriers for the Hückel–Möbius topology interconversions and other low rotational barriers along dihedral angles. This is an indication that the critical force, at which an energy minimum and transition state coalesce, is exceeded.^[42]^ The flip of dihedral angles are more prone to such changes of the force‐modified potential energy surface than, for example, rupture of covalent bonds, which typically occurs at higher force.[^1c^, ^43^] In particular, the **28 M_1a_** conformer seems very sensitive to external forces as it only converged to the desired Möbius topology when pulling at the 1–4, 3–5, or 5–6 positions. Interestingly, for 2–5, 2–6, and 3–6 pulling, the **28 M_1a_** geometry converged to a structure in which an undesired dihedral angle was inverted (see Figure S2 in the Supporting Information). The **28 M_1b_** and **28 M_2_** structures did not converge to the correct Möbius topology for the 3–6 pulling scenario, whereas **28 H** did not converge to a Hückel structure for the 5–6 pulling scenario at 1.0 nn. As several conformers no longer converge to the desired structures, a force of 1.0 nn can be considered as the maximum force regime for these interconversions associated with soft modes.

**Figure 4:**
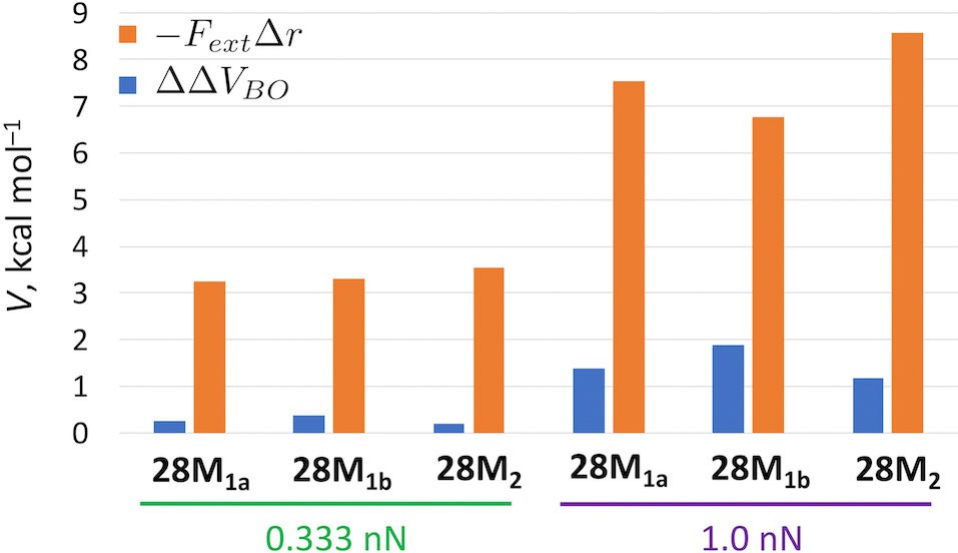
Contribution of the $\Delta \Delta V_{BO}$ (blue) and the $F_{ext}\;\Delta r$ term (orange) in Equation (4) to the shift in relative Hückel–Möbius energies for the 1–4 pulling scenario.

We quantified the contribution of the $\Delta \Delta V_{BO}$ and $F_{ext}\;\Delta r$ terms in Equation (4) for each of the pulling scenarios. Figure 4 illustrates these contributions for the 1–4 pulling scenario under 0.333 nn and 1.0 nn conditions. As anticipated, the force‐term (in orange) dominates the total shift in relative Hückel–Möbius energies. Importantly, the force‐dependent term is directly proportional to the pulling point distances in the Hückel and Möbius topologies of [28]hexaphyrin and, therefore, a larger difference in *meso* distances is desired. This is the case for the *meso* 1–4 distances, which are about 1 Å larger in the Hückel topology than the Möbius topology in Tables 1 and 2. In Figure S3 and Figure S4 in the Supporting Information, the same analysis is shown for all other pulling scenarios. The difference in *meso* distances for, for example, **28 M_1b_** and **28 M_2_** with respect to the Hückel structure is less significant in the case of 2–5 and 3–5 pulling with an external force of 1.0 nn, which is reflected in the smaller influence of the force‐dependent term in Figure S3 c and S3 e (in the Supporting Information).

Table 3 summarizes the energies of all Möbius conformers (relative to the Hückel conformer) when a force of 0.333 nn and 1.0 nn is used (see Table S1 in the Supporting Information for relative enthalpies and Gibbs free energies at 298.15 K). Here, a positive sign indicates a more stable Hückel structure, whereas a negative sign means that the Möbius topology is more stable than the Hückel one. The data under zero‐force conditions are also listed as reference.

**Table 3:** Energies (in kcal mol^−1^) of the Möbius conformers, relative to the Hückel topology **28 H** for different force regimes computed at the M06‐2X/6–311G(d,p)//M06‐2X/6‐31G level of theory. A positive sign indicates a more stable Hückel structure, whereas a negative sign means the opposite.

Force	Pulling scenario	**28 M_1a_**	**28 M_1b_**	**28 M_2_**
0 nn		−7.9	−4.0	−7.8
				
0.333 nn	1–4	−4.4	−0.4	−4.1
	1–5	8.1	−2.4	−6.0
	2–5	5.2	1.5	3.2
	2–6	−6.2	−1.1	−2.5
	3–5	−2.5	−2.7	−6.1
	3–6	−3.2	8.1	5.5
	5–6	−15.3	−10.8	−14.9
				
1 nn	1–4	1.1	4.6	1.9
	1–5	22.2	−1.5	−4.4
	2–5	−^[a]^	5.2	3.2
	2–6	−^[a]^	2.5	−0.7
	3–5	1.2	−2.3	−5.3
	3–6	−^[a]^	−^[a]^	−^[a]^
	5–6	−^[b]^	−^[b]^	−^[b]^

[a] The geometry optimization of the Möbius structure converged to a different topology. [b] The geometry optimization of the Hückel structure converged to a different topology.

In the low force regime (0.333 nn), the Hückel–Möbius equilibrium evolves in favor of the Hückel structure for the first six pulling scenarios, as expected based on the analysis of the distance matrices. Indeed, these six *meso* C−C distances are larger in unperturbed **28 H** than in all of the Möbius structures and pulling to these positions was hypothesized to promote the Hückel topology. In the absence of force, the energy of the **28 M_1a_** structure, for example, is −7.9 kcal mol^−1^ relative to the energy of the **28 H** structure. When pulling with a force of 0.333 nn at the 1–4, 2–6, 3–5, and 3–6 *meso* positions, the relative energy of **28 M_1a_** is reduced to −4.4, −6.2, −2.5, and −3.2 kcal mol^−1^, respectively. For the 1–5 and 2–5 positions, the conformational equilibrium is reversed with the Hückel structure being 8.1 and 5.2 kcal mol^−1^ more stable than Möbius topology **28 M_1a_**, respectively. The same trends are observed for the **28 M_1b_** and **28 M_2_** structures: when applying a pulling force, the Möbius topologies become destabilized with respect to the Hückel structure, again confirming the expectations based on the distance matrices.

The last pulling scenario (5–6) has the opposite effect and locks the equilibrium in favor of the Möbius topology, in agreement with the C^5^−C^6^ distance matrix elements in Table 1. Therefore, the simple approach based on distance matrices seems adequate for systematically selecting pulling positions for manipulating the Hückel–Möbius equilibrium in [28]hexaphyrin mechanically. Pulling at the 2–5 positions seems to be most efficient at low force conditions because all Möbius structures are higher in energy than the Hückel conformer.

Despite the barrierless topology interconversions in the geometry optimizations for certain structures, the trend observed for the 0.333 nn pulling force survives also at 1.0 nn. For instance, in the absence of an external force, the **28 M_1a_** structure is 7.9 kcal mol^−1^ more stable than **28 H**, but becomes largely destabilized upon application of an external force at the 1–4 position with a relative energy of −4.4 kcal mol^−1^ at 0.333 nn and 1.1 kcal mol^−1^ when an external force of 1.0 nn is applied. Thus, mechanical activation in the 1.0 nn regime reverts the conformational preferences of the [28]hexaphyrin macrocycle, locking the Hückel topology. In this force regime, the 1–4 pulling scenario seems the most efficient because all Möbius structures have a higher energy than the Hückel structure. Thus, a total of two pulling scenarios invert the Hückel–Möbius equilibrium: pulling at the 2–5 and 1–4 positions. The Möbius structures appear to be more resilient to the latter pulling scenario, as not all Möbius topologies exist upon 2–5 pulling with a load of 1.0 nn.

Basis set dependency was verified for the 1–4 pulling scenario at 1.0 nn and the 5–6 pulling scenario at 0.333 nn in Table S2 in the Supporting Information. Deviations of 1 kcal mol^−1^ or smaller, with respect to the values in Table 3, were found at the M06‐2X/6–311G(d,p) and M06‐2X/6–311++G(2d,2p) levels of theory, indicating a minor influence of basis set expansion on relative Hückel–Möbius energies.

The relative enthalpy and Gibbs free energy (at 298.15 K) in the gas phase of the interconversions in Table 3 are summarized in Table S1 in the Supporting Information. The enthalpic and entropic corrections have a minor influence on the relative energy of the different conformers of [28]hexaphyrin. The (de)stabilization of the Möbius (Hückel) topology with respect to the other topology owing to an external pulling force is captured by the electronic energies because of the explicit $F_{ext}$ dependence in the EFEI formalism (Equation (1)). Any changes in (relative) thermochemical corrections are due to geometric distortions (similar to the $\Delta \Delta V_{BO}$ term in Equation (4)) and are typically small.

### 3.3 Energy distribution analysis

**Figure 5:**
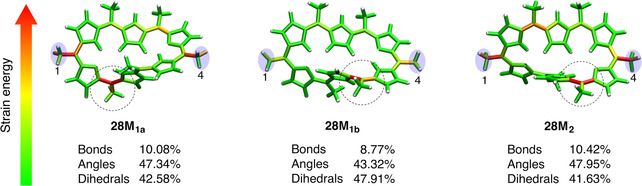
Color‐coded distribution of the external mechanical energy stored in the Möbius structures in the 1–4 pulling scenario at 1.0 nn. Red areas correspond to large amounts of stored mechanical energy, whereas green areas correspond to small ones. Circular contours highlight the activated regions around *ϕ*_1_ and *ϕ*_2_ triggering the Möbius‐to‐Hückel interconversion.

From the ab initio calculations with explicit (EFEI) force, two pulling scenarios were identified that reverse the original Hückel–Möbius conformational equilibrium in [28]hexaphyrin towards the Hückel structure, namely the 2–5 pulling at low force (0.333 nn) and the end‐to‐end 1–4 pulling at larger force (1.0 nn). To reveal the internal distribution of mechanical energy in the Möbius structures, the JEDI force distribution analysis^[6]^ was applied to the mechanically more resistant 1–4 pulling scenario in Figure 5 and to the 2–5 pulling scenario in Figure S4 in the Supporting Information.

Figure 5 illustrates the distribution of mechanical energy in the **28 M_1a_**, **28 M_1b_**, and **28 M_2_** structures in the case of 1–4 pulling with a force of 1.0 nn, as well as the fraction of the total JEDI energy that is absorbed by the bonds, angles, and dihedral angles. In each of the Möbius structures, two areas are clearly more strongly distorted than the rest of the molecule. The first area corresponds to the *meso* positions where the force is applied to (positions 1 and 4). The second area corresponds to the *meso* position near the key dihedral angles: *ϕ*_1_ for **28 M_1a_** or *ϕ*_2_ for **28 M_1b_** and **28 M_2_**. Interestingly, *ϕ*_1_ and *ϕ*_2_ define the reaction coordinate for the Hückel–Möbius interconversions.

It is important to notice that the *ϕ*_1_ and *ϕ*_2_ dihedral angles do not flip upon pulling and that the harmonic approximation behind the JEDI approach is still valid.^[6]^ In Figure S5 in the Supporting Information, the molecular region around *ϕ*_1_ and *ϕ*_2_ is also activated by using a force of 0.333 nn on the 2‐ and 5‐positions.

**Figure 6:**
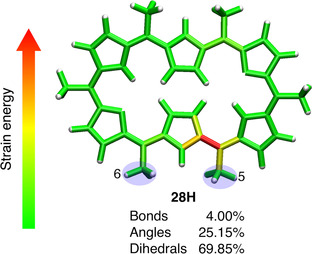
Color‐coded distribution of the external mechanical energy in the Hückel structure in the 5–6 pulling scenario at 0.333 nn. Red areas correspond to large amounts of stored mechanical energy, whereas green areas correspond to small ones.

**Figure 7:**
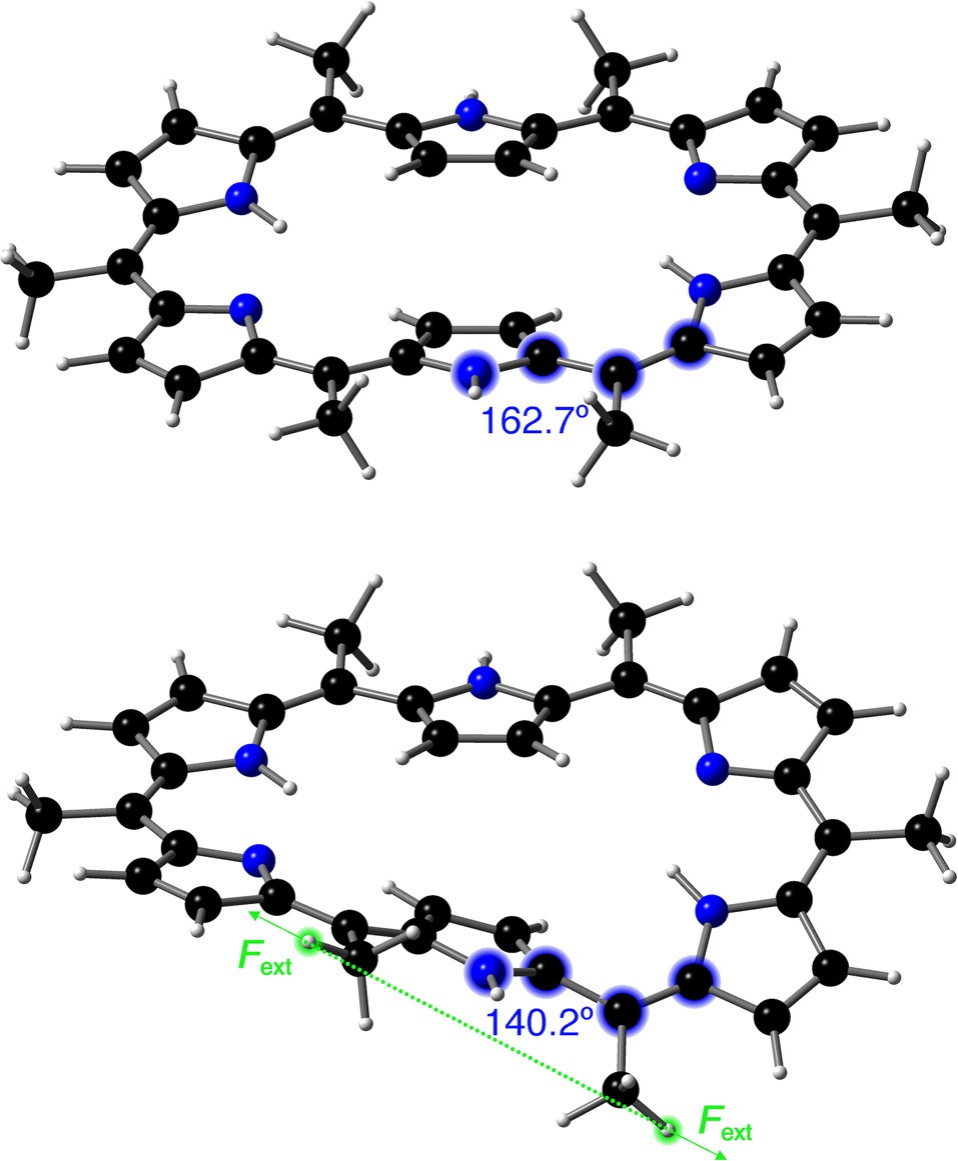
Large distortion of the *ϕ*_2_ dihedral angle (highlighted in blue) in **28 H** upon 5–6 pulling with a force of 0.333 nn.

In contrast to the 1–4 and 2–5 pulling scenarios, 5–6 pulling does not favor the Hückel structure but, instead, locks the Hückel–Möbius equilibrium in favor of the Möbius structures. The distribution of mechanical energy in Figure 6 illustrates that the Hückel structure is very locally distorted upon 5–6 pulling at 0.333 nn to trigger a Hückel‐to‐Möbius interconversion. In particular, the region near the 5‐position or the *ϕ*_2_ angle (see Figure 3) is clearly activated, indicating that the formation of **28 M_1b_** is promoted, whereas the rest of the molecule is relaxed. The force distribution in Figure 6 is not centrosymmetric owing to the loss of *C_i_* symmetry when the Hückel topology is subjected to a force acting on the 5 and 6‐positions (see above). The dihedral angles account for a significantly larger portion of the total strain energy in Figure 6 (about 70 %) than in Figure 5 (about 45 %). Figure 7 shows some dihedral angles of the Hückel structure to be largely distorted (approximately 20°), which is not apparent from the perspective in Figure 6. This large distortion indicates that the critical force for a barrierless Hückel‐to‐Möbius conversion is slightly larger than 0.333 nN. The geometry optimization of the Hückel structure did not converge to the correct topology with the larger 6‐311G(d,p) and 6‐311++G(2d,2p) basis sets, revealing a basis set dependence of the critical force (see Table S2 in the Supporting Information). Again, none of the dihedral angles flip upon pulling, justifying the application of the JEDI analysis at 0.333 nn.

### 3.4 *Meso*‐substitution

The force distribution analysis showed that an external force can efficiently trigger a Hückel‐to‐Möbius or Möbius‐to‐Hückel conversion depending on the pulling positions. In this final section, we explore the influence of the substituent on the *meso* position near *ϕ*_1_ (position 6 in Figure 3) in the **28 H**‐to‐**28 M_1a_** conversion. Table 4 lists the energy of the **28 M_1a_** structure, relative to the **28 H** energy, in the end‐to‐end 1–4 pulling scenario at 1.0 nn when the ‐CH_3_ substituent is replaced by H, *i*Pr, or Ph, while retaining the methyl substituent on all other *meso* positions. Enthalpies and Gibbs free energies are listed in Table S4 in the Supporting Information.

**Table 4:** Energy (in kcal mol^−1^) of the **28 M_1a_** structure relative to **28 H**, with different substituents on the 6‐position.

	Δ*E*(H)	Δ*E*(CH_3_)	Δ*E*(*i*Pr)	Δ*E*(Ph)
0 nn (no force)	−6.4	−7.9	−10.1	−7.5
1.0 nn	2.0	1.1	−1.3	1.9
ΔΔ*E*	8.4	9.0	8.8	9.4

In the original fully methyl‐substituted hexaphyrin, an external force of 1.0 nn shifts the **28 H**–**28 M_1a_** topological interconversions by 9.0 kcal mol^−1^, as reflected in the ΔΔ*E* values collected in Table 4, resulting in an inversion of the conformational equilibrium. Interestingly, substitution of the 6‐position by different groups does not influence the shift of the conformational equilibrium as all ΔΔ*E* values are within a range of 1 kcal mol^−1^ (8.4 kcal mol^−1^ for the H substituent up to 9.4 kcal mol^−1^ for the *i*Pr substituent). The JEDI analysis in Figure 5 shows that the mechanical energy is indeed not absorbed by the methyl substituent on the 6‐position, which is in line with the virtually unaffected ΔΔ*E* values. Thus, the thermochemistry of the **28 H**–**28 M_1a_** interconversion process without any external force is much more affected by the substituent on *meso* positions, in line with previous studies.^[44]^ In the case of a H atom, **28 M_1a_** is only favored by 6.4 kcal mol^−1^, whereas the *i*Pr‐group favors the Möbius configuration by 10.1 kcal mol^−1^, which cannot be overcome with a force of 1.0 nn. Therefore, the substituents on the *meso* positions determine whether a Hückel configuration can be forced by using mechanical stress at the molecular level.

## 4 Conclusion

Quantum mechanochemical calculations have shown that the Hückel or Möbius topology of [28]hexaphyrin can be imposed by external mechanical force, adding force to the toolbox of stimuli to trigger topological Hückel–Möbius interconversions in expanded porphyrins. The different (photo)physical properties of Hückel and Möbius structures promote these systems as conformational mechanophores.

A straightforward investigation of the distance matrices of the different conformers of [28]hexaphyrin revealed that applying an external force to certain atomic positions favors either the Hückel topology or Möbius topologies, depending on whether the interatomic distance is larger in the Hückel structure or Möbius structures, respectively. The adequacy of this approach for [28]hexaphyrin was confirmed by calculating the relative energies and explicitly including the constant external force by using the EFEI formalism. Without any exception, the equilibrium shifts indeed in favor of the structure with the larger interatomic distance, and several inversions of the original conformational equilibrium were found. A maximum force regime of 1.0 nn was put forward for this conformational mechanophore, as undesired rotations around covalent bonds might occur at higher forces. Analysis of the distribution of mechanical energy (JEDI) revealed that structural distortions occur very locally and trigger a Hückel‐to‐Möbius or Möbius‐to‐Hückel interconversion, depending on the attachment points of the external force. The substituents at the *meso* positions of the [28]hexaphyrin macrocycle were shown to affect the Hückel–Möbius equilibrium in the absence of external force, whereas the amount by which the equilibrium is shifted owing to the external force is almost invariant with different substituents. This offers a window of opportunity to fine‐tune the mechanophore for practical applications in different molecular environments, as there are, for example, many polymeric systems that can be used for polymer mechanochemical experiments.^[13b]^

## Conflict of interest

The authors declare no conflict of interest.

## Supporting information

As a service to our authors and readers, this journal provides supporting information supplied by the authors. Such materials are peer reviewed and may be re‐organized for online delivery, but are not copy‐edited or typeset. Technical support issues arising from supporting information (other than missing files) should be addressed to the authors.

Supplementary
