# Supplementary material for: Mechanochemically Triggered Topology Changes in Expanded Porphyrins
Source: Chemistry. 2021 Jan 18;27(10):3397–406. doi: 10.1002/chem.202003869 (PMC7898923; doi:10.1002/chem.202003869)
Supplement: Supplementary file 1 — Supplementary [file CHEM-27-3397-s001.pdf]

Chemistry–A European Journal

Supporting Information

Mechanochemically Triggered Topology Changes in Expanded Porphyrins

Tom Bettens,*[a] Marvin Hoffmann,[b] Mercedes Alonso,*[a] Paul Geerlings,[a]
Andreas Dreuw,*[b] and Frank De Proft*[a]

Table of contents

Figure S1. Figure-of-eight conformation of [28]hexaphyrin from two perspectives. S2

Figure S2. **28M$_{1a}$** inverted undesired dihedral angles during the geometry optimization of the 2-5, 2-6 and 3-6 pulling scenarios when a force of 1.0 nN is applied. The undesired dihedral angle inversions, with respect to the unperturbed **28M$_{1a}$** structure (top left), are indicated in red and blue ellipsoids indicate the meso positions to which the external pulling force was applied. S3

Figure S3. Contribution of the $\Delta\Delta V_{BO}$ (blue) and the $F_{ext}\Delta r$ term (orange) in equation (4) to the shift in relative Hückel-Möbius energies for the 1-4 (a), 1-5 (b), 2-5 (c), 2-6 (d), 3-5 (e) and 3-6 (f) pulling scenarios. S4

Figure S4. Contribution of the $\Delta\Delta V_{BO}$ (blue) and the $F_{ext}\Delta r$ term (orange) in equation (4) to the shift in relative Hückel-Möbius energies for the 5-6 pulling scenario............................ S5

Table S1. Enthalpies (ΔH) and Gibbs free energies (ΔG) of the Möbius conformers, relative to the Hückel topology **28H** for different force regimes computed at the M06-2X/6-311G(d,p)//M06-2X/6-31G level of theory. A positive sign indicates a more stable Hückel structure, whereas a negative sign means the opposite. All values are in kcal mol^{-1}........... S6

Table S2. Electronic energy of the Möbius topologies, relative to **28H**, computed at the M06-2X/6-311G(d,p) and M06-2X/6-311++G(2d,2p) level of theory. S7

Table S3. Wavefunction stability analysis for the M06-2X/6-311G(d,p)//M06-2X/6-31G single-point calculations. The lowest eigenvalues of the stability matrices are summarized. A negative eigenvalue indicates an instability. S7

Figure S5. Color-coded distribution of the external mechanical energy in the Möbius structures in the 2-5 pulling scenario at 0.333 nN. Red areas correspond to large amounts of stored mechanical energy, whereas green areas correspond to small ones. Circular contours highlight the activated regions around φ_1 and φ_2 triggering the Möbius-to-Hückel interconversion. S8

Table S4. Enthalpy (ΔH) and Gibbs free energy (ΔG at 298.15K) of the **28M$_{1a}$** structure relative to 28H, with different substituents on the 6-position. All values are in kcal mol^{-1}............... S8

Table S5. Cartesian coordinates of each [28]hexaphyrin structure optimized at the M06-2X/6-31G level of theory. The applied force as well as the point group symmetry are indicated.. S9

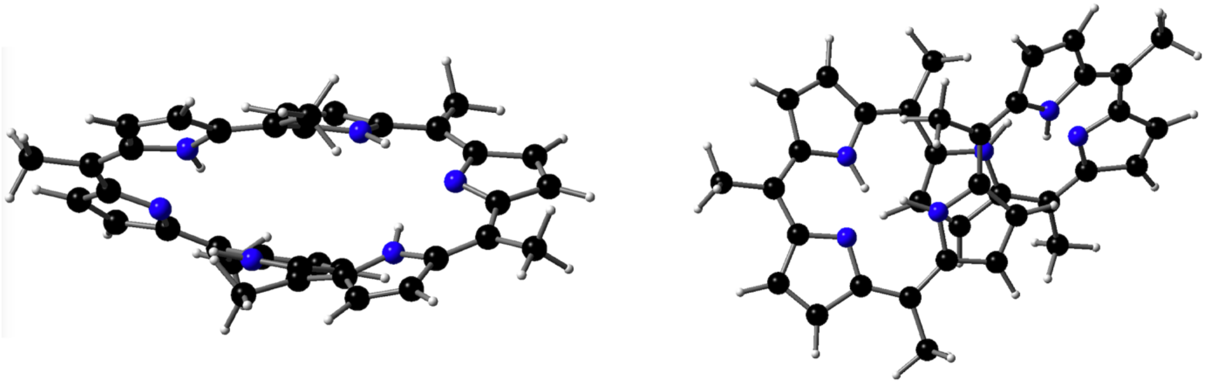

Figure S1. Figure-of-eight conformer of [28]hexaphyrin from two perspectives.

The electronic energy, enthalpy and Gibbs free energy of the Figure-of-eight conformer with respect to the planar **28H** conformer are -11.0, -11.9 and -12.0 kcal mol^{-1}, respectively.

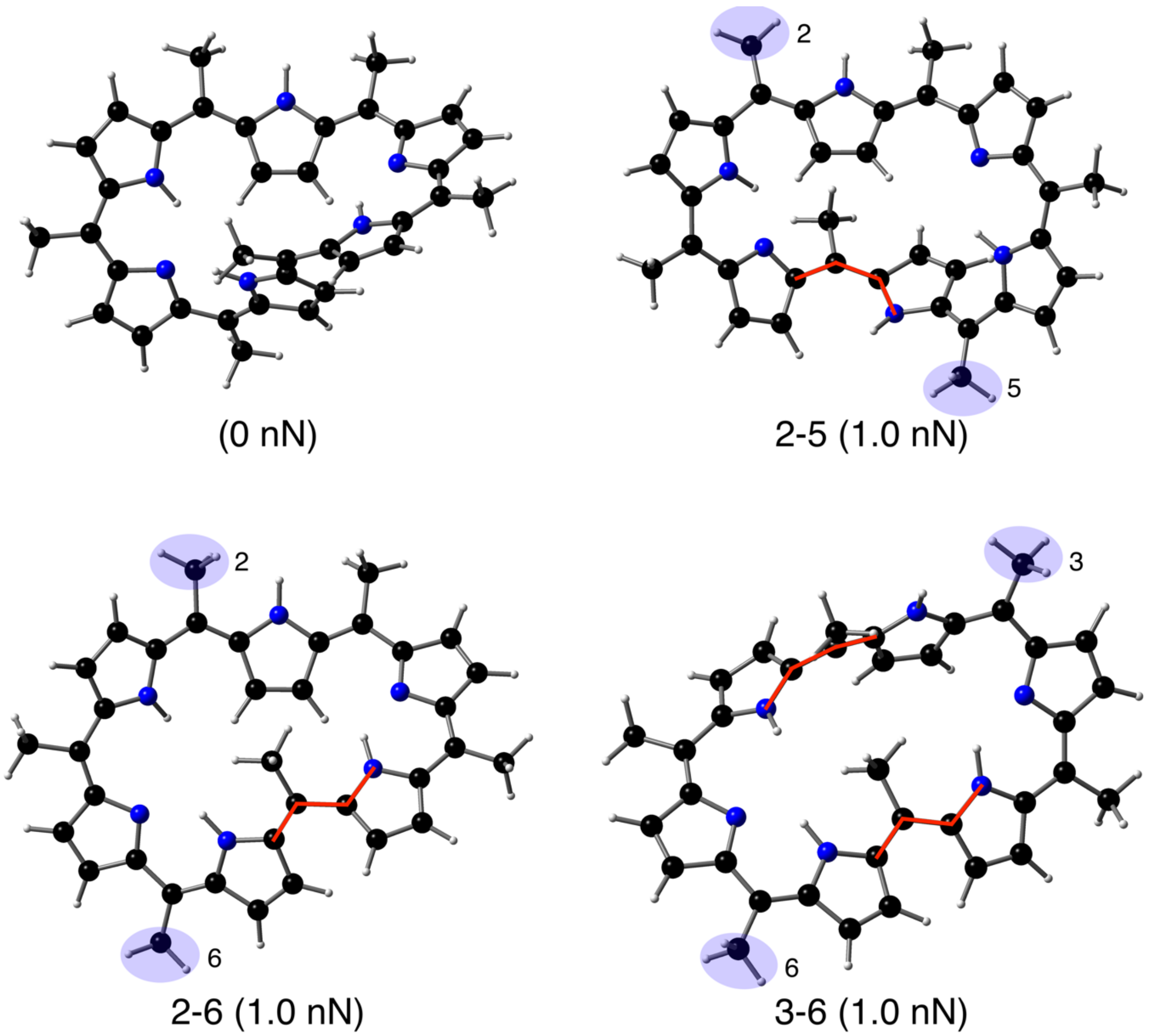

Figure S2. **$28M_{1a}$** inverted undesired dihedral angles during the geometry optimization of the 2-5, 2-6 and 3-6 pulling scenarios when a force of 1.0 nN is applied. The undesired dihedral angle inversions, with respect to the unperturbed **$28M_{1a}$** structure (top left), are indicated in red and blue ellipsoids indicate the *meso* positions to which the external pulling force was applied.

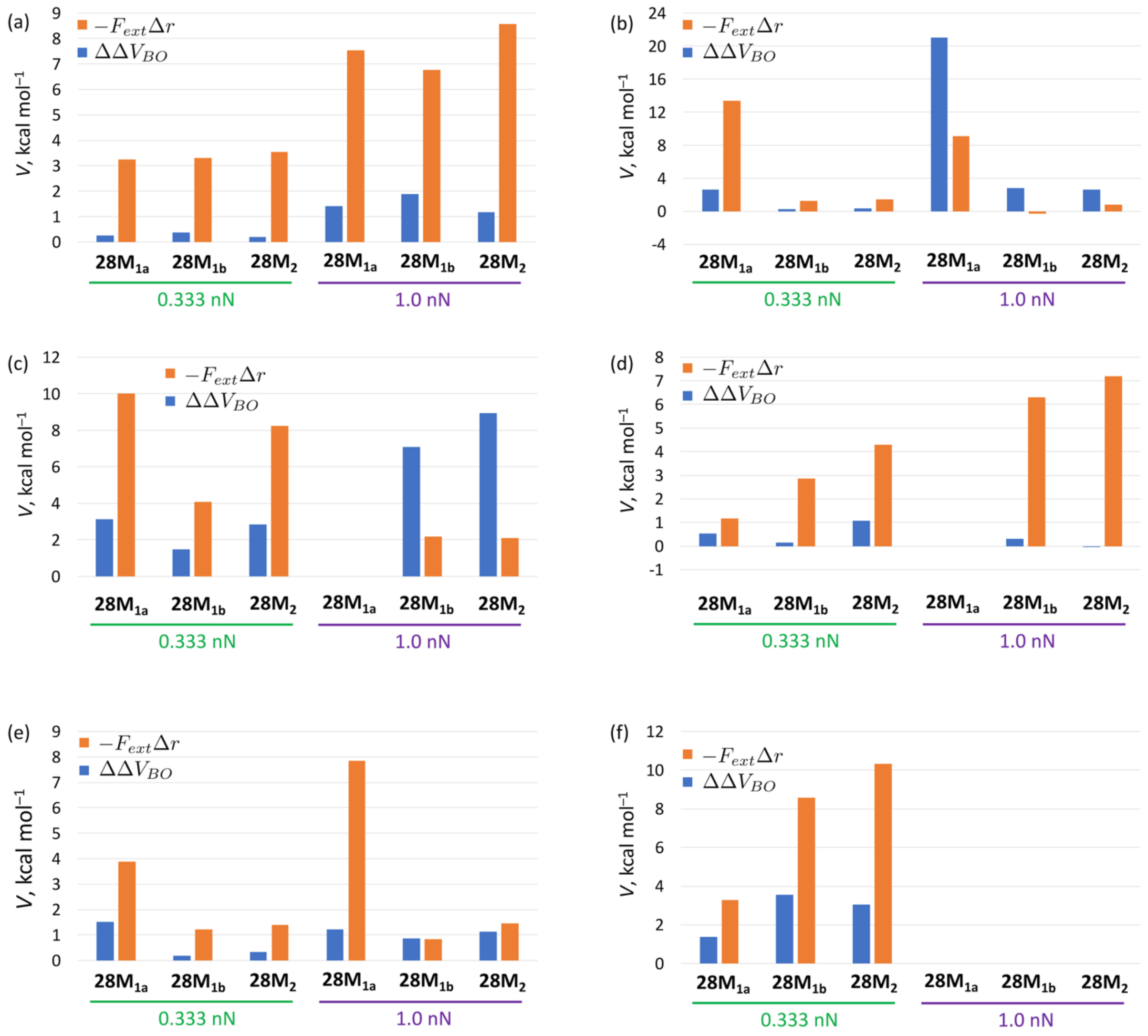

Figure S3. Contribution of the $\Delta\Delta V_{BO}$ (blue) and the $F_{ext}\ \Delta r$ term (orange) in equation (4) to the shift in relative Hückel-Möbius energies for the 1-4 (a), 1-5 (b), 2-5 (c), 2-6 (d), 3-5 (e) and 3-6 (f) pulling scenarios.

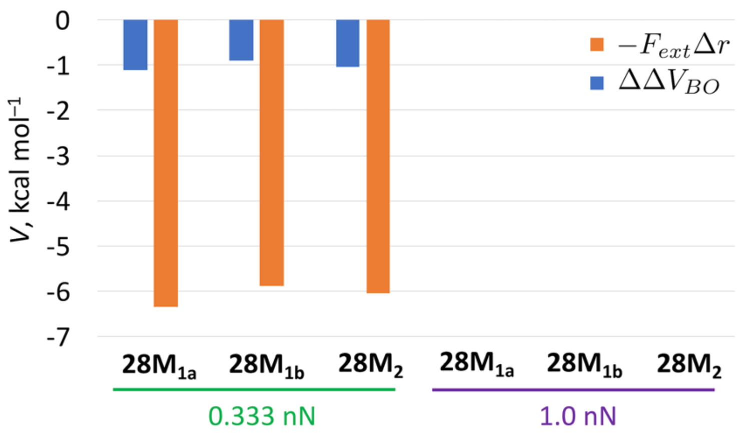

Figure S4. Contribution of the $\Delta\Delta V_{BO}$ (blue) and the $F_{ext}\ \Delta r$ term (orange) in equation (4) to the shift in relative Hückel-Möbius energies for the 5-6 pulling scenario.

Table S1. Enthalpies (ΔH) and Gibbs free energies (ΔG) of the Möbius conformers, relative to the Hückel topology **28H** for different force regimes computed at the M06-2X/6-311G(d,p)//M06-2X/6-31G level of theory. A positive sign indicates a more stable Hückel structure, whereas a negative sign means the opposite. All values are in kcal mol^{-1}.

Force	Pulling scenario	ΔH			ΔG (298.15K)		
		**28M$_{1a}$**	**28M$_{1b}$**	**28M$_2$**	**28M$_{1a}$**	**28M$_{1b}$**	**28M$_2$**
0 nN		-11.8	-8.0	-11.4	-15.3	-10.4	-14.8
0.333 nN	1-4	-8.4	-4.2	-7.7	-10.8	-5.4	-10.0
	1-5	4.5	-6.2	-9.7	5.8	-8.1	-12.6
	2-5	1.4	-1.7	-0.9	1.8	-2.3	-1.2
	2-6	-9.9	-4.5	-5.8	-12.8	-5.9	-7.6
	3-5	-6.0	-5.9	-9.2	-7.7	-7.6	-11.9
	3-6	-6.2	5.0	2.5	-8.0	6.5	3.2
	5-6	-18.2	-14.1	-17.6	-23.6	-18.3	-22.8
1.0 nN	1-4	-2.5	1.9	-1.3	-3.2	2.5	-1.7
	1-5	19.5	-4.4	-7.5	25.3	-5.7	-9.8
	2-5	-	2.8	-0.4	-	3.6	-0.5
	2-6	-	0.2	-3.1	-	0.3	-4.1
	3-5	-1.8	-4.8	-7.9	-2.3	-6.2	-10.2
	3-6	-	-	-	-	-	-
	5-6	-	-	-	-	-	-

Table S2. Electronic energy of the Möbius topologies, relative to **28H**, computed at the M06-2X/6-311G(d,p) and M06-2X/6-311++G(2d,2p) level of theory.

Pulling scenario	M06-2X/6-311G(d,p)			M06-2X/6-311++G(2d,2p)		
	**$28M_{1a}$**	**$28M_{1b}$**	**$28M_2$**	**$28M_{1a}$**	**$28M_{1b}$**	**$28M_2$**
0 nN	-8.3	-4.5	-8.2	-6.8	-3.4	-7.1
1-4 (1.0 nN)	0.7	4.2	1.6	1.7	4.5	2.1
5-6 (0.333 nN)	-	-	-	-	-	-

With the larger basis sets, the 28H conformer did not converge for the 5-6 pulling scenario at 0.333 nN. Instead, a Möbius topology was found indicating that the critical force for a barrierless Hückel-Möbius transition is slightly lower than 0.333 nN. This critical force is slightly larger than 0.333 nN at the M06-2X/6-311G(d,p)//M06-2X/6-31G level of theory, judging from the strong geometric distortion in Figure 7. The Möbius structures, on the other hand, did converge. According to Table 1, **$28M_{1a}$** is the most stable conformer under these force conditions with **$28M_{1b}$** and **$28M_2$** being respectively 4.5 and 0.4 kcal mol^{-1} higher in energy. With the 6-311G(d,p) basis, the **$28M_{1a}$** conformer is also the most stable conformer and **$28M_{1b}$** and **$28M_2$** are 4.5 and 0.4 kcal mol^{-1} higher in energy, respectively. With the 6-311++G(2d,2p) basis, the **$28M_{1a}$** conformer is also the most stable conformer and **$28M_{1b}$** and **$28M_2$** are 3.4 and 0.0 kcal mol^{-1} higher in energy, respectively. These relative energies are again in good agreement with those reported in Table 1.

Table S3. Wavefunction stability analysis for the M06-2X/6-311G(d,p)//M06-2X/6-31G single-point calculations. The lowest eigenvalues of the stability matrices are summarized. A negative eigenvalue indicates an instability.

	RKS -> UKS			RKS -> RKS			RKS -> CRKS		
	0 nN	1-4 (1.0nN)	5-6 (0.333nN)	0 nN	1-4 (1.0nN)	5-6 (0.333nN)	0 nN	1-4 (1.0nN)	5-6 (0.333nN)
**28H**	0.008	0.007	0.009	0.040	0.040	0.043	0.037	0.037	0.040
**$28M_{1a}$**	0.013	0.013	0.013	0.055	0.057	0.055	0.060	0.060	0.060
**$28M_{1b}$**	0.009	0.012	0.008	0.049	0.053	0.047	0.055	0.058	0.054
**$28M_2$**	0.010	0.008	0.009	0.050	0.048	0.049	0.052	0.051	0.052

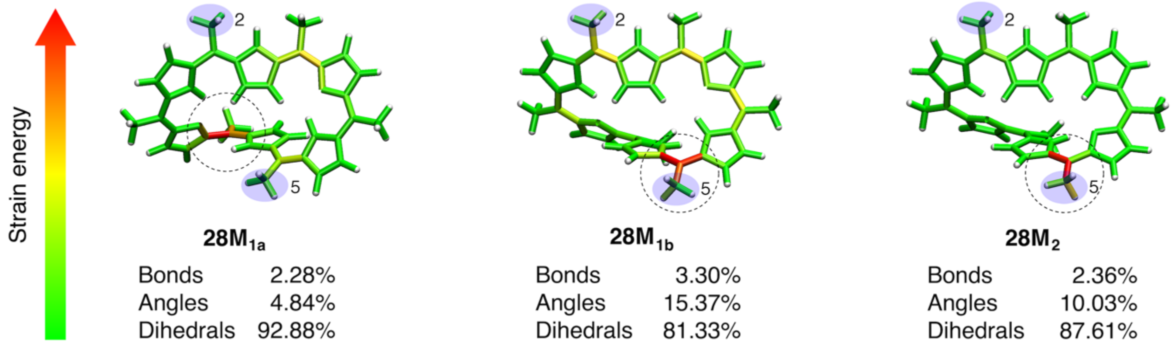

Figure S5. Color-coded distribution of the external mechanical energy in the Möbius structures in the 2-5 pulling scenario at 0.333 nN. Red areas correspond to large amounts of stored mechanical energy, whereas green areas correspond to small ones. Circular contours highlight the activated regions around φ_1 and φ_2 triggering the Möbius-to-Hückel interconversion.

Table S4. Enthalpy (ΔH) and Gibbs free energy (ΔG at 298.15K) of the **28M$_{1a}$** structure relative to **28H**, with different substituents on the 6-position. All values are in kcal mol^{-1}.

	ΔH(H)	ΔH(CH_3)	ΔH(*i*Pr)	ΔH(Ph)	ΔG(H)	ΔG(CH_3)	ΔG(*i*Pr)	ΔG(Ph)
0 nN (no Force)	-9.5	-11.8	-13.3	-10.8	-12.4	-15.3	-17.2	-14.0
1.0 nN	-0.9	-7.9	-4.6	-1.4	-1.2	-10.3	-6.0	-1.8
$\Delta\Delta H/\Delta\Delta G$	8.6	3.9	8.7	9.4	11.2	5.1	11.3	12.2

Table S5. Cartesian coordinates of each [28]hexaphyrin structure optimized at the M06-2X/6-31G level of theory. The applied force as well as the point group symmetry are indicated.

**28H** (0 nN, C_i)<br><br>N 3.927460873 1.434867286 -0.05146257<br>N 0.054512494 3.548816426 0.071661201<br>N -3.842199202 1.430263253 -0.039909728<br>N -3.927460873 -1.434867286 0.05146257<br>N -0.054512494 -3.548816426 -0.071661201<br>N 3.842199202 -1.430263253 0.039909728<br>C 5.901744427 -0.036967254 0.117880386<br>C 5.296297124 1.185998518 0.05179232<br>C 5.937099341 2.49705312 0.05575006<br>C 4.983458652 3.447574836 -0.035242597<br>C 3.667573551 2.807975822 -0.100267654<br>C 2.467418373 3.458440353 -0.156397156<br>C 1.174754721 2.814411189 -0.238685159<br>C 0.714927912 1.549410582 -0.643140133<br>C -0.693418698 1.550482203 -0.563025045<br>C -1.108804445 2.807862267 -0.106130243<br>C -2.378494083 3.439003672 0.102667221<br>C -3.606054822 2.819985383 0.070355631<br>C -4.889755893 3.516193264 0.123090247<br>C -5.857115303 2.573054435 0.026019784<br>C -5.176658703 1.280823358 -0.058570942<br>C -5.901744427 0.036967254 -0.117880386<br>C -5.296297124 -1.185998518 -0.05179232<br>C -5.937099341 -2.49705312 -0.05575006<br>C -4.983458652 -3.447574836 0.035242597<br>C -3.667573551 -2.807975822 0.100267654<br>C -2.467418373 -3.458440353 0.156397156<br>C -1.174754721 -2.814411189 0.238685159<br>C -0.714927912 -1.549410582 0.643140133<br>C 0.693418698 -1.550482203 0.563025045<br>C 1.108804445 -2.807862267 0.106130243<br>C 2.378494083 -3.439003672 -0.102667221<br>C 3.606054822 -2.819985383 -0.070355631<br>C 4.889755893 -3.516193264 -0.123090247<br>C 5.857115303 -2.573054435 -0.026019784<br>C 5.176658703 -1.280823358 0.058570942<br>H 3.269427893 0.663587414 -0.03296217<br>H 0.083464247 4.488926153 0.424677955<br>H -3.269427893 -0.663587414 0.03296217<br>H -0.083464247 -4.488926153 -0.424677955<br>H 6.999708779 2.664014947 0.11516198<br>H 5.152575843 4.510844887 -0.05976486

H 1.324316935 0.738020219 -1.009523568<br>H -1.360488286 0.751064036 -0.836768323<br>H -5.045351508 4.580316335 0.193926564<br>H -6.924254963 2.728341311 0.021904093<br>H -6.999708779 -2.664014947 -0.11516198<br>H -5.152575843 -4.510844887 0.05976486<br>H -1.324316935 -0.738020219 1.009523568<br>H 1.360488286 -0.751064036 0.836768323<br>H 5.045351508 -4.580316335 -0.193926564<br>H 6.924254963 -2.728341311 -0.021904093<br>C 7.40567473 -0.141673214 0.226763053<br>C 2.423953514 4.971513361 -0.093745015<br>C -2.304719508 4.929185386 0.369368146<br>C -7.40567473 0.141673214 -0.226763053<br>C -2.423953514 -4.971513361 0.093745015<br>C 2.304719508 -4.929185386 -0.369368146<br>H 7.69222366 -0.754812671 1.087825057<br>H 7.837967033 -0.608260654 -0.665956809<br>H 7.878496391 0.831511348 0.349921024<br>H 1.754737566 5.371257782 -0.863725714<br>H 2.066063479 5.326958707 0.883038332<br>H 3.400783679 5.42256708 -0.251607008<br>H -1.775870531 5.12146993 1.311587751<br>H -1.766420926 5.443597075 -0.434238653<br>H -3.284847399 5.385921936 0.461598617<br>H -7.837967033 0.608260654 0.665956809<br>H -7.69222366 0.754812671 -1.087825057<br>H -7.878496391 -0.831511348 -0.349921024<br>H -2.066063479 -5.326958707 -0.883038332<br>H -1.754737566 -5.371257782 0.863725714<br>H -3.400783679 -5.42256708 0.251607008<br>H 1.766420926 -5.443597075 0.434238653<br>H 1.775870531 -5.12146993 -1.311587751<br>H 3.284847399 -5.385921936 -0.461598617
**28M$_{1a}$** (0 nN, C_1)<br><br>N -3.3243096946 1.5431377766 0.5876870750<br>N 0.5850646036 3.3337281998 -0.572106863<br>N 3.8849581348 0.6729887104 -0.3858238866<br>N 3.0634848154 -1.4041314435 0.9630035922<br>N -1.0296394271 -1.9965563092 -0.0632007752<br>N -3.6429396615 -0.9861935554 -0.3539844337<br>C -5.3632439329 0.1840019059 0.9404962397<br>C -4.5444892045 1.3118511069 1.2110231655<br>C -4.8438699589 2.4781713184 1.9463966838

C -3.8177116007 3.3961904143 1.7329890281
C -2.8631464652 2.8022989953 0.8682233734
C -1.6573592471 3.4000567194 0.3628770567
C -0.5906566753 2.7024933100 -0.1491095648
C -0.3201792431 1.2773090764 -0.2902824097
C 0.9660478360 1.1091899118 -0.7014046565
C 1.5947031890 2.4088521822 -0.8586610603
C 2.8830080763 2.7600054255 -1.1827457695
C 3.9832141873 1.8404266417 -1.0388688522
C 5.3798434034 2.1001285512 -1.379977488
C 6.1123204698 1.0598281376 -0.8821853134
C 5.1766954344 0.1599282285 -0.2280629404
C 5.4407490949 -0.9372563069 0.5821594232
C 4.3955246178 -1.6584211580 1.2263625921
C 4.4175762439 -2.6478889885 2.2353988887
C 3.0914624970 -2.9454450022 2.5757102237
C 2.2504528396 -2.1706806164 1.7440605089
C 0.8134912093 -2.1479064917 1.5646757653
C 0.3077180964 -2.1552355095 0.2907324958
C 0.9892224360 -2.5205644808 -0.9524006607
C 0.0681962308 -2.6375431744 -1.9411687046
C -1.2488874410 -2.3094577620 -1.4076502765
C -2.4775570099 -2.3672959863 -2.0128493677
C -3.6596338387 -1.9302305208 -1.3168512362
C -5.0094673569 -2.4306796221 -1.5137325098
C -5.8046763875 -1.7910079478 -0.6027053917
C -4.9597897472 -0.8444903177 0.1015343684
H -2.9482701204 0.8326351280 -0.0373825316
H 0.7571681822 4.3176770131 -0.4597117739
H 2.8298241734 -0.5844886703 0.3869721824
H -1.7854094899 -1.6674807839 0.5224728999
H -5.7226127484 2.6265844216 2.5538154545
H -3.7414860020 4.3813631145 2.1626053698
H -1.0200024710 0.4772966726 -0.1091867376
H 1.4513106755 0.1783522235 -0.9401806611
H 5.7579130445 2.9564773408 -1.9162311076
H 7.1825479484 0.9357764296 -0.9485684036
H 5.3049639211 -3.0891016773 2.6624157459
H 2.7574556917 -3.6780058424 3.2931025191
H 2.0476719167 -2.7180732374 -1.0179708813
H 0.2597066411 -2.9308636970 -2.9607814126
H -5.3041917319 -3.1854092233 -2.2266763774
H -6.8683260044 -1.9180973148 -0.4686759053
C -6.7617140329 0.2069953925 1.5040976546
C -1.5391011911 4.9058036965 0.4511502691
C 3.1881569111 4.2043129110 -1.5011156105
C 6.8530657811 -1.3658613704 0.8801017233

C -0.0858037789 -2.1199154413 2.7705991027<br>C -2.6494616370 -2.9292313679 -3.4013862388<br>H -6.7349102664 0.4277479317 2.5756378631<br>H -7.3688190008 0.9819542612 1.0244297951<br>H -7.2576088827 -0.7545553297 1.3764545345<br>H -0.8361649950 5.2182121977 1.2343968182<br>H -1.1878379458 5.3215960914 -0.5010716242<br>H -2.5033439980 5.3687033065 0.6588055036<br>H 3.4402083422 4.7790424748 -0.5997963556<br>H 4.0380954235 4.2858980500 -2.1813013481<br>H 2.3368270809 4.6885187272 -1.9899032024<br>H 6.9646994455 -2.4458934985 0.7460925693<br>H 7.5734507300 -0.8643589473 0.2348697255<br>H 7.1116347260 -1.1357740509 1.9197902959<br>H -0.3240958469 -1.0910236196 3.0697295219<br>H -1.0281919519 -2.6433943016 2.5808140263<br>H 0.4051098712 -2.5945655201 3.6246953307<br>H -3.2421786927 -3.8516867170 -3.3794624608<br>H -3.1708667405 -2.2221837970 -4.054465278<br>H -1.6915314409 -3.1716158050 -3.8630046351
**28M$_{\mathbf{1b}}$** (0 nN, C$_1$)<br><br>N 3.9632781932 0.5749540735 -0.5367127799<br>N 0.7165364440 3.2117553249 -0.5607259253<br>N -3.1891263597 1.5466199026 0.6419321590<br>N -3.7579231379 -0.9119072558 -0.3594300733<br>N -1.0865683925 -2.0987615373 -0.0573076706<br>N 3.0345131168 -1.4963258360 0.7898728741<br>C 5.4235889983 -0.9758061858 0.6362309367<br>C 5.2203375238 0.0773477331 -0.2339619542<br>C 6.1593160771 1.0049461900 -0.8472334966<br>C 5.4530658294 2.0240415199 -1.4134471516<br>C 4.0306284306 1.7927578997 -1.1862480278<br>C 2.9624102166 2.6425884956 -1.3855644574<br>C 1.6415688720 2.2726736035 -0.9692113738<br>C 1.0155919565 1.0143585643 -0.8260164851<br>C -0.2655083597 1.2159588263 -0.3099040644<br>C -0.4601896058 2.6003555950 -0.119296816<br>C -1.4985971142 3.3521961215 0.4877874284<br>C -2.7175559900 2.8320249182 0.9101370714<br>C -3.7362858223 3.5636329855 1.6551851132<br>C -4.7963898869 2.7252520175 1.8133473225<br>C -4.4472409038 1.4785425580 1.1460591902<br>C -5.3703000399 0.4096898954 0.9177686656<br>C -5.0393004563 -0.6681524706 0.1251279695<br>C -5.8970973877 -1.6536718863 -0.5062713673

C -5.1561272138 -2.3672391616 -1.400648443
C -3.7739743376 -1.9347038283 -1.3111606622
C -2.6638032354 -2.4479833568 -1.9416767568
C -1.3462347094 -2.2919658341 -1.4022018936
C -0.0966141109 -2.4864441376 -2.0263106044
C 0.8979440095 -2.3993488135 -1.0525617768
C 0.2780328660 -2.1698400413 0.1969481173
C 0.8288706934 -2.2382495475 1.5081664442
C 2.1913271513 -2.0953518592 1.7198757775
C 2.9921143339 -2.5938864164 2.8304393908
C 4.2986787429 -2.3458028643 2.5169089533
C 4.2976238879 -1.6164630842 1.2520831609
H 3.1460091583 0.0866816464 -0.1667354931
H 0.9047806457 4.1985979522 -0.5076891173
H -3.0657865458 -0.1612812573 -0.2598868315
H -1.8103931256 -1.9665117601 0.6342419546
H 7.2331824392 0.9058303269 -0.8238163354
H 5.8650408464 2.8815247304 -1.9212873629
H 1.4344396811 0.0637957227 -1.1111211935
H -0.9915451239 0.4609134808 -0.0688206533
H -3.6612172294 4.5729980266 2.0272106751
H -5.7291535020 2.9356506575 2.3133538674
H -6.9524298445 -1.7667869171 -0.3154773917
H -5.5036652348 -3.1737339981 -2.0269188592
H 0.0459628980 -2.6743887647 -3.078368435
H 1.9626746932 -2.5043503511 -1.1783812209
H 2.6219514615 -3.1171108639 3.6992302378
H 5.1718150293 -2.6006710902 3.0987513005
C 6.8098817895 -1.3783490279 1.0681463517
C 3.1726349231 4.0504692891 -1.8859497389
C -1.2090288543 4.8257908081 0.6743800562
C -6.7752051539 0.5596247231 1.4488000190
C -2.7947995360 -3.2778668528 -3.1935490894
C -0.1093208098 -2.5331774622 2.6492961245
H 7.5768417525 -0.9071944536 0.4510221398
H 7.0020944443 -1.0935337975 2.1097674828
H 6.9443569170 -2.4623323752 0.9963974258
H 3.1477803755 4.7831047954 -1.0664253902
H 4.1387332456 4.1614660803 -2.379859731
H 2.3967429323 4.3293721562 -2.6064748796
H -0.3197856222 4.9770233220 1.2975627234
H -1.0254501178 5.3031919455 -0.2964753428
H -2.0374144452 5.3558943317 1.1360240962
H -6.7628220151 0.7591545391 2.5254077296
H -7.3038025707 1.3907268269 0.9682903540
H -7.3615243837 -0.3471855473 1.2952218634
H -2.4069695418 -4.2918369279 -3.0510754287

H -3.8335094675 -3.3404102706 -3.521435511<br>H -2.2267723057 -2.8240018085 -4.0141867219<br>H -0.8071353374 -1.6972860383 2.7905332749<br>H -0.7028370990 -3.4343247457 2.4618757064<br>H 0.4308293239 -2.6561108790 3.5877473799
**28M₂** (0 nN, C_1)<br><br>N 3.9311687808 0.5438478526 -0.5908602744<br>N 0.7010632103 3.1737094960 -0.6562741841<br>N -3.1777167971 1.5643575443 0.6926831223<br>N -3.6477604828 -0.9594619807 -0.2852657380<br>N -1.0926737824 -2.0524650155 -0.0646112584<br>N 2.9872208147 -1.4500177057 0.8297946738<br>C 5.3759546272 -0.9359772665 0.6873214912<br>C 5.1820564099 0.0699693747 -0.2450514081<br>C 6.1280171995 0.9726577722 -0.8841305440<br>C 5.4264542955 1.9633998017 -1.5061681032<br>C 4.0034518718 1.7379288050 -1.2883433582<br>C 2.9349764728 2.5791517657 -1.5142915507<br>C 1.6147854561 2.2212986239 -1.0813160538<br>C 0.9864126167 0.9752801424 -0.9116885953<br>C -0.2839545925 1.1904365126 -0.3610103326<br>C -0.4612151980 2.5769814900 -0.1754467098<br>C -1.4722554200 3.3533905261 0.4689717551<br>C -2.6707344578 2.8404162902 0.9247441112<br>C -3.6751719910 3.5053472475 1.7344432690<br>C -4.7152354536 2.6499608068 1.9452966114<br>C -4.4376093433 1.4097759949 1.2505210447<br>C -5.2874023004 0.3431686976 1.0087872902<br>C -4.9176501309 -0.6948319963 0.1080898032<br>C -5.8625419511 -1.4998719063 -0.6636382682<br>C -5.1351179884 -2.1874743554 -1.5871495799<br>C -3.7333347935 -1.879442439 -1.3372361528<br>C -2.6269519423 -2.4167182654 -1.9681553592<br>C -1.3244792970 -2.3387913749 -1.3963541769<br>C -0.0695528370 -2.6501872071 -1.9668575091<br>C 0.8996206906 -2.5239757654 -0.9753419163<br>C 0.2502147515 -2.1611152367 0.2317100920<br>C 0.7720363287 -2.1409701142 1.5627433888<br>C 2.1271762950 -1.9850735734 1.7850595964<br>C 2.9124387731 -2.4061967094 2.9432555505<br>C 4.2215698709 -2.1839544234 2.6331453267<br>C 4.2427537336 -1.5378035825 1.3186399943<br>H 3.1090272262 0.0702154994 -0.2111342987<br>H 0.8961700664 4.1596718700 -0.6111819505<br>H -2.8191076798 0.8400207527 0.0778614635

H -1.8473286653 -1.7267233738 0.5315998566<br>H 7.2016531266 0.8808051554 -0.8360543782<br>H 5.8435979227 2.8004264670 -2.0433517089<br>H 1.3901897214 0.0158397727 -1.1926693647<br>H -0.9771538795 0.4088013833 -0.1023890336<br>H -3.5951903509 4.5094589176 2.1178105037<br>H -5.6123482406 2.8541076889 2.5069909151<br>H -6.9355574807 -1.4953269552 -0.5441365270<br>H -5.5106020893 -2.8707039831 -2.3335098393<br>H 0.0930379869 -2.9369369655 -2.9935788406<br>H 1.9607569024 -2.6892104799 -1.0565053361<br>H 2.5261262720 -2.8667817278 3.8400086651<br>H 5.0858590751 -2.4004867893 3.2432173661<br>C 6.7578510162 -1.2982681275 1.1682477955<br>C 3.1444961143 3.9704069063 -2.0589627272<br>C -1.1595113304 4.8180804475 0.6718162547<br>C -6.6987813336 0.3686218474 1.5469820065<br>C -2.7510809295 -3.1586641658 -3.2736331861<br>C -0.1949606898 -2.3628266263 2.6953867142<br>H 7.5295165053 -0.8983426519 0.5078598965<br>H 6.9496130347 -0.9075443397 2.1752011997<br>H 6.8881576769 -2.3841969310 1.2083658595<br>H 3.1170617040 4.7274149410 -1.2621246761<br>H 4.1117772096 4.0670316069 -2.5536192780<br>H 2.3686012854 4.2253967194 -2.7878551278<br>H -0.2474585821 4.9518077421 1.2659676230<br>H -1.0085517667 5.3189087546 -0.2933491596<br>H -1.9662396069 5.3460469086 1.1761327547<br>H -6.7577431048 0.9290955756 2.4823893793<br>H -7.4009418337 0.8278708796 0.8398731152<br>H -7.0519184426 -0.6448221051 1.7538760189<br>H -3.7727206578 -3.1352963511 -3.6517680415<br>H -2.1101650371 -2.6965079089 -4.0327726087<br>H -2.4354539194 -4.2020368767 -3.1722424567<br>H -0.8952684824 -1.5201593189 2.7649825525<br>H -0.7882690549 -3.2707052298 2.5456799514<br>H 0.3204327721 -2.4287333948 3.6536949293
**28H$_{f8}$** (Figure-of-eight conformer, 0 nN, C$_1$)<br><br>N 0.613140551 -3.698748534 -0.484167564<br>C 1.211489836 -3.822344834 -1.73466572<br>C 1.669770234 -2.685204278 -2.376940354<br>C 1.725783615 -1.419655187 -1.700003087<br>C 1.859198423 -0.114379526 -2.209205566<br>C 1.848390284 0.782326434 -1.131782742<br>C 1.740447773 0.028611319 0.061690116

C 1.857092744 0.387706824 1.465066767
C 1.66062111 1.674468254 1.89205654
C 2.032374449 2.35067001 3.137632219
C 1.719566449 3.671128344 3.031563961
C 1.063972116 3.904043774 1.742134601
C 0.507597518 5.035219107 1.184200372
C -0.220706194 4.903932916 -0.061124481
N -0.611780037 3.698578857 -0.485066523
C -1.210232299 3.822185218 -1.735508963
C -1.668707877 2.685153741 -2.377856448
C -1.725047791 1.419491766 -1.701059943
C -1.858666682 0.11425981 -2.210321041
C -1.848471274 -0.78249937 -1.13291661
C -1.740641107 -0.028857367 0.060602123
C -1.857720629 -0.387855384 1.463973714
C -1.66141601 -1.674558814 1.891187871
C -2.033451893 -2.350579739 3.136778595
C -1.720568492 -3.671038923 3.030979078
C -1.064293934 -3.904051609 1.741911533
C -0.507470169 -5.035205447 1.184446754
C 0.221378976 -4.904012683 -0.060611854
C 0.605062309 -5.905070929 -1.053648232
C 1.193310947 -5.231525298 -2.095471933
N 1.661590637 -1.283905571 -0.32066469
N 1.072374761 2.661463646 1.121011361
C -0.604672166 5.905037694 -1.054008739
C -1.192277854 5.23141873 -2.096146614
N -1.661314275 1.283636351 -0.321708088
N -1.072884295 -2.661632323 1.120467321
H 0.430359578 -6.968382301 -0.983939388
H 1.589399619 -5.665934229 -3.001266356
H 1.48607041 -2.092576923 0.265915423
H 1.942515961 0.146349942 -3.253048139
H 1.952553276 1.853366557 -1.193813177
H 0.547111416 2.533755466 0.259981082
H 2.530370758 1.877336373 3.969423201
H 1.902760543 4.431975475 3.774162132
H -0.430461872 6.968413902 -0.98402411
H -1.588360451 5.665797651 -3.001958357
H -1.485615492 2.092158479 0.2649975
H -1.941744633 -0.146470485 -3.254178265
H -1.952966044 -1.853501136 -1.195027378
H -0.547333442 -2.533903991 0.259666583
H -2.531749509 -1.877157377 3.968339369
H -1.903948784 -4.431795771 3.77362442
C -2.122857774 2.724659317 -3.810444944
H -2.023916565 3.725746455 -4.231001366

H -1.518376178 2.042537687 -4.418589516
H -3.165284706 2.40409831 -3.900444187
C 0.627716883 6.392768792 1.820767
H 1.202740626 6.353913073 2.747947961
H 1.135253937 7.096994386 1.15037253
H -0.35593518 6.817088183 2.051271436
C 2.241114582 -0.709582852 2.423830963
H 2.386680147 -0.324255913 3.434248955
H 1.447179195 -1.46782189 2.482811884
H 3.159831585 -1.217381932 2.110527042
C 2.123238693 -2.724286292 -3.809777086
H 2.028126568 -3.726169901 -4.229320097
H 1.515492806 -2.045285308 -4.418201705
H 3.164261346 -2.399554976 -3.900768385
C -0.627663575 -6.392665745 1.821178224
H -1.203747432 -6.353900728 2.747704363
H -1.134124515 -7.097248877 1.150350246
H 0.35592834 -6.816491893 2.05284792
C -2.241769005 0.709593191 2.422531608
H -2.387528147 0.324415259 3.432978899
H -1.447767165 1.467764141 2.481524849
H -3.160395559 1.217428921 2.109022035

28H (0.333 nN at 1-4, C_i)

N 3.95534972 1.428255422 -0.042903689
N 0.055158073 3.519838092 0.079968036
N -3.866027938 1.40657013 -0.052044726
N -3.95534972 -1.428255422 0.042903689
N -0.055158073 -3.519838092 -0.079968036
N 3.866027938 -1.40657013 0.052044726
C 5.940859362 -0.028840988 0.10333262
C 5.328368995 1.191481522 0.044224905
C 5.957115231 2.507471136 0.047501112
C 4.993139499 3.449852683 -0.025081881
C 3.680884834 2.798522739 -0.082033062
C 2.473396856 3.438629622 -0.130709575
C 1.181383784 2.790667968 -0.225521742
C 0.727214754 1.52933535 -0.647382296
C -0.682324207 1.526839288 -0.581952601
C -1.106232647 2.778412349 -0.117242931
C -2.380825433 3.407276214 0.079586964
C -3.613384795 2.795061102 0.042908834
C -4.891236279 3.504020322 0.087695856
C -5.868799315 2.569956166 0.006248999
C -5.203508951 1.269332325 -0.063816832
C -5.940859362 0.028840988 -0.10333262

C -5.328368995 -1.191481522 -0.044224905<br>C -5.957115231 -2.507471136 -0.047501112<br>C -4.993139499 -3.449852683 0.025081881<br>C -3.680884834 -2.798522739 0.082033062<br>C -2.473396856 -3.438629622 0.130709575<br>C -1.181383784 -2.790667968 0.225521742<br>C -0.727214754 -1.52933535 0.647382296<br>C 0.682324207 -1.526839288 0.581952601<br>C 1.106232647 -2.778412349 0.117242931<br>C 2.380825433 -3.407276214 -0.079586964<br>C 3.613384795 -2.795061102 -0.042908834<br>C 4.891236279 -3.504020322 -0.087695856<br>C 5.868799315 -2.569956166 -0.006248999<br>C 5.203508951 -1.269332325 0.063816832<br>H 3.306025608 0.64858007 -0.02141534<br>H 0.078657522 4.456651651 0.441958378<br>H -3.306025608 -0.64858007 0.02141534<br>H -0.078657522 -4.456651651 -0.441958378<br>H 7.019594234 2.680128851 0.097787614<br>H 5.152868372 4.514704587 -0.044220853<br>H 1.34289589 0.721905539 -1.012181433<br>H -1.34371345 0.727343411 -0.869786495<br>H -5.037470972 4.570287691 0.143621437<br>H -6.93402631 2.737700258 0.001678132<br>H -7.019594234 -2.680128851 -0.097787614<br>H -5.152868372 -4.514704587 0.044220853<br>H -1.34289589 -0.721905539 1.012181433<br>H 1.34371345 -0.727343411 0.869786495<br>H 5.037470972 -4.570287691 -0.143621437<br>H 6.93402631 -2.737700258 -0.001678132<br>C 7.44964157 -0.147741076 0.180587742<br>C 2.419616654 4.951112762 -0.053808343<br>C -2.304969118 4.898696732 0.343432092<br>C -7.44964157 0.147741076 -0.180587742<br>C -2.419616654 -4.951112762 0.053808343<br>C 2.304969118 -4.898696732 -0.343432092<br>H 7.744573245 -0.743203842 1.051662519<br>H 7.845429773 -0.654592187 -0.707338551<br>H 7.968160472 0.811241128 0.258691023<br>H 1.75440919 5.354512216 -0.825387206<br>H 2.050992801 5.294065561 0.923453683<br>H 3.394696097 5.410273402 -0.197889766<br>H -1.787802015 5.090811496 1.292196799<br>H -1.753792966 5.4086695 -0.454130151<br>H -3.283708977 5.360302711 0.422541586<br>H -7.845429773 0.654592187 0.707338551<br>H -7.744573245 0.743203842 -1.051662519

H -7.968160472 -0.811241128 -0.258691023<br>H -2.050992801 -5.294065561 -0.923453683<br>H -1.75440919 -5.354512216 0.825387206<br>H -3.394696097 -5.410273402 0.197889766<br>H 1.753792966 -5.4086695 0.454130151<br>H 1.787802015 -5.090811496 -1.292196799<br>H 3.283708977 -5.360302711 -0.422541586
**28H** (0.333 nN at 1-5, C_1)<br><br>N 3.949914566 -1.44890721 0.077466066<br>N 0.074495558 -3.525156426 -0.273558291<br>N -3.817868378 -1.426011451 0.089935262<br>N -3.933692474 1.438789994 0.018798215<br>N -0.084580455 3.581587884 -0.076107707<br>N 3.837419447 1.408303917 0.025438804<br>C 5.919747482 0.032360287 0.123215107<br>C 5.321032976 -1.195458127 0.102429359<br>C 5.965702191 -2.504276505 0.083520582<br>C 5.011805431 -3.457784617 0.024794482<br>C 3.692917333 -2.821550407 0.021989587<br>C 2.489854703 -3.466933454 -0.034370792<br>C 1.201039591 -2.822510508 0.083339163<br>C 0.750507741 -1.591754152 0.592919251<br>C -0.658319147 -1.582289878 0.531051029<br>C -1.083741501 -2.798957994 -0.019036846<br>C -2.358667355 -3.417356011 -0.231120775<br>C -3.584416532 -2.806737063 -0.105027344<br>C -4.865829332 -3.508481792 -0.112128787<br>C -5.828301748 -2.578730946 0.099178239<br>C -5.148974563 -1.287301131 0.201094917<br>C -5.875371536 -0.050663896 0.340767527<br>C -5.288477713 1.177390139 0.223522135<br>C -5.945422134 2.480209203 0.233484218<br>C -5.01576266 3.437121714 0.030419957<br>C -3.697506385 2.811666989 -0.102520339<br>C -2.513145262 3.476717882 -0.256031667<br>C -1.213995354 2.837520452 -0.331143731<br>C -0.766092541 1.54193714 -0.643234561<br>C 0.641936457 1.53203282 -0.557406865<br>C 1.073475888 2.812418515 -0.191317983<br>C 2.35497393 3.428650083 0.00654503<br>C 3.582131707 2.801658142 0.058334449<br>C 4.859036123 3.508147795 0.161069924<br>C 5.836140284 2.570984269 0.180331598<br>C 5.173859938 1.26970222 0.104275644<br>H 3.293405796 -0.677097155 0.024475771

H 0.09415187 -4.438216539 -0.691986093
H -3.267300784 0.675271094 0.003120479
H -0.106107481 4.542796825 0.216628239
H 7.030401611 -2.666951004 0.108537858
H 5.182210963 -4.521027044 0.003564949
H 1.368408218 -0.814984509 1.015865629
H -1.319644997 -0.805914172 0.876981761
H -5.020219343 -4.569386803 -0.223720248
H -6.892196991 -2.741615856 0.166589423
H -7.003060885 2.636398409 0.366629766
H -5.202003674 4.496559321 -0.01918411
H -1.381233952 0.711363025 -0.952978707
H 1.29792335 0.704797861 -0.767395814
H 5.007641082 4.574761829 0.195491351
H 6.899793482 2.737153918 0.242468224
C 7.430023286 0.14965627 0.169431564
C 2.441092703 -4.96631587 -0.237837813
C -2.294886635 -4.88548499 -0.601420895
C -7.365831714 -0.168029632 0.563628704
C -2.506772007 4.993582348 -0.294072103
C 2.289987615 4.935156111 0.17654974
H 7.749885447 0.686720824 1.069642975
H 7.941409809 -0.817423132 0.174744568
H 7.805339219 0.710329011 -0.694113591
H 1.798284526 -5.443022338 0.511123097
H 2.048062632 -5.222211903 -1.232051456
H 3.42286549 -5.428457641 -0.167549406
H -1.785007121 -5.011983902 -1.564949894
H -1.742875676 -5.456801813 0.153017956
H -3.278774914 -5.331191083 -0.708674964
H -7.869519341 -0.594059404 -0.312065716
H -7.580064934 -0.822378307 1.414982989
H -7.827200754 0.796115131 0.772796222
H -2.13242103 5.422633305 0.646418495
H -1.864707462 5.354202992 -1.105522298
H -3.501710285 5.41558452 -0.458349123
H 1.760585572 5.396316439 -0.66422223
H 1.75443053 5.191581793 1.099428
H 3.269848272 5.395225174 0.243101175

28H (0.333 nN at 2-5, C_i)

N 3.93217979 1.499114108 -0.051615816
N 0.092751833 3.601727985 0.052236434
N -3.795938731 1.399638901 -0.037689303
N -3.93217979 -1.499114108 0.051615816

N -0.092751833 -3.601727985 -0.052236434
N 3.795938731 -1.399638901 0.037689303
C 5.870702141 -0.01429995 0.145565433
C 5.29308669 1.221230207 0.067340019
C 5.962264186 2.519300436 0.060158126
C 5.030596915 3.489241406 -0.057163342
C 3.703214328 2.877203559 -0.123884063
C 2.513806321 3.544732948 -0.197237171
C 1.224631709 2.888287793 -0.26297762
C 0.787813324 1.604093709 -0.635413299
C -0.618213235 1.572489905 -0.530979804
C -1.056683516 2.827746913 -0.091104858
C -2.339722381 3.425592777 0.134221913
C -3.560617416 2.789847314 0.097058614
C -4.846236595 3.482872933 0.155282632
C -5.811966641 2.541797573 0.036017977
C -5.131412909 1.250969967 -0.06660663
C -5.870702141 0.01429995 -0.145565433
C -5.29308669 -1.221230207 -0.067340019
C -5.962264186 -2.519300436 -0.060158126
C -5.030596915 -3.489241406 0.057163342
C -3.703214328 -2.877203559 0.123884063
C -2.513806321 -3.544732948 0.197237171
C -1.224631709 -2.888287793 0.26297762
C -0.787813324 -1.604093709 0.635413299
C 0.618213235 -1.572489905 0.530979804
C 1.056683516 -2.827746913 0.091104858
C 2.339722381 -3.425592777 -0.134221913
C 3.560617416 -2.789847314 -0.097058614
C 4.846236595 -3.482872933 -0.155282632
C 5.811966641 -2.541797573 -0.036017977
C 5.131412909 -1.250969967 0.06660663
H 3.254301736 0.746848975 -0.019177391
H 0.106240163 4.550154984 0.383824202
H -3.254301736 -0.746848975 0.019177391
H -0.106240163 -4.550154984 -0.383824202
H 7.027697019 2.664282604 0.126111008
H 5.219637292 4.548876668 -0.099066736
H 1.407871413 0.800120559 -1.000679052
H -1.270125107 0.751040616 -0.775346573
H -5.0051781 4.545077783 0.242171918
H -6.878737782 2.698174817 0.028243072
H -7.027697019 -2.664282604 -0.126111008
H -5.219637292 -4.548876668 0.099066736
H -1.407871413 -0.800120559 1.000679052
H 1.270125107 -0.751040616 0.775346573
H 5.0051781 -4.545077783 -0.242171918

H 6.878737782 -2.698174817 -0.028243072
C 7.371753886 -0.141655254 0.279486291
C 2.478576332 5.061250579 -0.157729747
C -2.289573759 4.914100876 0.420973319
C -7.371753886 0.141655254 -0.279486291
C -2.478576332 -5.061250579 0.157729747
C 2.289573759 -4.914100876 -0.420973319
H 7.633205004 -0.778580046 1.130908231
H 7.816734637 -0.591628546 -0.615567107
H 7.853287869 0.822163903 0.437152239
H 1.812223989 5.448675299 -0.936889168
H 2.109529363 5.428097676 0.810881412
H 3.455570649 5.526525164 -0.31221392
H -1.728042041 5.102782907 1.344513864
H -1.794658701 5.452707381 -0.394905416
H -3.273761648 5.348696804 0.559672581
H -7.816734637 0.591628546 0.615567107
H -7.633205004 0.778580046 -1.130908231
H -7.853287869 -0.822163903 -0.437152239
H -2.109529363 -5.428097676 -0.810881412
H -1.812223989 -5.448675299 0.936889168
H -3.455570649 -5.526525164 0.31221392
H 1.794658701 -5.452707381 0.394905416
H 1.728042041 -5.102782907 -1.344513864
H 3.273761648 -5.348696804 -0.559672581

28H (0.333 nN at 2-6, C_i)

N 3.913418865 1.460868052 -0.05240671
N 0.064212067 3.596055434 0.054158982
N -3.8157387 1.440629546 -0.044591966
N -3.913418865 -1.460868052 0.05240671
N -0.064212067 -3.596055434 -0.054158982
N 3.8157387 -1.440629546 0.044591966
C 5.872350413 -0.034043346 0.136077734
C 5.278386596 1.1944399 0.061353628
C 5.934978278 2.498671589 0.058606416
C 4.99435531 3.460614567 -0.04889027
C 3.67170409 2.837952031 -0.115479498
C 2.479986567 3.503023672 -0.182182216
C 1.185261016 2.861186795 -0.252088588
C 0.727982259 1.587042807 -0.632399515
C -0.679190385 1.58348804 -0.54352872
C -1.096945835 2.846661468 -0.104063885
C -2.369680914 3.466951859 0.113538131
C -3.591177505 2.833132002 0.078172424
C -4.880605772 3.51734704 0.133906431

C	-5.83979536	2.56718405	0.026434114
C	-5.149046344	1.280500687	-0.067497017
C	-5.872350413	0.034043346	-0.136077734
C	-5.278386596	-1.1944399	-0.061353628
C	-5.934978278	-2.498671589	-0.058606416
C	-4.99435531	-3.460614567	0.04889027
C	-3.67170409	-2.837952031	0.115479498
C	-2.479986567	-3.503023672	0.182182216
C	-1.185261016	-2.861186795	0.252088588
C	-0.727982259	-1.587042807	0.632399515
C	0.679190385	-1.58348804	0.54352872
C	1.096945835	-2.846661468	0.104063885
C	2.369680914	-3.466951859	-0.113538131
C	3.591177505	-2.833132002	-0.078172424
C	4.880605772	-3.51734704	-0.133906431
C	5.83979536	-2.56718405	-0.026434114
C	5.149046344	-1.280500687	0.067497017
H	3.243445585	0.701201304	-0.02929561
H	0.090627929	4.542877196	0.38944448
H	-3.243445585	-0.701201304	0.02929561
H	-0.090627929	-4.542877196	-0.38944448
H	6.999069754	2.653494907	0.122780419
H	5.174517793	4.521985209	-0.082963924
H	1.337013737	0.772677016	-0.992784898
H	-1.345402819	0.777328462	-0.798802538
H	-5.045209857	4.579661225	0.211622875
H	-6.907857033	2.715095357	0.021048435
H	-6.999069754	-2.653494907	-0.122780419
H	-5.174517793	-4.521985209	0.082963924
H	-1.337013737	-0.772677016	0.992784898
H	1.345402819	-0.777328462	0.798802538
H	5.045209857	-4.579661225	-0.211622875
H	6.907857033	-2.715095357	-0.021048435
C	7.375640125	-0.143921132	0.260356509
C	2.442938139	5.018597281	-0.136459451
C	-2.310580222	4.95447328	0.409206758
C	-7.375640125	0.143921132	-0.260356509
C	-2.442938139	-5.018597281	0.136459451
C	2.310580222	-4.95447328	-0.409206758
H	7.649809345	-0.770972345	1.115184156
H	7.817993007	-0.597572787	-0.63412873
H	7.849241307	0.825639572	0.405530545
H	1.76418903	5.407767978	-0.903692544
H	2.091866156	5.383284312	0.839516108
H	3.41463747	5.480444374	-0.309293181
H	-1.764304888	5.128832713	1.345008905
H	-1.800776801	5.511196465	-0.387463645

H -3.296191399 5.390446201 0.538415769
H -7.817993007 0.597572787 0.63412873
H -7.649809345 0.770972345 -1.115184156
H -7.849241307 -0.825639572 -0.405530545
H -2.091866156 -5.383284312 -0.839516108
H -1.76418903 -5.407767978 0.903692544
H -3.41463747 -5.480444374 0.309293181
H 1.800776801 -5.511196465 0.387463645
H 1.764304888 -5.128832713 -1.345008905
H 3.296191399 -5.390446201 -0.538415769

28H (0.333 nN at 3-5, C_i)

N 3.913418865 1.460868052 -0.05240671
N 0.064212067 3.596055434 0.054158982
N -3.8157387 1.440629546 -0.044591966
N -3.913418865 -1.460868052 0.05240671
N -0.064212067 -3.596055434 -0.054158982
N 3.8157387 -1.440629546 0.044591966
C 5.872350414 -0.034043346 0.136077734
C 5.278386596 1.1944399 0.061353628
C 5.934978278 2.498671589 0.058606416
C 4.99435531 3.460614567 -0.04889027
C 3.67170409 2.837952031 -0.115479498
C 2.479986567 3.503023672 -0.182182216
C 1.185261016 2.861186795 -0.252088588
C 0.727982259 1.587042807 -0.632399515
C -0.679190385 1.58348804 -0.54352872
C -1.096945835 2.846661468 -0.104063885
C -2.369680914 3.466951859 0.113538131
C -3.591177505 2.833132002 0.078172424
C -4.880605772 3.51734704 0.133906431
C -5.83979536 2.56718405 0.026434114
C -5.149046344 1.280500687 -0.067497017
C -5.872350414 0.034043346 -0.136077734
C -5.278386596 -1.1944399 -0.061353628
C -5.934978278 -2.498671589 -0.058606416
C -4.99435531 -3.460614567 0.04889027
C -3.67170409 -2.837952031 0.115479498
C -2.479986567 -3.503023672 0.182182216
C -1.185261016 -2.861186795 0.252088588
C -0.727982259 -1.587042807 0.632399515
C 0.679190385 -1.58348804 0.54352872
C 1.096945835 -2.846661468 0.104063885
C 2.369680914 -3.466951859 -0.113538131
C 3.591177505 -2.833132002 -0.078172424
C 4.880605772 -3.51734704 -0.133906431

C 5.83979536 -2.56718405 -0.026434114
C 5.149046344 -1.280500687 0.067497017
H 3.243445585 0.701201304 -0.02929561
H 0.090627929 4.542877196 0.38944448
H -3.243445585 -0.701201304 0.02929561
H -0.090627929 -4.542877196 -0.38944448
H 6.999069754 2.653494907 0.122780419
H 5.174517793 4.521985209 -0.082963924
H 1.337013737 0.772677016 -0.992784898
H -1.345402819 0.777328462 -0.798802538
H -5.045209857 4.579661225 0.211622875
H -6.907857033 2.715095357 0.021048435
H -6.999069754 -2.653494907 -0.122780419
H -5.174517793 -4.521985209 0.082963924
H -1.337013737 -0.772677016 0.992784898
H 1.345402819 -0.777328462 0.798802538
H 5.045209857 -4.579661225 -0.211622875
H 6.907857033 -2.715095357 -0.021048435
C 7.375640125 -0.143921132 0.260356509
C 2.442938139 5.01859728 -0.136459451
C -2.310580222 4.95447328 0.409206758
C -7.375640125 0.143921132 -0.260356509
C -2.442938139 -5.01859728 0.136459451
C 2.310580222 -4.95447328 -0.409206758
H 7.649809345 -0.770972345 1.115184156
H 7.817993008 -0.597572787 -0.63412873
H 7.849241307 0.825639572 0.405530545
H 1.76418903 5.407767978 -0.903692544
H 2.091866156 5.383284312 0.839516108
H 3.41463747 5.480444374 -0.309293181
H -1.764304888 5.128832713 1.345008905
H -1.800776801 5.511196465 -0.387463645
H -3.296191399 5.390446201 0.538415769
H -7.817993008 0.597572787 0.63412873
H -7.649809345 0.770972345 -1.115184156
H -7.849241307 -0.825639572 -0.405530545
H -2.091866156 -5.383284312 -0.839516108
H -1.76418903 -5.407767978 0.903692544
H -3.41463747 -5.480444374 0.309293181
H 1.800776801 -5.511196465 0.387463645
H 1.764304888 -5.128832713 -1.345008905
H 3.296191399 -5.390446201 -0.538415769

28H (0.333 nN at 3-6, C_i)

N 3.890593898 1.405224995 -0.042507658
N 0.012185396 3.603409274 0.079200013

N	-3.857085294	1.499023361	-0.032268662
N	-3.890593898	-1.405224995	0.042507658
N	-0.012185396	-3.603409274	-0.079200013
N	3.857085294	-1.499023361	0.032268662
C	5.885459653	-0.061172846	0.068893825
C	5.263565317	1.155818947	0.029913494
C	5.900304722	2.468309893	0.048593669
C	4.944215871	3.418343897	0.000317157
C	3.626799246	2.781101321	-0.05614646
C	2.43155196	3.446452238	-0.087876375
C	1.124334866	2.835381044	-0.188615112
C	0.643576637	1.57525738	-0.583150049
C	-0.765267986	1.610751324	-0.537906853
C	-1.162582703	2.886159581	-0.115026554
C	-2.4316998	3.534622835	0.053358983
C	-3.649298858	2.896957539	0.041602429
C	-4.945231481	3.567759667	0.094841327
C	-5.893759817	2.602164959	0.039879692
C	-5.186663886	1.321326672	-0.029519265
C	-5.885459653	0.061172846	-0.068893825
C	-5.263565317	-1.155818947	-0.029913494
C	-5.900304722	-2.468309893	-0.048593669
C	-4.944215871	-3.418343897	-0.000317157
C	-3.626799246	-2.781101321	0.05614646
C	-2.43155196	-3.446452238	0.087876375
C	-1.124334866	-2.835381044	0.188615112
C	-0.643576637	-1.57525738	0.583150049
C	0.765267986	-1.610751324	0.537906853
C	1.162582703	-2.886159581	0.115026554
C	2.4316998	-3.534622835	-0.053358983
C	3.649298858	-2.896957539	-0.041602429
C	4.945231481	-3.567759667	-0.094841327
C	5.893759817	-2.602164959	-0.039879692
C	5.186663886	-1.321326672	0.029519265
H	3.234233637	0.63354664	-0.032889206
H	0.051350141	4.548190845	0.419160864
H	-3.234233637	-0.63354664	0.032889206
H	-0.051350141	-4.548190845	-0.419160864
H	6.962973577	2.638379707	0.091050326
H	5.115088845	4.48127026	-0.002964505
H	1.240260151	0.744333794	-0.924988553
H	-1.445474323	0.824920864	-0.817381619
H	-5.118336843	4.630366412	0.148517357
H	-6.963688685	2.73576675	0.053240231
H	-6.962973577	-2.638379707	-0.091050326
H	-5.115088845	-4.48127026	0.002964505
H	-1.240260151	-0.744333794	0.924988553

H 1.445474323 -0.824920864 0.817381619<br>H 5.118336843 -4.630366412 -0.148517357<br>H 6.963688685 -2.73576675 -0.053240231<br>C 7.393961313 -0.14361597 0.141337815<br>C 2.411097061 4.959269786 0.007074293<br>C -2.366283532 5.038138797 0.254476069<br>C -7.393961313 0.14361597 -0.141337815<br>C -2.411097061 -4.959269786 -0.007074293<br>C 2.366283532 -5.038138797 -0.254476069<br>H 7.710735486 -0.738941751 1.004369766<br>H 7.807771083 -0.620077311 -0.754782994<br>H 7.859044511 0.836020739 0.234897505<br>H 1.774433947 5.387344566 -0.775520041<br>H 2.024857695 5.295773699 0.979561926<br>H 3.39753229 5.40099724 -0.105724518<br>H -1.884754798 5.271824442 1.213055776<br>H -1.775584283 5.50483382 -0.541535036<br>H -3.343239944 5.523318113 0.266597711<br>H -7.807771083 0.620077311 0.754782994<br>H -7.710735486 0.738941751 -1.004369766<br>H -7.859044511 -0.836020739 -0.234897505<br>H -2.024857695 -5.295773699 -0.979561926<br>H -1.774433947 -5.387344566 0.775520041<br>H -3.39753229 -5.40099724 0.105724518<br>H 1.775584283 -5.50483382 0.541535036<br>H 1.884754798 -5.271824442 -1.213055776<br>H 3.343239944 -5.523318113 -0.266597711
**28H** (0.333 nN at 5-6, C_1)<br><br>N 3.918775444 -1.388171139 -0.230154436<br>N 0.044527552 -3.529599521 -0.165413904<br>N -3.78740331 -1.403680886 0.298372358<br>N -3.856442563 1.416957804 -0.232246246<br>N -0.114857817 3.517426643 0.069385995<br>N 3.784246035 1.403252166 0.211665358<br>C 5.871748168 0.1029152 -0.124262794<br>C 5.280194819 -1.110855909 -0.337264341<br>C 5.918105653 -2.38447899 -0.65081104<br>C 4.96789769 -3.343840448 -0.704084364<br>C 3.660150088 -2.745600493 -0.426624574<br>C 2.462962785 -3.404675549 -0.367327123<br>C 1.198780359 -2.790483915 -0.032615243<br>C 0.799596682 -1.532631672 0.454897011<br>C -0.603676006 -1.533159944 0.581532317<br>C -1.077862707 -2.793378539 0.195452285<br>C -2.358486257 -3.434929046 0.236674649

C -3.569190864 -2.798474041 0.371393502
C -4.850160513 -3.469601591 0.578706032
C -5.799087644 -2.503890268 0.631678102
C -5.110820279 -1.227476441 0.429602565
C -5.823930535 0.018620973 0.304554437
C -5.220417873 1.194664568 -0.041967893
C -5.873633887 2.47086706 -0.326157378
C -4.934083809 3.373148626 -0.67547306
C -3.611008426 2.746720067 -0.596479781
C -2.430377749 3.409056635 -0.752339962
C -1.121021975 2.805558153 -0.54683467
C -0.565439049 1.582921672 -0.940110869
C 0.79643466 1.586327641 -0.574123353
C 1.076973399 2.788926589 0.084348238
C 2.266875037 3.318725987 0.686971949
C 3.498983812 2.702830723 0.682981849
C 4.754004011 3.356793889 1.049796857
C 5.754918612 2.491743425 0.751603686
C 5.12095584 1.274749092 0.247926605
H 3.267652246 -0.629430897 -0.066433681
H 0.016467594 -4.469104452 -0.520251611
H -3.189299981 0.662459715 -0.111080227
H -0.243659517 4.427324132 0.479930142
H 6.976207683 -2.523901999 -0.800217214
H 5.137798829 -4.388671306 -0.903028956
H 1.4459106 -0.719813274 0.742122124
H -1.224413306 -0.730006697 0.942888398
H -5.011742262 -4.529028858 0.695810457
H -6.859444788 -2.63615784 0.777487228
H -6.935960473 2.642989237 -0.274212687
H -5.103796057 4.404368745 -0.937999758
H -1.083090019 0.804389443 -1.479047788
H 1.529259126 0.827846532 -0.780853817
H 4.870622952 4.358680139 1.43037386
H 6.815480046 2.64982819 0.869320409
C 7.371558118 0.256025966 -0.215877108
C 2.399424114 -4.88530643 -0.673694741
C -2.316028448 -4.944460028 0.11650619
C -7.320236513 -0.042335824 0.511706215
C -2.453596557 4.882094445 -1.125458621
C 2.136583929 4.676627567 1.346370323
H 7.638526575 1.106555733 -0.851412847
H 7.816234006 0.431988487 0.770970425
H 7.847488782 -0.629009147 -0.637342327
H 1.92337905 -5.435596368 0.147208459
H 1.824502365 -5.079719311 -1.589429012
H 3.383904404 -5.32162494 -0.823406997

H -1.986733548 -5.237054674 -0.889343281<br>H -1.61685297 -5.374999088 0.840744315<br>H -3.288989205 -5.40136046 0.270713954<br>H -7.807897038 -0.651943714 -0.257963263<br>H -7.55798925 -0.489877793 1.482613755<br>H -7.779493816 0.94490617 0.492731327<br>H -2.521627625 5.541783545 -0.249458668<br>H -1.544778143 5.145556701 -1.673341978<br>H -3.305547886 5.112571001 -1.776366775<br>H 1.956966645 5.466861745 0.607414285<br>H 1.295759013 4.674839044 2.04926274<br>H 3.029259714 4.934419023 1.918833318
**28M$_{1a}$** (0.333 nN at 1-4, C$_1$)<br><br>N -3.398777502 1.58726447 0.555160194<br>N 0.578952546 3.330959335 -0.505866857<br>N 3.923852301 0.661135442 -0.357665784<br>N 3.143343892 -1.455085697 0.925089953<br>N -1.000516202 -2.01073658 -0.011726082<br>N -3.685406424 -0.953546344 -0.320770754<br>C -5.45649733 0.251388454 0.887198501<br>C -4.649654717 1.394221562 1.131817774<br>C -4.96734189 2.593748192 1.803905024<br>C -3.920608804 3.490035919 1.601364524<br>C -2.933211207 2.85070804 0.806826887<br>C -1.697581091 3.420441551 0.338582506<br>C -0.613274598 2.708421739 -0.116420765<br>C -0.340993094 1.281285457 -0.233570751<br>C 0.95633668 1.105708056 -0.607711988<br>C 1.593083805 2.402302746 -0.766331286<br>C 2.881785396 2.758637796 -1.08857636<br>C 3.99662195 1.850863736 -0.971143503<br>C 5.387200509 2.142818591 -1.316241958<br>C 6.140358237 1.097337748 -0.862232247<br>C 5.226722117 0.160596928 -0.231400581<br>C 5.51669852 -0.9649778 0.530042669<br>C 4.480597547 -1.716969683 1.160347965<br>C 4.516114493 -2.735741077 2.13922191<br>C 3.194400316 -3.041218775 2.491717519<br>C 2.340803728 -2.240460497 1.698261837<br>C 0.896573024 -2.201570874 1.56058571<br>C 0.348517308 -2.178073682 0.304916761<br>C 0.996111435 -2.518326047 -0.962916203<br>C 0.050252562 -2.619905217 -1.928149595<br>C -1.254733429 -2.30513791 -1.356346444<br>C -2.492149433 -2.367644193 -1.939957622

C -3.678290262 -1.936626754 -1.242237569<br>C -5.016554452 -2.472499345 -1.426494688<br>C -5.832385172 -1.806582183 -0.553653156<br>C -5.013163604 -0.812599907 0.112629247<br>H -3.002823322 0.84170556 -0.017148546<br>H 0.748973918 4.316178351 -0.403747086<br>H 2.906377848 -0.615479307 0.376817061<br>H -1.737994483 -1.70328798 0.606314407<br>H -5.870463498 2.778173262 2.363739398<br>H -3.853050668 4.490772424 1.994680389<br>H -1.050217155 0.485863826 -0.066403925<br>H 1.443045793 0.169955574 -0.82259706<br>H 5.748642083 3.022919218 -1.824501539<br>H 7.21159879 0.990380603 -0.941909646<br>H 5.408765792 -3.189785337 2.541134582<br>H 2.87127269 -3.792904326 3.194190261<br>H 2.05309266 -2.710597605 -1.060028998<br>H 0.216617152 -2.893187596 -2.957566083<br>H -5.289594826 -3.26533801 -2.105834766<br>H -6.894797179 -1.946271547 -0.421143618<br>C -6.874052523 0.266905523 1.415737908<br>C -1.569066548 4.927657057 0.395244437<br>C 3.170539768 4.207979342 -1.403816033<br>C 6.936911769 -1.398879828 0.804150032<br>C 0.03912234 -2.193826019 2.797299097<br>C -2.678644397 -2.925384317 -3.329579276<br>H -6.892154399 0.667939543 2.432862271<br>H -7.553681127 0.883134992 0.810170869<br>H -7.281389052 -0.743886389 1.453756069<br>H -0.899459841 5.253662776 1.201814024<br>H -1.171206304 5.316070055 -0.550132758<br>H -2.536547703 5.404856361 0.54645158<br>H 3.4003212 4.787294138 -0.499456053<br>H 4.027931106 4.302674713 -2.072131624<br>H 2.31921391 4.679143702 -1.905364871<br>H 7.040232227 -2.476659235 0.645781742<br>H 7.678409741 -0.894752304 0.178323353<br>H 7.194706368 -1.198848416 1.850572265<br>H -0.164244159 -1.170952772 3.13919095<br>H -0.920806216 -2.690319545 2.623706375<br>H 0.547287568 -2.708659167 3.617459515<br>H -3.258352599 -3.855958317 -3.301998793<br>H -3.221395555 -2.222414584 -3.969365999<br>H -1.726250268 -3.153079023 -3.80919002
**28M$_{1a}$** (0.333 nN at 1-5, C_1)

N -3.511166393 1.720175052 0.510091956
N 0.50254034 3.339044342 -0.455394914
N 3.885385332 0.649634123 -0.284517907
N 3.239242562 -1.558703709 1.003733281
N -0.91029909 -2.149504152 0.219323369
N -3.727539103 -0.857006737 -0.235884642
C -5.596106459 0.413842517 0.738295016
C -4.8104277 1.57120688 0.976215894
C -5.179004127 2.811793677 1.539117982
C -4.107208658 3.687271574 1.38491738
C -3.059023225 2.993616448 0.724875698
C -1.786765666 3.51627735 0.311988001
C -0.699905355 2.755746655 -0.048012392
C -0.434813699 1.320933985 -0.046834487
C 0.866959034 1.111578527 -0.388905637
C 1.510178539 2.389428708 -0.645167442
C 2.790046026 2.73159344 -1.011901444
C 3.914405057 1.836627847 -0.911854298
C 5.280152594 2.143136817 -1.333215294
C 6.068590198 1.111803624 -0.914246723
C 5.20081335 0.167885401 -0.232730257
C 5.560535814 -0.969203927 0.477761236
C 4.596402663 -1.782696829 1.132811564
C 4.737252935 -2.881814566 2.008632582
C 3.455731958 -3.284654 2.401259182
C 2.520819018 -2.457390031 1.73619461
C 1.074986024 -2.497524271 1.68566195
C 0.455265858 -2.309380343 0.474277994
C 1.070346061 -2.463014546 -0.846086226
C 0.09806565 -2.488170204 -1.786804296
C -1.197712087 -2.285720893 -1.149356422
C -2.430615784 -2.313405367 -1.737747135
C -3.648071249 -1.914472969 -1.061763251
C -4.958169817 -2.515306793 -1.253150532
C -5.839084261 -1.792817025 -0.497867757
C -5.084885088 -0.713862518 0.109762891
H -3.07424091 0.93572643 0.023449232
H 0.668174815 4.329923215 -0.457425371
H 2.928221007 -0.706416025 0.521561234
H -1.622183412 -1.943072879 0.902447279
H -6.128730589 3.034464795 1.999032671
H -4.065647825 4.709330095 1.722929505
H -1.14898068 0.545459972 0.186577934
H 1.355229193 0.160805213 -0.513554746
H 5.604737502 3.019696272 -1.870704197
H 7.134432595 1.018022169 -1.055133847
H 5.672279879 -3.327482615 2.310849113

H 3.210659377 -4.115994884 3.042701679<br>H 2.128458595 -2.603136565 -0.999583921<br>H 0.23893933 -2.626725466 -2.846445431<br>H -5.17199456 -3.385410126 -1.85448329<br>H -6.901279903 -1.960404687 -0.404259489<br>C -7.064611938 0.502891278 1.086982688<br>C -1.627458131 5.020457311 0.273077888<br>C 3.043746289 4.171973401 -1.39615253<br>C 7.004451984 -1.368217394 0.634956585<br>C 0.342561731 -2.810012536 2.963125363<br>C -2.585202945 -2.722388131 -3.18451199<br>H -7.190258365 0.923588234 2.08885429<br>H -7.615019159 1.151316403 0.388801801<br>H -7.532993996 -0.48089249 1.081196336<br>H -0.914877991 5.375353442 1.02874874<br>H -1.263324725 5.345300497 -0.710068634<br>H -2.575546104 5.529639989 0.438560551<br>H 3.156124824 4.81519658 -0.512388045<br>H 3.953741873 4.276925291 -1.985750469<br>H 2.220139084 4.567413154 -2.000586174<br>H 7.138068959 -2.428937174 0.402740649<br>H 7.659769594 -0.790860427 -0.015477912<br>H 7.332485666 -1.214866257 1.669092202<br>H 0.145386583 -1.910065004 3.558253197<br>H -0.611831072 -3.309290266 2.770548172<br>H 0.963861845 -3.47264652 3.582325286<br>H -3.396932604 -3.4494154 -3.289554756<br>H -2.841439156 -1.864944398 -3.817055255<br>H -1.681779854 -3.186283992 -3.581421188
**28M$_{1a}$** (0.333 nN at 2-5, C$_1$)<br><br>N -3.478358056 1.790012307 0.47159652<br>N 0.450553663 3.357752856 -0.691581569<br>N 3.858545227 0.621240777 -0.171282236<br>N 3.217347708 -1.696036041 0.986080995<br>N -0.905848685 -2.201929109 0.084346162<br>N -3.644921147 -0.795384069 -0.18854523<br>C -5.428012072 0.379753563 1.016109942<br>C -4.703655685 1.597773439 1.094221891<br>C -5.096678262 2.858715053 1.596342<br>C -4.114383074 3.785671139 1.247546638<br>C -3.093239482 3.098666566 0.544045301<br>C -1.86769188 3.624888288 0.004313277<br>C -0.748695332 2.850925259 -0.18679348<br>C -0.452185948 1.459014099 0.147399709<br>C 0.859187525 1.2127455 -0.107989038

C 1.483945961 2.410726266 -0.644862313
C 2.771344369 2.681416767 -1.037660038
C 3.900328603 1.817806568 -0.781242323
C 5.287688629 2.195714544 -1.044055007
C 6.075155604 1.198746887 -0.549311836
C 5.185752475 0.199930437 0.013994034
C 5.536516925 -0.952084728 0.705581887
C 4.568508775 -1.851475155 1.226352383
C 4.697094355 -2.979964963 2.065721702
C 3.41275781 -3.467780749 2.328716058
C 2.489436116 -2.661714609 1.621616869
C 1.054669088 -2.747693503 1.500566922
C 0.45572371 -2.385193259 0.316434661
C 1.073801802 -2.350613277 -1.012381466
C 0.098633156 -2.275024444 -1.949244088
C -1.197195221 -2.169033916 -1.288920933
C -2.442844416 -2.138427867 -1.842003459
C -3.618227026 -1.827332599 -1.046706102
C -4.90913788 -2.488711552 -1.093339024
C -5.719714321 -1.834445877 -0.203261738
C -4.947717869 -0.730098638 0.332000622
H -3.05203021 1.004729816 -0.026994571
H 0.593520749 4.327454205 -0.911238757
H 2.906706237 -0.853278153 0.492684165
H -1.612703598 -2.05911204 0.787544231
H -6.004294448 3.060433345 2.143290899
H -4.102127754 4.835904826 1.490791735
H -1.153994016 0.74422319 0.547592526
H 1.376519513 0.282809433 0.034419498
H 5.624131273 3.105865255 -1.514103943
H 7.153480176 1.163354077 -0.565193894
H 5.626802259 -3.383917793 2.435293275
H 3.161671896 -4.331322031 2.923199611
H 2.13293522 -2.45805724 -1.18435945
H 0.236119495 -2.28229025 -3.018753583
H -5.154030895 -3.35099037 -1.694740964
H -6.752315963 -2.058301826 0.018608703
C -6.824910667 0.376439963 1.583655562
C -1.794358004 5.103893083 -0.316173098
C 3.047155937 4.023738675 -1.680318705
C 6.979798345 -1.282694979 0.987027273
C 0.285551028 -3.308865882 2.669853691
C -2.664000038 -2.369802963 -3.315536809
H -6.834084756 0.835398341 2.57611437
H -7.508142887 0.949975507 0.948019155
H -7.211317048 -0.637845208 1.679945897
H -1.149144192 5.642122789 0.390545665

H -1.382774362 5.256709702 -1.322234371
H -2.77628339 5.587589325 -0.308934321
H 3.277252035 4.800056162 -0.93879405
H 3.892425611 3.965200162 -2.366869281
H 2.189782736 4.360366441 -2.271569056
H 7.193665996 -2.323274699 0.726234044
H 7.662386148 -0.644061559 0.429046436
H 7.19736486 -1.160161221 2.053951454
H 0.277757697 -2.63014779 3.529645591
H -0.74847019 -3.532475113 2.395146898
H 0.744969688 -4.252730594 3.005156781
H -3.476372935 -3.089242885 -3.467549701
H -2.958368764 -1.445423692 -3.825527477
H -1.774699867 -2.767855381 -3.806948447

28M$_{1a}$ (0.333 nN at 2-6, C_1)

N -3.287787027 1.561638466 0.569830321
N 0.642174922 3.407119524 -0.464709943
N 3.908977138 0.715193974 -0.403070186
N 3.055938608 -1.375523117 0.8915582
N -1.05707381 -1.997441919 -0.127238514
N -3.650914295 -1.032449014 -0.422967057
C -5.352409812 0.200538954 0.852635767
C -4.518882725 1.310502896 1.165408972
C -4.821019234 2.441537132 1.949473894
C -3.785056709 3.362313284 1.796671597
C -2.822596181 2.807465451 0.921132024
C -1.610782038 3.442152653 0.467120682
C -0.536066843 2.769264781 -0.05772201
C -0.272740866 1.346943966 -0.246797253
C 1.006510172 1.185087851 -0.67749853
C 1.642536492 2.486584243 -0.79624319
C 2.929842562 2.83701094 -1.123473102
C 4.020205727 1.899445101 -1.019311517
C 5.419852289 2.150942319 -1.357978066
C 6.139984239 1.086679983 -0.894298145
C 5.194259059 0.179779042 -0.262972285
C 5.441451711 -0.936686229 0.523487897
C 4.382783343 -1.650596686 1.159003303
C 4.386993469 -2.634144546 2.172382873
C 3.053874205 -2.904032886 2.515496058
C 2.229323837 -2.118363989 1.680663496
C 0.789215845 -2.060221179 1.50280652
C 0.279388634 -2.152457734 0.235445788
C 0.946575298 -2.64408095 -0.971988978
C 0.014001587 -2.849999827 -1.935368487

C -1.293159054 -2.448600418 -1.426506548
C -2.532389566 -2.552226759 -2.008697499
C -3.692471561 -2.016709871 -1.348117791
C -5.059471217 -2.474717419 -1.543042106
C -5.840076542 -1.773658214 -0.669455521
C -4.967984467 -0.832867169 0.014663611
H -2.905248456 0.890801064 -0.089849595
H 0.824213777 4.383946782 -0.313169984
H 2.838532613 -0.547939184 0.320761809
H -1.809702008 -1.607450135 0.426288306
H -5.707384243 2.567029039 2.550499774
H -3.706758259 4.325382659 2.273809321
H -0.97348388 0.544532763 -0.081445804
H 1.480787449 0.258674554 -0.953234022
H 5.807137165 3.019173626 -1.868067532
H 7.208370667 0.950779036 -0.967471733
H 5.265932826 -3.088255654 2.603370466
H 2.705544019 -3.624564695 3.238259225
H 2.002004322 -2.861317399 -1.023827612
H 0.189223493 -3.251315934 -2.920603863
H -5.369871787 -3.246657083 -2.230781527
H -6.908941382 -1.857850943 -0.545448874
C -6.756740362 0.24875441 1.403699505
C -1.531256114 4.951325147 0.603749523
C 3.251134351 4.28673715 -1.396909234
C 6.846153675 -1.393340403 0.81388082
C -0.101182272 -1.904973884 2.705427436
C -2.724466356 -3.286389025 -3.317571664
H -6.732863693 0.428089273 2.48311374
H -7.332606628 1.061432021 0.948850881
H -7.283580157 -0.689678702 1.238076895
H -0.952895752 5.290633542 1.477959159
H -1.071522578 5.391681004 -0.28887662
H -2.529739684 5.383388673 0.68281015
H 3.542615662 4.82291078 -0.483953516
H 4.080646118 4.37805427 -2.101199306
H 2.394627224 4.803976129 -1.840572061
H 6.940321273 -2.472379047 0.660030438
H 7.575120071 -0.891009007 0.178878743
H 7.106898937 -1.187006289 1.858048548
H -0.287744907 -0.846998067 2.931465253
H -1.06874415 -2.394064339 2.555103904
H 0.372554128 -2.341418615 3.589176741
H -3.088218912 -4.317445589 -3.177569283
H -3.449398609 -2.767813333 -3.951576163
H -1.788416718 -3.351888642 -3.875721837

28M$_{1a}$ (0.333 nN at 3-5, C_1)

N -3.347339091 1.599191191 0.549688365
N 0.542146389 3.376129475 -0.681147412
N 3.846372961 0.711461957 -0.333708007
N 3.047498017 -1.435445259 1.042068363
N -1.020108283 -2.050912773 0.021634408
N -3.635877206 -0.946921314 -0.306843534
C -5.362147824 0.226983289 0.972153483
C -4.561439321 1.377516968 1.188709725
C -4.875394655 2.572597463 1.872187716
C -3.864976096 3.494621863 1.612124832
C -2.903594887 2.875047607 0.770884242
C -1.704096397 3.461163802 0.239371024
C -0.628668076 2.750495621 -0.237085365
C -0.339630472 1.322966671 -0.308621217
C 0.951370088 1.154367331 -0.703160646
C 1.56889922 2.454625968 -0.904933984
C 2.86789843 2.799830771 -1.193129799
C 3.952003573 1.861183983 -1.02266596
C 5.344168158 2.085109797 -1.405674434
C 6.071043725 1.049264998 -0.89488084
C 5.13583112 0.183879351 -0.194274888
C 5.414548804 -0.909513929 0.614908235
C 4.393275334 -1.653356735 1.269966428
C 4.470261238 -2.688376142 2.228884516
C 3.164647223 -3.060995452 2.569132782
C 2.277780033 -2.285592952 1.788201015
C 0.842141705 -2.339455927 1.63259816
C 0.319219994 -2.208792089 0.366899868
C 1.010608506 -2.44298382 -0.903559679
C 0.092275942 -2.514180341 -1.899206875
C -1.231617879 -2.263418128 -1.345977357
C -2.455154362 -2.307183338 -1.96064363
C -3.645154478 -1.908069146 -1.251562892
C -4.983907647 -2.444959525 -1.416416027
C -5.780605492 -1.804685495 -0.505075596
C -4.948088346 -0.824007349 0.164374424
H -2.963317872 0.863511033 -0.042247658
H 0.680269293 4.371478659 -0.671387852
H 2.763596764 -0.615768325 0.497572759
H -1.774239202 -1.757485822 0.625736476
H -5.753623232 2.735599521 2.476654029
H -3.802199953 4.499107372 1.996738583
H -1.027962581 0.522503382 -0.085155284
H 1.455217229 0.221571925 -0.887188254
H 5.718119925 2.908423497 -1.994300744

H 7.135867324 0.901565807 -0.990474717<br>H 5.382037028 -3.113627869 2.618802615<br>H 2.873406956 -3.84234837 3.252610245<br>H 2.072834292 -2.611062684 -0.985087775<br>H 0.290860188 -2.726116086 -2.937572451<br>H -5.272450295 -3.223344243 -2.105981311<br>H -6.83853486 -1.954880768 -0.350555571<br>C -6.756471318 0.244408322 1.545935248<br>C -1.595568968 4.970024921 0.260726215<br>C 3.194392757 4.24753886 -1.50167484<br>C 6.83480596 -1.336098761 0.882720347<br>C -0.014690829 -2.61511873 2.843061456<br>C -2.609910909 -2.777050118 -3.384382544<br>H -6.729643108 0.530484517 2.60164144<br>H -7.389800445 0.969981612 1.0245294<br>H -7.224283629 -0.737435365 1.47985315<br>H -0.857789412 5.317293802 0.995759404<br>H -1.29348831 5.34975726 -0.723219509<br>H -2.551917693 5.435874047 0.49522039<br>H 2.773977066 4.915226491 -0.737055386<br>H 4.270973927 4.417013275 -1.490641746<br>H 2.816384165 4.586517823 -2.477601707<br>H 6.954957382 -2.409612058 0.708920046<br>H 7.545197883 -0.805163555 0.250892381<br>H 7.10101767 -1.141039124 1.927526648<br>H 0.060994556 -1.816638054 3.588173275<br>H -1.065557453 -2.72880468 2.566363319<br>H 0.286878693 -3.552360148 3.338141371<br>H -3.323049099 -3.607356288 -3.440954815<br>H -2.99093213 -1.979005696 -4.030501203<br>H -1.666489648 -3.132185189 -3.801080191
**28M$_{1a}$** (0.333 nN at 3-6, C_1)<br><br>N -3.286252202 1.515072086 0.499083469<br>N 0.686637354 3.480471805 -0.452115643<br>N 3.987991004 0.824928342 -0.314131665<br>N 3.067380443 -1.339916178 0.816041966<br>N -1.114734328 -2.008348151 -0.192930375<br>N -3.719782652 -1.108249768 -0.411991708<br>C -5.363195491 0.176873302 0.87788468<br>C -4.509335409 1.288617679 1.125905267<br>C -4.798412178 2.446242712 1.871630811<br>C -3.767720154 3.360922898 1.665398419<br>C -2.819724687 2.775382388 0.794450659<br>C -1.614481589 3.417066729 0.327954447<br>C -0.495052798 2.786897289 -0.149258564

C -0.173297618 1.384168049 -0.37923398<br>C 1.135054883 1.282634597 -0.732996087<br>C 1.741498839 2.609244356 -0.745522785<br>C 3.044585268 2.992353888 -0.954561827<br>C 4.127480583 2.043246924 -0.847894345<br>C 5.538969767 2.308610532 -1.119817561<br>C 6.237431182 1.208866516 -0.708687064<br>C 5.265024237 0.267649974 -0.173693749<br>C 5.47284784 -0.909311176 0.527492371<br>C 4.387351147 -1.653667649 1.082853162<br>C 4.363394558 -2.703813285 2.023454379<br>C 3.02013642 -2.975577777 2.330164398<br>C 2.220894783 -2.1198039 1.545773086<br>C 0.774589293 -2.017936434 1.383752885<br>C 0.232665611 -2.155957441 0.137811877<br>C 0.860106604 -2.709822247 -1.065033609<br>C -0.10476956 -2.973069659 -1.980719738<br>C -1.398043427 -2.547738033 -1.44873212<br>C -2.660059531 -2.6981313 -1.967426431<br>C -3.795812535 -2.130656811 -1.289503084<br>C -5.170491458 -2.59368235 -1.413972573<br>C -5.917610706 -1.852836906 -0.545011258<br>C -5.019068222 -0.887527639 0.066087422<br>H -2.91057783 0.81648422 -0.135542651<br>H 0.825981726 4.449159984 -0.224610732<br>H 2.869155511 -0.47372861 0.301068796<br>H -1.850984846 -1.596693949 0.366991112<br>H -5.674718572 2.593884028 2.482100522<br>H -3.683556097 4.340155677 2.106412122<br>H -0.864917519 0.558298566 -0.30938442<br>H 1.653344441 0.385871981 -1.02519595<br>H 5.942320286 3.210544787 -1.554090515<br>H 7.306825024 1.069278464 -0.756150615<br>H 5.229554427 -3.199631952 2.433841856<br>H 2.649221142 -3.736939447 2.997658306<br>H 1.914935636 -2.924980319 -1.140395772<br>H 0.037753604 -3.424653141 -2.94947935<br>H -5.511150396 -3.390699266 -2.056662306<br>H -6.981463807 -1.928233243 -0.379909782<br>C -6.746695228 0.258479235 1.47604004<br>C -1.563951132 4.924761069 0.468835487<br>C 3.415040029 4.443264693 -1.173225039<br>C 6.861851308 -1.413170454 0.814650673<br>C -0.081487956 -1.780138025 2.598681893<br>C -2.919464782 -3.506601847 -3.217613984<br>H -6.682189012 0.451886137 2.551388107<br>H -7.32312146 1.075176856 1.029515566

H -7.295602305 -0.672092727 1.341968468
H -1.020623684 5.23946881 1.369709487
H -1.071694548 5.376160733 -0.399581923
H -2.566812047 5.349009314 0.516103505
H 3.986167336 4.879848603 -0.339670415
H 4.042356516 4.536499615 -2.066744661
H 2.533004138 5.064227632 -1.349042986
H 6.943341621 -2.47675324 0.571927061
H 7.61570322 -0.871417403 0.244557386
H 7.095855429 -1.301067892 1.879260606
H -0.207196593 -0.707627002 2.796364566
H -1.076457336 -2.221437283 2.482987564
H 0.388204404 -2.215627594 3.485169378
H -3.55991382 -4.379352187 -3.012984102
H -3.42820246 -2.907864386 -3.980758535
H -1.991971579 -3.883985206 -3.650959217

28M$_{1a}$ (0.333 nN at 5-6, C_1)

N -3.333772167 1.54534809 0.596971883
N 0.576797333 3.324536815 -0.551662374
N 3.875034001 0.660186777 -0.398150728
N 3.065575333 -1.412055517 0.965289402
N -1.036361459 -2.006126987 -0.033162598
N -3.644866291 -0.971104745 -0.367652226
C -5.380110534 0.191453728 0.913550842
C -4.559890058 1.312675139 1.20738653
C -4.86057263 2.472676877 1.952195314
C -3.82855526 3.38853197 1.756827725
C -2.869751091 2.79950812 0.893452839
C -1.65980055 3.395517335 0.396245906
C -0.597627982 2.695713987 -0.122106457
C -0.331027156 1.269940116 -0.265782587
C 0.952066373 1.099195225 -0.685542714
C 1.581979479 2.398014462 -0.847779991
C 2.867136699 2.747337798 -1.185854041
C 3.967544535 1.826182361 -1.054105903
C 5.360554785 2.083048303 -1.412346111
C 6.097139639 1.042434567 -0.921546421
C 5.167793696 0.145271354 -0.254603779
C 5.439259651 -0.949913727 0.555394536
C 4.400150281 -1.667055486 1.214418774
C 4.432798243 -2.651014645 2.228355886
C 3.110142777 -2.944058042 2.586094122
C 2.260604307 -2.172706036 1.760145497
C 0.819103036 -2.146420748 1.605745911
C 0.298843966 -2.172649107 0.335135647

C 0.98621982 -2.548141237 -0.902670027
C 0.076306947 -2.648852532 -1.902903482
C -1.24302402 -2.305099065 -1.386950735
C -2.460334903 -2.340488884 -2.02198949
C -3.651123115 -1.901240085 -1.343706901
C -5.000287823 -2.390619291 -1.570947316
C -5.807021564 -1.759977096 -0.663762283
C -4.968540366 -0.828257018 0.067908532
H -2.955200145 0.838962112 -0.031501421
H 0.750306183 4.30875158 -0.444010457
H 2.826237085 -0.594886541 0.388140871
H -1.796892315 -1.677936406 0.547040675
H -5.743847821 2.618948299 2.553535742
H -3.752303878 4.368997842 2.197066642
H -1.03080069 0.471046349 -0.079270464
H 1.433599259 0.166966304 -0.927012613
H 5.733519191 2.937843088 -1.954614341
H 7.166278878 0.916389377 -1.000541968
H 5.324540846 -3.091337865 2.647145597
H 2.782292728 -3.671709469 3.311337603
H 2.042476873 -2.759174912 -0.958232773
H 0.277030728 -2.941784794 -2.920839815
H -5.28518628 -3.133487886 -2.300413386
H -6.87346703 -1.882440259 -0.548724042
C -6.786727978 0.214520861 1.456209767
C -1.534070979 4.900092903 0.491566064
C 3.17134155 4.191038803 -1.507886212
C 6.853997489 -1.380961944 0.837649121
C -0.047039939 -2.081745107 2.83864445
C -2.613430138 -2.912377962 -3.414169627
H -6.776106513 0.432913739 2.52845452
H -7.385865047 0.991082937 0.969047177
H -7.281323532 -0.746434774 1.31872365
H -0.812776656 5.204160545 1.261186994
H -1.201378035 5.320831152 -0.465354777
H -2.491360861 5.36583212 0.723173952
H 3.437161155 4.764920829 -0.610019124
H 4.012055813 4.270636485 -2.199719592
H 2.314749196 4.677513601 -1.985052995
H 6.962685715 -2.460882435 0.700123105
H 7.568301369 -0.878911248 0.186138053
H 7.123511899 -1.153643583 1.875148286
H -0.430859176 -1.064996736 2.9979443
H -0.909165339 -2.753352032 2.763874552
H 0.530475728 -2.345383168 3.73444447
H -3.07059106 -3.909511623 -3.376709139
H -3.251636165 -2.289712397 -4.05583798

H -1.648857978 -3.019601979 -3.91284337

28M$_{1b}$ (0.333 nN at 1-4, C_1)

N 3.999524372 0.589284012 -0.525246333
N 0.704795236 3.211113665 -0.4431326
N -3.27326669 1.567710327 0.618863367
N -3.827943358 -0.914940313 -0.327867105
N -1.055126236 -2.131543182 0.017335773
N 3.118136133 -1.527629704 0.740761762
C 5.507400067 -0.996718372 0.539201276
C 5.270057453 0.092468764 -0.276143439
C 6.183221496 1.05759589 -0.870322845
C 5.452532306 2.09244965 -1.372541539
C 4.037364532 1.834182759 -1.123146376
C 2.955643981 2.677126111 -1.270793099
C 1.638406061 2.284035284 -0.858656943
C 1.01321573 1.021833307 -0.749739113
C -0.279900396 1.211166071 -0.254960025
C -0.481277448 2.59069397 -0.039446151
C -1.534158161 3.339917484 0.550602873
C -2.779211572 2.839681938 0.917829534
C -3.817059482 3.596921598 1.610601374
C -4.910133936 2.79232853 1.698375932
C -4.560757432 1.536969464 1.047621476
C -5.502232993 0.489825028 0.775724517
C -5.135963369 -0.628826701 0.060381762
C -5.967794371 -1.634636685 -0.574307963
C -5.184500313 -2.40009847 -1.382728346
C -3.802343745 -1.973349522 -1.239021594
C -2.680927792 -2.499373136 -1.836793592
C -1.354519172 -2.315895479 -1.32098943
C -0.122806669 -2.477434614 -1.987444293
C 0.901971509 -2.38344689 -1.046757942
C 0.322090541 -2.183722961 0.225527346
C 0.927107539 -2.282672334 1.511749771
C 2.298563649 -2.150330827 1.677860855
C 3.131906989 -2.686030641 2.746657976
C 4.429810463 -2.433371415 2.401357596
C 4.395590725 -1.664252346 1.160950426
H 3.196418836 0.074730771 -0.158083592
H 0.888054267 4.197669983 -0.373235716
H -3.127188178 -0.176715321 -0.205933626
H -1.757106316 -2.025227242 0.735105592
H 7.258280992 0.968611639 -0.876252677
H 5.843359844 2.976396843 -1.850794517
H 1.439783416 0.077988107 -1.04537859

H -1.010985857 0.449409718 -0.049808258<br>H -3.735337135 4.600976461 1.994785076<br>H -5.861970571 3.028264081 2.148332581<br>H -7.033697503 -1.727487749 -0.440231669<br>H -5.505495101 -3.233050336 -1.987819454<br>H -0.011031785 -2.648673034 -3.045895759<br>H 1.963588657 -2.469206976 -1.209044528<br>H 2.787014077 -3.235091882 3.609882054<br>H 5.31841276 -2.710208244 2.948701464<br>C 6.907789181 -1.415053011 0.923360428<br>C 3.144385327 4.103772306 -1.726330947<br>C -1.228217733 4.806536004 0.772599718<br>C -6.940864396 0.722097276 1.18644021<br>C -2.800436825 -3.365659356 -3.067238792<br>C 0.03391804 -2.611060918 2.680081004<br>H 7.030449544 -2.497636381 0.814837562<br>H 7.685079698 -0.936807588 0.316994645<br>H 7.117600463 -1.169502287 1.971833471<br>H 3.118073622 4.808314999 -0.882617642<br>H 4.104157125 4.243288349 -2.224916019<br>H 2.358638815 4.396526325 -2.430696618<br>H -0.345639741 4.931387094 1.410633911<br>H -1.025350582 5.3021464 -0.18526956<br>H -2.053935281 5.339152647 1.234993316<br>H -7.021407283 0.818120013 2.275351<br>H -7.323979921 1.651412695 0.751217998<br>H -7.619648709 -0.080371653 0.883024879<br>H -2.397449934 -4.369408059 -2.897792049<br>H -3.83679664 -3.452163357 -3.395665155<br>H -2.238530476 -2.925435279 -3.899378551<br>H -0.668361635 -1.786950909 2.861816718<br>H -0.555284642 -3.51581962 2.495930193<br>H 0.608070903 -2.745144492 3.596401843
**28M$_{1b}$** (0.333 nN at 1-5, C_1)<br><br>N 4.004307236 0.599290333 -0.509343927<br>N 0.743029627 3.208516065 -0.595547887<br>N -3.164899445 1.532522123 0.627477863<br>N -3.774668519 -0.937837331 -0.351696869<br>N -1.114280647 -2.139372626 -0.128500906<br>N 3.057947539 -1.507136654 0.744666091<br>C 5.446120068 -0.932640921 0.715624058<br>C 5.254579281 0.123052508 -0.155477254<br>C 6.203978973 1.071049479 -0.716979067<br>C 5.505867061 2.081728604 -1.308775514<br>C 4.078838974 1.823777111 -1.145661475

C 3.006426121 2.659571732 -1.376275983
C 1.678126379 2.27554548 -0.99443462
C 1.055221226 1.014511347 -0.866918999
C -0.234501209 1.208156359 -0.366564756
C -0.435866223 2.590723354 -0.170517834
C -1.477722535 3.34165084 0.434805339
C -2.689834762 2.822220017 0.873867032
C -3.695051459 3.560163125 1.632440936
C -4.748436541 2.721561091 1.82289381
C -4.411156704 1.468485091 1.160006555
C -5.340979453 0.398540895 0.968225916
C -5.037532987 -0.685513927 0.173679802
C -5.920000413 -1.672826205 -0.420660907
C -5.212880212 -2.3951422 -1.33427791
C -3.826326878 -1.967486928 -1.296088627
C -2.743732328 -2.49050171 -1.964987194
C -1.404189122 -2.328433382 -1.469071877
C -0.164398768 -2.491804262 -2.120904706
C 0.852393605 -2.39013067 -1.16703179
C 0.257403974 -2.185753413 0.097361288
C 0.823049242 -2.261560914 1.405487698
C 2.181730785 -2.115140986 1.640343706
C 2.943954077 -2.601029522 2.785919126
C 4.257625343 -2.332246477 2.531836991
C 4.303146352 -1.602908263 1.267247406
H 3.180979378 0.09007476 -0.18386192
H 0.929282652 4.194845555 -0.528849594
H -3.07191694 -0.195472461 -0.275847681
H -1.824378246 -2.024070308 0.579855732
H 7.276887686 0.987895432 -0.63815467
H 5.923976067 2.951806831 -1.789578698
H 1.481288341 0.066664858 -1.150651006
H -0.961602143 0.448513317 -0.140875541
H -3.613725434 4.5728847 1.993629433
H -5.669922226 2.934456081 2.342258834
H -6.96857744 -1.78024312 -0.193261231
H -5.58484137 -3.20474034 -1.942119361
H -0.040077183 -2.66636188 -3.177775881
H 1.916593152 -2.47377782 -1.31731893
H 2.545746723 -3.127262972 3.640165696
H 5.106429027 -2.565647738 3.156992141
C 6.839607731 -1.290899232 1.183931556
C 3.215574074 4.072244178 -1.863804829
C -1.191549783 4.81782092 0.610489877
C -6.72840619 0.554114004 1.542512379
C -2.94077135 -3.332804671 -3.203319659
C -0.121599625 -2.570507473 2.540169223

H 7.559779932 -1.185168722 0.36744168
H 7.200823122 -0.665317517 2.015139628
H 6.878554391 -2.332088367 1.513154834
H 3.170756174 4.799725089 -1.040542208
H 4.189488794 4.192611429 -2.339927025
H 2.450835263 4.349591852 -2.596547618
H -0.315001299 4.97715455 1.249707552
H -0.989203083 5.283722512 -0.362017501
H -2.029102061 5.353151388 1.048536643
H -6.681952803 0.758427082 2.617298826
H -7.270450162 1.383913344 1.074920542
H -7.321193515 -0.351974538 1.411738612
H -2.567818631 -4.352819366 -3.062364096
H -3.99524001 -3.381820487 -3.499948737
H -2.391176701 -2.901225049 -4.048337383
H -0.832569841 -1.744573791 2.674550896
H -0.700178744 -3.480320394 2.347387498
H 0.409139582 -2.684962516 3.484361301

28M$_{1b}$ (0.333 nN at 2-5, C_1)

N 4.060914284 0.675745261 -0.50889851
N 0.797514694 3.325158133 -0.49099928
N -3.197981899 1.575919776 0.493056756
N -3.835026293 -0.997146661 -0.372128442
N -1.149212751 -2.146814881 -0.097008748
N 3.053794391 -1.491109391 0.635002663
C 5.463701832 -1.021623956 0.520236306
C 5.300098761 0.116389852 -0.241462027
C 6.273510598 1.088191694 -0.719215036
C 5.603580936 2.190680475 -1.156152017
C 4.17083362 1.965693594 -0.994338912
C 3.121061392 2.847836413 -1.133093381
C 1.769488924 2.428176753 -0.879031859
C 1.141771859 1.158420375 -0.895203921
C -0.184605259 1.314631735 -0.483198878
C -0.409738464 2.679334619 -0.20766231
C -1.503339622 3.404375496 0.336916636
C -2.732664302 2.878141199 0.7177263
C -3.7639566 3.634705513 1.421877012
C -4.818120469 2.798977263 1.607660701
C -4.456314981 1.526282822 0.997526959
C -5.385726058 0.444336946 0.858187791
C -5.088522899 -0.69484604 0.147797439
C -5.976787793 -1.742576 -0.327332118
C -5.280277617 -2.563872021 -1.158209632
C -3.89360994 -2.129462758 -1.193586137

C -2.823070017 -2.710568362 -1.826665264
C -1.473087691 -2.424846752 -1.41188193
C -0.25404826 -2.546986585 -2.104794583
C 0.790105094 -2.344234033 -1.195694971
C 0.229314438 -2.122599706 0.081199243
C 0.8302209 -2.195852916 1.372975685
C 2.197423093 -2.085485915 1.559459564
C 2.98930063 -2.643470781 2.64840142
C 4.300284549 -2.437022053 2.328428684
C 4.313753724 -1.667105799 1.08846286
H 3.225383242 0.179674363 -0.197312165
H 0.966852644 4.305506416 -0.346279748
H -3.111899082 -0.275905408 -0.351761511
H -1.838301441 -2.041550915 0.632728178
H 7.343881824 0.957645878 -0.691689453
H 6.045166881 3.092721566 -1.548618592
H 1.588904918 0.229500108 -1.209834767
H -0.926383273 0.540699998 -0.380799417
H -3.698456513 4.657093707 1.756890601
H -5.75347096 3.024278033 2.095346831
H -7.022297323 -1.825280324 -0.077748738
H -5.661638692 -3.433780461 -1.668457191
H -0.161477786 -2.76517945 -3.156905905
H 1.851005256 -2.387863733 -1.380279258
H 2.607805686 -3.178351293 3.504977012
H 5.169326445 -2.740811267 2.892404793
C 6.834149394 -1.501731606 0.923529066
C 3.369278135 4.315351081 -1.399698157
C -1.244245522 4.885567439 0.532689451
C -6.772379382 0.638213838 1.425504091
C -3.015264626 -3.736362295 -2.919621433
C -0.077644336 -2.503500574 2.53694267
H 7.616542343 -1.033884195 0.323262864
H 7.044716105 -1.276289725 1.976210989
H 6.922332361 -2.585280277 0.797425027
H 3.044836574 4.928954589 -0.546834311
H 4.429381732 4.546699385 -1.54502046
H 2.820540686 4.662265978 -2.282378954
H -0.453410362 5.054821684 1.273726477
H -0.926387838 5.341627516 -0.412484209
H -2.128787608 5.42380868 0.858327822
H -6.723939744 0.889537699 2.490204911
H -7.306069393 1.45153558 0.921195295
H -7.375266535 -0.265522048 1.335177288
H -2.351263306 -4.592575553 -2.77012602
H -4.04517932 -4.110013223 -2.963059926
H -2.783902437 -3.310421463 -3.90398619

H -0.796250775 -1.686045447 2.68008664<br>H -0.647751869 -3.424428395 2.372922759<br>H 0.482756066 -2.596940913 3.466290409
**28M$_{1b}$** (0.333 nN at 2-6, C_1)<br><br>N 3.94883788 0.635889928 -0.491832425<br>N 0.716101539 3.256067076 -0.614453877<br>N -3.185995922 1.583125717 0.618249863<br>N -3.760581166 -0.905572454 -0.325838564<br>N -1.09889274 -2.181961115 -0.018077203<br>N 3.007771318 -1.48475656 0.839401033<br>C 5.396423724 -0.927985535 0.688072563<br>C 5.19928155 0.131377201 -0.173277289<br>C 6.152452265 1.045955204 -0.787599625<br>C 5.461748298 2.060261063 -1.377833382<br>C 4.034842571 1.843929145 -1.166980329<br>C 2.979144584 2.693604993 -1.413409963<br>C 1.647599793 2.321967543 -1.016717006<br>C 1.026113963 1.061927339 -0.879590079<br>C -0.260647599 1.256882941 -0.369936379<br>C -0.46076942 2.638920837 -0.179300126<br>C -1.502726168 3.393513689 0.425471247<br>C -2.716067034 2.876916375 0.859998947<br>C -3.734525441 3.618983416 1.597127166<br>C -4.790056981 2.780606911 1.775863397<br>C -4.439337555 1.521595588 1.129764448<br>C -5.364492474 0.446999556 0.928987984<br>C -5.038666613 -0.645298417 0.156244853<br>C -5.90588267 -1.63341997 -0.46305066<br>C -5.173843478 -2.35854849 -1.353451585<br>C -3.786473862 -1.935444693 -1.271706865<br>C -2.684583093 -2.453979572 -1.905656357<br>C -1.359852099 -2.293705641 -1.372160126<br>C -0.112716556 -2.410514272 -2.01243089<br>C 0.886348143 -2.354805821 -1.036883834<br>C 0.267939418 -2.229407642 0.22520779<br>C 0.838602686 -2.385475269 1.52589999<br>C 2.190984035 -2.17847874 1.735282196<br>C 3.019188777 -2.715429503 2.809423986<br>C 4.311331049 -2.400804458 2.506370005<br>C 4.275754368 -1.597707695 1.284915708<br>H 3.121121286 0.16660507 -0.125992098<br>H 0.900542991 4.243368182 -0.557233232<br>H -3.059203994 -0.164180299 -0.234006842<br>H -1.823480301 -2.102051638 0.67996169<br>H 7.225008889 0.938152908 -0.751373284

H 5.883760147 2.906121586 -1.896955709
H 1.450297007 0.11281584 -1.162013925
H -0.982841877 0.496027773 -0.131697522
H -3.661520073 4.635273204 1.950113056
H -5.72087149 2.996470355 2.27708793
H -6.96158775 -1.736270119 -0.268527364
H -5.529005877 -3.166822646 -1.973231482
H 0.029346362 -2.524624995 -3.075168879
H 1.952842437 -2.421398775 -1.173318101
H 2.673863002 -3.304910334 3.645330015
H 5.197701524 -2.659673416 3.065873476
C 6.78294285 -1.335305755 1.117930353
C 3.191797417 4.096798988 -1.937843341
C -1.214550075 4.869474198 0.597983759
C -6.766658606 0.610782363 1.463420308
C -2.820266652 -3.2921946 -3.15093273
C -0.056708864 -2.869533473 2.643187042
H 7.551440086 -0.852037701 0.512544034
H 6.971402643 -1.068940311 2.1650845
H 6.920415945 -2.417550716 1.026870702
H 3.140921173 4.839561705 -1.128378223
H 4.162204464 4.236080502 -2.42573213
H 2.419022131 4.353653232 -2.670074478
H -0.330703792 5.027512609 1.22718523
H -1.022908098 5.335783706 -0.376558816
H -2.046415094 5.404889228 1.046687654
H -6.749645332 0.820523946 2.538186027
H -7.292595332 1.440156278 0.976972134
H -7.358894692 -0.294023436 1.320990012
H -2.437099646 -4.306892972 -3.000966348
H -3.859137871 -3.351201481 -3.479462405
H -2.248233515 -2.847580878 -3.973922709
H -0.905280191 -2.186563654 2.774854403
H -0.468124742 -3.872489608 2.45587043
H 0.478523391 -2.889875262 3.592467083

28M$_{1b}$ (0.333 nN at 3-5, C_1)

N 3.959524132 0.596824895 -0.564584397
N 0.739715837 3.252161207 -0.477460414
N -3.17538946 1.598206597 0.620227766
N -3.752668446 -0.925995492 -0.421553386
N -1.089486199 -2.15837138 -0.069384378
N 3.037586477 -1.486127965 0.752761583
C 5.425502731 -0.961669914 0.589337998
C 5.217512674 0.09465873 -0.273855405
C 6.15453672 1.027029606 -0.886584126

C 5.446907514 2.052775614 -1.436659571
C 4.024581543 1.823745467 -1.198573871
C 2.959437895 2.680030669 -1.369927269
C 1.643007929 2.311387193 -0.929324811
C 1.007081224 1.058112882 -0.798218519
C -0.262877645 1.264935572 -0.253392007
C -0.436291731 2.646073637 -0.030662633
C -1.464479163 3.393731648 0.607733072
C -2.700525576 2.870896835 0.96249561
C -3.742289458 3.585942308 1.690502636
C -4.817339402 2.756744502 1.766269433
C -4.44926146 1.527361343 1.070797623
C -5.372337157 0.465272704 0.79802756
C -5.036404346 -0.628995377 0.032617754
C -5.907100211 -1.625789416 -0.567831299
C -5.170636381 -2.405851652 -1.404502112
C -3.777696016 -2.005483085 -1.316953716
C -2.687113983 -2.593073384 -1.912883226
C -1.35664327 -2.405840788 -1.403267828
C -0.110354368 -2.584779362 -2.0336297
C 0.892581721 -2.434825457 -1.072690346
C 0.278915751 -2.186515877 0.174387082
C 0.835839414 -2.225150789 1.487031807
C 2.195460272 -2.070483455 1.695347125
C 2.999519259 -2.549723077 2.812654566
C 4.304776707 -2.30705751 2.491489468
C 4.300013911 -1.597729287 1.214510403
H 3.14293967 0.10739513 -0.196599188
H 0.935203738 4.23753327 -0.421456751
H -3.031809689 -0.208218863 -0.334245219
H -1.809890578 -2.02619537 0.625481821
H 7.228335978 0.925476054 -0.871317365
H 5.856891417 2.915326789 -1.93758735
H 1.408360997 0.106759812 -1.107712426
H -0.994091707 0.515772388 -0.009134732
H -3.671912042 4.58234564 2.097076523
H -5.769988926 2.961008281 2.22965687
H -6.968100659 -1.703295413 -0.39318067
H -5.52751477 -3.23656093 -1.992674074
H 0.030076048 -2.799433974 -3.080711077
H 1.958516319 -2.514361603 -1.20664757
H 2.631997721 -3.058525269 3.691079264
H 5.179620207 -2.552008876 3.074962064
C 6.813043435 -1.370581171 1.010696667
C 3.165916594 4.091397944 -1.861865246
C -1.133243439 4.848324126 0.884544448
C -6.791040137 0.636340469 1.288788531

C -2.852835422 -3.529678885 -3.088709502
C -0.097413295 -2.516093193 2.633469956
H 7.577622724 -0.896958152 0.392408847
H 7.012305525 -1.093418268 2.053126473
H 6.944841137 -2.454381858 0.930770904
H 3.157556864 4.817340738 -1.036048098
H 4.123879129 4.20313914 -2.371287965
H 2.378962925 4.378990146 -2.56679251
H -0.182907339 4.926855113 1.424830287
H -1.026666115 5.397394015 -0.060225857
H -1.884490008 5.375276581 1.474228685
H -6.807004272 0.815653171 2.369026471
H -7.284560144 1.488900751 0.808847599
H -7.395226149 -0.251417688 1.101450433
H -2.63668125 -4.579878729 -2.842198819
H -3.863934092 -3.473723648 -3.495625137
H -2.16843177 -3.244160369 -3.895191821
H -0.811163914 -1.691111524 2.75818275
H -0.673093498 -3.432161894 2.463300618
H 0.444096315 -2.610333742 3.574334525

28M$_{1b}$ (0.333 nN at 3-6, C_1)

N 3.963923122 0.636438209 -0.260253927
N 0.604984107 3.339890416 -0.613162899
N -3.318625577 1.843062601 0.5666107
N -3.756606692 -0.76317082 -0.163224739
N -0.98137259 -2.315647389 0.087296856
N 3.147395938 -1.63559957 0.874868498
C 5.509464727 -0.978601861 0.711948519
C 5.242758997 0.153883228 -0.026541247
C 6.141390727 1.12774275 -0.626300433
C 5.395111352 2.134848894 -1.155206678
C 3.983493404 1.861533879 -0.904509604
C 2.901459644 2.682339056 -1.136694749
C 1.570866416 2.356218548 -0.713973515
C 0.948212726 1.154655377 -0.303761371
C -0.370974446 1.442461475 0.051193136
C -0.597050803 2.823216585 -0.132817186
C -1.705223472 3.664722911 0.162240914
C -2.935150386 3.186253957 0.586733176
C -4.035703363 3.98717123 1.109316804
C -5.0480475 3.126431186 1.404965903
C -4.589117872 1.793524187 1.023147408
C -5.436637319 0.640807178 0.958865726
C -5.042055059 -0.507470848 0.307940004
C -5.881559026 -1.566017574 -0.232253131

C	-5.140273239	-2.31309462	-1.092717166
C	-3.763554627	-1.841607258	-1.057084871
C	-2.678399117	-2.355252836	-1.718981645
C	-1.320247545	-2.20310261	-1.249281841
C	-0.114002025	-2.18766196	-1.967726475
C	0.941511417	-2.278894111	-1.053701977
C	0.398162579	-2.393102454	0.241733531
C	1.066902511	-2.806798108	1.442016951
C	2.409152808	-2.549479359	1.631694223
C	3.308980554	-3.245865839	2.543966045
C	4.567132552	-2.797644297	2.275591458
C	4.438151576	-1.772081061	1.239647118
H	3.17262938	0.096534838	0.083225006
H	0.771871594	4.313106742	-0.80410887
H	-3.071760676	-0.005065495	-0.125040908
H	-1.664793943	-2.345575196	0.828239355
H	7.217338288	1.056005973	-0.633682302
H	5.774368292	3.011609275	-1.654597093
H	1.377406787	0.165972594	-0.302265067
H	-1.103000838	0.753375448	0.433589564
H	-4.032895071	5.055450112	1.26124267
H	-6.016863027	3.369273527	1.813584247
H	-6.93059942	-1.690234849	-0.015428146
H	-5.475371181	-3.165532356	-1.662445591
H	-0.032704459	-2.11819148	-3.040605122
H	1.99835149	-2.302856935	-1.261838353
H	3.023228629	-4.008344561	3.253204191
H	5.48956562	-3.10911153	2.742170455
C	6.924337529	-1.380715241	1.042100496
C	3.100728545	4.047029231	-1.753292269
C	-1.477388314	5.154324617	-0.001655775
C	-6.856010387	0.780846145	1.455680965
C	-2.85756315	-3.220674928	-2.94274871
C	0.340455897	-3.694684133	2.432172258
H	7.653313127	-0.775847547	0.50097745
H	7.130949013	-1.265096885	2.112861585
H	7.106758459	-2.429535512	0.785900805
H	3.003127631	4.848139887	-1.00719711
H	4.086525958	4.149583716	-2.20519591
H	2.360714933	4.230609395	-2.54055001
H	-0.698221142	5.511956848	0.682261636
H	-1.14644472	5.369820864	-1.025771532
H	-2.374841027	5.754243757	0.162878227
H	-6.869285474	1.200378882	2.466550735
H	-7.449049782	1.446092023	0.816900909
H	-7.363334731	-0.183805061	1.500838945
H	-2.508600772	-4.24503954	-2.772773085

H -3.899912413 -3.24781083 -3.264561982
H -2.273160596 -2.817218118 -3.777923408
H -0.742014913 -3.661858766 2.296348393
H 0.646506071 -4.746143562 2.309386211
H 0.562959267 -3.40396216 3.461288454

28M$_{1b}$ (0.333 nN at 5-6, C_1)

N 3.944787532 0.550435263 -0.569880205
N 0.700647361 3.182384335 -0.545315003
N -3.19205811 1.521698394 0.672750574
N -3.754685706 -0.909946102 -0.371521754
N -1.095997463 -2.105712703 -0.021843924
N 3.03141442 -1.495812789 0.797630308
C 5.419732256 -0.98469533 0.602705803
C 5.206276316 0.055832595 -0.281255725
C 6.135197014 0.976441319 -0.917867329
C 5.419666501 1.990886792 -1.482659821
C 4.001781457 1.76142906 -1.230529902
C 2.928968587 2.61123578 -1.413942597
C 1.618393471 2.24283363 -0.970133517
C 0.995550169 0.98332091 -0.810718878
C -0.274204804 1.185856069 -0.270861368
C -0.466919293 2.57145082 -0.079761452
C -1.494626272 3.319633774 0.54580701
C -2.714651568 2.797099655 0.967244131
C -3.73236653 3.521292225 1.719952474
C -4.801093628 2.68834284 1.852571588
C -4.45675195 1.453118508 1.163407117
C -5.385518524 0.400583592 0.892212885
C -5.047597376 -0.662214581 0.080357721
C -5.899074704 -1.612614651 -0.608053167
C -5.139688695 -2.308062027 -1.503248623
C -3.756420413 -1.902441429 -1.352879427
C -2.634649022 -2.420169027 -1.96316444
C -1.333779978 -2.313080729 -1.373066492
C -0.072863188 -2.546672971 -1.964868127
C 0.901448059 -2.463546028 -0.974480155
C 0.265451065 -2.194483102 0.261521828
C 0.827725773 -2.207306493 1.57025931
C 2.198657036 -2.065761982 1.752168169
C 3.011618768 -2.540787065 2.863681405
C 4.316135876 -2.31048812 2.525663823
C 4.301849358 -1.611726607 1.245370881
H 3.134033099 0.065256605 -0.180486417
H 0.88822427 4.169807775 -0.500388774
H -3.061831624 -0.163848135 -0.238545207

H -1.830592605 -1.946322408 0.652770388
H 7.20936749 0.877911187 -0.91003468
H 5.823382194 2.843894394 -2.004458103
H 1.409291367 0.03140289 -1.099920711
H -0.994273897 0.431003318 -0.013088766
H -3.652547591 4.522875336 2.111603034
H -5.736559839 2.896705608 2.348491135
H -6.961624733 -1.714845132 -0.454488697
H -5.475522533 -3.087766466 -2.168872357
H 0.090948149 -2.755092652 -3.009642936
H 1.965852945 -2.589280708 -1.080263
H 2.650294387 -3.04159247 3.749265721
H 5.195351836 -2.557632807 3.101680284
C 6.811532286 -1.38072396 1.022515547
C 3.131645184 4.018320357 -1.919656577
C -1.200611278 4.790362554 0.745235241
C -6.800494805 0.553850441 1.39422627
C -2.750014998 -3.1960141 -3.256575495
C -0.073934658 -2.428488209 2.759270113
H 7.570058466 -0.922074168 0.385858031
H 7.018236458 -1.076580862 2.055815469
H 6.943554591 -2.466022024 0.969420478
H 3.133136907 4.751084002 -1.099858493
H 4.08421507 4.124364735 -2.440335057
H 2.337635739 4.300954979 -2.618678034
H -0.292254236 4.933080023 1.342098932
H -1.047992848 5.283039072 -0.223541135
H -2.014408385 5.312264583 1.241199298
H -6.81090425 0.720798381 2.476448442
H -7.305766592 1.406606713 0.927009268
H -7.394791085 -0.339732373 1.19935324
H -2.542915463 -4.262357531 -3.112460222
H -3.736869264 -3.089227641 -3.720933235
H -2.020876466 -2.822210961 -3.983739735
H -0.798044094 -1.607219762 2.84072538
H -0.640771561 -3.361875201 2.672596554
H 0.496263541 -2.441870928 3.694436512

28M₂ (0.333 nN at 1-4, C_1)

N 3.971306506 0.552213029 -0.562809668
N 0.688575743 3.189441951 -0.592096221
N -3.262763032 1.599285796 0.641636782
N -3.696138926 -0.945877189 -0.26174499
N -1.066593747 -2.051693402 -0.010130424
N 3.059849395 -1.48141508 0.797194954
C 5.452526365 -0.975469633 0.618273789

C 5.232831141 0.070571865 -0.264951118
C 6.159493608 1.005292056 -0.882839175
C 5.439187545 2.017905005 -1.446890639
C 4.021951783 1.772603553 -1.21321306
C 2.942436578 2.612021207 -1.399634194
C 1.621221829 2.24029293 -0.983670839
C 0.990636088 0.991516885 -0.82954872
C -0.300060161 1.204325227 -0.326924934
C -0.490184841 2.591553334 -0.153406454
C -1.530541796 3.373727991 0.435908741
C -2.751809318 2.877277891 0.853519234
C -3.783744561 3.57701439 1.597696964
C -4.840324982 2.738368965 1.793146555
C -4.545535253 1.472792659 1.155250805
C -5.38945174 0.395568585 0.9309872
C -4.979529694 -0.681843845 0.095517357
C -5.898055684 -1.538008957 -0.652143641
C -5.145043531 -2.253329905 -1.531748184
C -3.75173361 -1.908632572 -1.275842532
C -2.634638182 -2.439558176 -1.893493286
C -1.326316944 -2.332721324 -1.339268311
C -0.081417819 -2.626959369 -1.939896444
C 0.908847417 -2.502553737 -0.970779442
C 0.286825611 -2.1570555 0.255161239
C 0.850073204 -2.16676267 1.569142165
C 2.215482198 -2.033001392 1.756476448
C 3.024652372 -2.49775131 2.881345007
C 4.329147083 -2.282946775 2.545509898
C 4.328578944 -1.596703932 1.251805282
H 3.160742713 0.0542737 -0.186276829
H 0.877206099 4.176521775 -0.54736617
H -2.881952917 0.84201063 0.08059323
H -1.80652663 -1.74739059 0.612919874
H 7.234309514 0.916594051 -0.859326144
H 5.841567375 2.879576941 -1.955513381
H 1.406195595 0.032433783 -1.093906187
H -1.005188948 0.422969461 -0.09871053
H -3.711766649 4.592788743 1.950292343
H -5.758748982 2.969715511 2.307954452
H -6.972714098 -1.546107882 -0.548234703
H -5.496777034 -2.976964151 -2.25094588
H 0.059939171 -2.904080853 -2.972242246
H 1.968893185 -2.660519473 -1.077295265
H 2.655397676 -2.979997761 3.773924056
H 5.204531496 -2.528025989 3.12842841
C 6.846263285 -1.355822868 1.064557895
C 3.142360932 4.020905607 -1.903068159

C -1.224330715 4.843291018 0.620551562<br>C -6.812300088 0.42146514 1.450889073<br>C -2.7520125 -3.207023661 -3.18630341<br>C -0.083751003 -2.404999235 2.726352801<br>H 6.943805262 -2.443501051 1.135947608<br>H 7.628706611 -1.006633669 0.381094928<br>H 7.073481236 -0.943304265 2.055771755<br>H 3.108971023 4.753134368 -1.083726404<br>H 4.108577203 4.139657208 -2.394375898<br>H 2.365014941 4.291528289 -2.625005737<br>H -0.341970948 4.989307678 1.255352392<br>H -1.025360966 5.321077899 -0.347532117<br>H -2.052307216 5.385089647 1.072179003<br>H -7.134396486 -0.587587765 1.722624834<br>H -6.887181509 1.034249614 2.352066752<br>H -7.544991887 0.813077633 0.728085758<br>H -3.776793362 -3.214645712 -3.555789316<br>H -2.130818605 -2.740836309 -3.959307448<br>H -2.408908902 -4.240200825 -3.071769793<br>H -0.774901039 -1.559007184 2.835082009<br>H -0.688282861 -3.305579941 2.576697902<br>H 0.459866341 -2.494422706 3.666911164
**28M$_2$** (0.333 nN at 1-5, C$_1$)<br><br>N 3.978045688 0.586010484 -0.567262782<br>N 0.718991601 3.177495396 -0.68557713<br>N -3.158694466 1.534249683 0.681023911<br>N -3.668587819 -1.000703199 -0.276256984<br>N -1.115217401 -2.080412916 -0.136752655<br>N 3.022132516 -1.452492833 0.772151431<br>C 5.408087718 -0.88101927 0.747408888<br>C 5.222974793 0.13461989 -0.178557511<br>C 6.175086103 1.065599943 -0.761034407<br>C 5.478384914 2.051081835 -1.398964896<br>C 4.053186566 1.791427872 -1.243345662<br>C 2.97626691 2.616438308 -1.492515116<br>C 1.650627548 2.236733279 -1.097806446<br>C 1.030461219 0.983446231 -0.946418569<br>C -0.251458557 1.183313448 -0.41702822<br>C -0.444060932 2.567542334 -0.224739406<br>C -1.464214612 3.334856993 0.416001079<br>C -2.652842922 2.81521857 0.890712599<br>C -3.642208219 3.482978404 1.716720589<br>C -4.67137632 2.624355993 1.961558957<br>C -4.40489183 1.379689974 1.271484103<br>C -5.260567813 0.310759986 1.065787844

C -4.922737359 -0.735783495 0.162959206
C -5.894144951 -1.552559381 -0.561319819
C -5.200519515 -2.250641677 -1.502597702
C -3.791053043 -1.934495085 -1.312449136
C -2.708431974 -2.4737466 -1.98385823
C -1.382897222 -2.375433585 -1.461210014
C -0.136250038 -2.660525856 -2.065177007
C 0.859473241 -2.513035506 -1.100116619
C 0.237447254 -2.165440192 0.125569927
C 0.776596923 -2.149395108 1.451600253
C 2.130398134 -1.996562842 1.693247351
C 2.877068811 -2.411933909 2.880690964
C 4.193677425 -2.171918145 2.625475354
C 4.261315675 -1.519636035 1.315599112
H 3.153042926 0.083669263 -0.232121887
H 0.906460715 4.164102129 -0.626005606
H -2.811333495 0.80626209 0.064060794
H -1.85706766 -1.763327594 0.479805923
H 7.247006136 0.992790917 -0.661464912
H 5.898576142 2.906598436 -1.903759034
H 1.446980782 0.028655133 -1.225000771
H -0.944424917 0.394356428 -0.178918004
H -3.55771885 4.490385588 2.089987459
H -5.55522773 2.828496319 2.54381542
H -6.961886954 -1.549250972 -0.4017103
H -5.603832092 -2.944919298 -2.22361969
H 0.003770152 -2.94406043 -3.096404871
H 1.921563554 -2.659666366 -1.209902953
H 2.462488292 -2.878256512 3.761535354
H 5.033207447 -2.373065734 3.273963767
C 6.798654266 -1.20404572 1.249885979
C 3.180865223 4.018266866 -2.011873296
C -1.166254427 4.805096013 0.60562611
C -6.653060297 0.341505021 1.651550738
C -2.896294554 -3.231585815 -3.275920231
C -0.195173519 -2.371830573 2.582669439
H 7.516799534 -1.197853166 0.424182098
H 7.172976076 -0.497434102 2.007210969
H 6.825913372 -2.204011115 1.688958877
H 3.130449024 4.762599849 -1.204305802
H 4.15605183 4.132161068 -2.486925139
H 2.416183948 4.275817378 -2.751503633
H -0.267021675 4.955175838 1.215540108
H -1.001021053 5.293245359 -0.363533594
H -1.986778201 5.333927215 1.085701119
H -6.676350104 0.893691397 2.593490544
H -7.375464036 0.811649046 0.972585947

H -7.005721936 -0.671245562 1.861805974
H -2.329290038 -2.750372781 -4.081242201
H -2.528614179 -4.259218816 -3.190117698
H -3.945601997 -3.260659842 -3.58759624
H -0.898095861 -1.530760236 2.646680912
H -0.785472307 -3.281776985 2.433037133
H 0.314234321 -2.432687362 3.543950266

28M₂ (0.333 nN at 2-5, C_1)

N 4.030714484 0.641945303 -0.62667438
N 0.789630994 3.301219067 -0.571897012
N -3.179978646 1.555265103 0.567727685
N -3.718516607 -1.062518583 -0.301689839
N -1.156774193 -2.073362879 -0.134539563
N 3.000606366 -1.421993174 0.643117617
C 5.412551086 -0.996485358 0.521325252
C 5.262837818 0.093218625 -0.323993368
C 6.243101945 1.039736543 -0.822692302
C 5.579012548 2.1343879 -1.299285307
C 4.149495704 1.91992644 -1.14157139
C 3.099572893 2.814815874 -1.267752082
C 1.754758953 2.40578482 -1.004475601
C 1.122946765 1.140203345 -1.018333997
C -0.189532467 1.291356884 -0.562211148
C -0.403878683 2.655372441 -0.256048941
C -1.472001381 3.377419477 0.348135503
C -2.677659155 2.840247181 0.773274483
C -3.689465543 3.520419831 1.556926257
C -4.72798114 2.668218167 1.785050489
C -4.443749356 1.413378383 1.130269522
C -5.305010224 0.341790922 0.949814836
C -4.972585316 -0.759681013 0.118740298
C -5.947478541 -1.640329626 -0.521807359
C -5.254118956 -2.436034611 -1.38268907
C -3.847475022 -2.100772613 -1.228485749
C -2.78059762 -2.722237324 -1.85861198
C -1.446006153 -2.527510901 -1.407353729
C -0.199484718 -2.818304141 -2.018794712
C 0.809837971 -2.52650947 -1.108734197
C 0.202353094 -2.091316805 0.101560408
C 0.758969041 -2.008771224 1.409878976
C 2.127894206 -1.908473391 1.606786289
C 2.897978875 -2.377791944 2.7546792
C 4.215349779 -2.237235129 2.428203005
C 4.258469421 -1.581628767 1.122426981
H 3.192789332 0.159690515 -0.293251475

H 0.965709999 4.278266429 -0.412035927
H -2.807345253 0.807256976 -0.007498373
H -1.895515833 -1.718992032 0.465669995
H 7.312626395 0.907503078 -0.777832528
H 6.030743091 3.021185865 -1.714250701
H 1.557070002 0.211656797 -1.354334621
H -0.901127111 0.486632588 -0.468246218
H -3.61608609 4.532362231 1.918452654
H -5.627884014 2.885318282 2.336807811
H -7.015650474 -1.615167994 -0.367838275
H -5.650528401 -3.196767785 -2.038445152
H -0.067996538 -3.193539075 -3.020993163
H 1.873699985 -2.643397678 -1.232328681
H 2.49430938 -2.818381425 3.653796924
H 5.072521954 -2.508266886 3.026418185
C 6.780913306 -1.431417955 0.981874683
C 3.394773098 4.287489324 -1.448542458
C -1.209280806 4.851873039 0.574141371
C -6.702344163 0.416110102 1.523562339
C -3.04049361 -3.688107268 -2.99356535
C -0.187479398 -2.140358697 2.575038063
H 7.55959904 -1.088646306 0.297608747
H 7.017360044 -1.037164406 1.97804938
H 6.846121916 -2.521935824 1.036098773
H 3.183185745 4.840090473 -0.52153407
H 4.450139053 4.451417287 -1.666759068
H 2.821208315 4.763462267 -2.251369296
H -0.385691291 5.009307136 1.282121846
H -0.940455387 5.34088385 -0.370404869
H -2.080815466 5.37602087 0.957643539
H -6.718591403 0.974480605 2.461865774
H -7.404935838 0.901662028 0.835024959
H -7.083829796 -0.584473343 1.738532503
H -3.643297932 -3.224263023 -3.779485611
H -2.106666057 -4.034468151 -3.437784094
H -3.584812649 -4.579366379 -2.644184379
H -0.859103002 -1.272780311 2.615031723
H -0.812437374 -3.035418766 2.48870612
H 0.349394912 -2.171000428 3.523086177

28M₂ (0.333 nN at 2-6, C_1)

N 3.885083243 0.610001677 -0.532501494
N 0.671915575 3.21683923 -0.751404314
N -3.178960144 1.600671155 0.680555591
N -3.640334931 -0.936915717 -0.237949377
N -1.089480249 -2.095207925 -0.001987608

N 2.947783828 -1.426232421 0.928995758
C 5.329006791 -0.866104851 0.768241352
C 5.13083373 0.132172873 -0.168432773
C 6.089887793 1.000500994 -0.835786575
C 5.405228803 1.971249306 -1.501687832
C 3.976583085 1.77442702 -1.287155778
C 2.925452229 2.615067617 -1.585268271
C 1.590338704 2.268765372 -1.17289918
C 0.969600154 1.021054137 -0.995915799
C -0.30138589 1.229827123 -0.439872298
C -0.484419549 2.615126392 -0.260121444
C -1.492246938 3.396410495 0.390237255
C -2.678702227 2.885953903 0.873964516
C -3.680443702 3.563578196 1.679796384
C -4.710981949 2.706890927 1.923001904
C -4.431716102 1.450765634 1.254659006
C -5.277175597 0.375274557 1.045680852
C -4.90843112 -0.673236543 0.154110454
C -5.854177824 -1.478712613 -0.618271182
C -5.127263762 -2.161641684 -1.54547521
C -3.725417187 -1.852484397 -1.295454544
C -2.616613542 -2.371478149 -1.931496645
C -1.313893092 -2.297208001 -1.352437494
C -0.056152987 -2.551222584 -1.937371079
C 0.910382222 -2.474048594 -0.935086659
C 0.253587474 -2.208135298 0.29013038
C 0.785455441 -2.319008485 1.619929815
C 2.127624738 -2.092090389 1.844865041
C 2.950808973 -2.568233132 2.95575024
C 4.239881787 -2.24937984 2.655323593
C 4.210957829 -1.503837548 1.392169684
H 3.052335256 0.163897262 -0.148656344
H 0.857038691 4.204739222 -0.715077141
H -2.816490005 0.858221333 0.090292485
H -1.84186846 -1.80114004 0.611867182
H 7.161895527 0.89564599 -0.782425812
H 5.837770715 2.772059724 -2.079843214
H 1.380851001 0.062731677 -1.270262439
H -0.984376296 0.441126685 -0.171320145
H -3.604844231 4.577998033 2.036010701
H -5.604223942 2.917928782 2.488442195
H -6.927061225 -1.475451215 -0.497014611
H -5.502156435 -2.842659844 -2.294270961
H 0.11310855 -2.770435972 -2.97958004
H 1.973456643 -2.621955739 -1.023046042
H 2.601152558 -3.118299804 3.816107171
H 5.122824866 -2.469588689 3.236620009

C 6.716259566 -1.22580693 1.238739307<br>C 3.172474131 3.980825755 -2.186284497<br>C -1.182076787 4.864978417 0.568853887<br>C -6.683421447 0.401081949 1.596542758<br>C -2.730792852 -3.088953313 -3.25108724<br>C -0.149458321 -2.773253566 2.717101786<br>H 7.484218579 -0.804779151 0.587796489<br>H 6.905530389 -0.855820528 2.253898146<br>H 6.857155186 -2.311204107 1.254763207<br>H 2.858770274 4.779355814 -1.499641554<br>H 4.233920066 4.142793056 -2.37630498<br>H 2.640709498 4.140678252 -3.133956468<br>H -0.26413565 5.009884027 1.151323053<br>H -1.04361531 5.353132602 -0.404604296<br>H -1.9844847 5.397291608 1.075347541<br>H -6.734976257 0.96859233 2.528339701<br>H -7.394099851 0.852832425 0.89295035<br>H -7.03172852 -0.611753634 1.81469354<br>H -3.752994987 -3.071935872 -3.628413974<br>H -2.096693512 -2.601982814 -4.000554642<br>H -2.39917922 -4.129071598 -3.170542205<br>H -0.675516567 -1.914971293 3.154345058<br>H -0.904853692 -3.458649438 2.322894976<br>H 0.370547068 -3.28497447 3.53424312
**28M₂** (0.333 nN at 3-5, C_1)<br><br>N 3.928814001 0.561454617 -0.626795947<br>N 0.726434764 3.208266853 -0.585463396<br>N -3.159350255 1.604420934 0.671440554<br>N -3.641766829 -0.969406253 -0.357169629<br>N -1.102704551 -2.077264879 -0.098671019<br>N 2.980945779 -1.433292719 0.787536189<br>C 5.370979109 -0.923670079 0.644895589<br>C 5.179078506 0.082653916 -0.286209468<br>C 6.125708359 0.988041744 -0.923333677<br>C 5.424857238 1.985736876 -1.533920977<br>C 4.001026601 1.763759033 -1.309954286<br>C 2.934833807 2.612254386 -1.510576077<br>C 1.62023176 2.255634221 -1.052737688<br>C 0.986994166 1.012656472 -0.887182055<br>C -0.267520587 1.229539435 -0.29979755<br>C -0.428395673 2.61370742 -0.087278739<br>C -1.426425842 3.382620304 0.593255441<br>C -2.642061941 2.862791619 0.986120151<br>C -3.663236609 3.498959839 1.79700565<br>C -4.719357545 2.649510955 1.931778088

C -4.434645657 1.435913644 1.190220834<br>C -5.282351448 0.379895543 0.906729298<br>C -4.908711324 -0.667818591 0.014498353<br>C -5.864700688 -1.482746591 -0.735493136<br>C -5.144823323 -2.229406211 -1.61610224<br>C -3.738439985 -1.941886992 -1.36701337<br>C -2.649039751 -2.549993164 -1.963200462<br>C -1.338129445 -2.445080012 -1.409110537<br>C -0.083504596 -2.778007741 -1.966530928<br>C 0.889485594 -2.584614441 -0.988015411<br>C 0.241278193 -2.162355166 0.199473881<br>C 0.760014478 -2.092139126 1.531760104<br>C 2.113959658 -1.936342501 1.755086458<br>C 2.893369534 -2.330453053 2.926650896<br>C 4.20486358 -2.126693047 2.613525494<br>C 4.233394133 -1.513944332 1.282936987<br>H 3.106387354 0.086375415 -0.250958681<br>H 0.92491966 4.193736679 -0.541793142<br>H -2.784226647 0.911524293 0.033384398<br>H -1.857426262 -1.715093185 0.477023125<br>H 7.199296687 0.893098138 -0.879404918<br>H 5.842325453 2.826909443 -2.064442773<br>H 1.374277661 0.052311922 -1.189945937<br>H -0.954229132 0.447585005 -0.02489356<br>H -3.581664033 4.483677255 2.227693757<br>H -5.63072639 2.839661455 2.474802554<br>H -6.938383104 -1.448117645 -0.628869414<br>H -5.527474554 -2.938044279 -2.334665763<br>H 0.080201048 -3.121631032 -2.975251968<br>H 1.951417066 -2.747113014 -1.06427056<br>H 2.50171388 -2.764363045 3.834340304<br>H 5.066247373 -2.332982396 3.231198188<br>C 6.751284416 -1.291947895 1.125336246<br>C 3.140775727 4.007103964 -2.046773627<br>C -1.071145617 4.826229379 0.886792366<br>C -6.703662212 0.404046135 1.421975078<br>C -2.806025638 -3.400665829 -3.202311998<br>C -0.212275542 -2.273313039 2.667198679<br>H 7.52417005 -0.896910127 0.463304856<br>H 6.946079079 -0.900118181 2.131355664<br>H 6.876555341 -2.378403449 1.167612396<br>H 3.120793572 4.75838074 -1.244259166<br>H 4.104096595 4.106383499 -2.548690874<br>H 2.359163189 4.268483623 -2.767282015<br>H -0.107212084 4.886711415 1.405900142<br>H -0.983307935 5.399267861 -0.046079886<br>H -1.802839399 5.348918162 1.507137831

H -6.781902641 0.973290427 2.350171916
H -7.396263081 0.852005678 0.698431374
H -7.055461799 -0.608787042 1.634008325
H -3.786940239 -3.259157748 -3.656139182
H -2.057148823 -3.117491693 -3.948967598
H -2.669863335 -4.47360208 -3.001134919
H -0.911452402 -1.427511239 2.703296691
H -0.806472268 -3.184859021 2.546440984
H 0.298881673 -2.305786101 3.629496061

28M₂ (0.333 nN at 3-6, C_1)

N 3.922036823 0.61493154 -0.339581011
N 0.58376451 3.272652565 -0.762816518
N -3.298926788 1.796746527 0.592675115
N -3.624293253 -0.837257396 -0.109470512
N -0.997991348 -2.162804736 0.084902735
N 3.067254309 -1.532403451 0.961038797
C 5.437223287 -0.921163996 0.790973056
C 5.191312628 0.149481169 -0.050961777
C 6.103916599 1.072693987 -0.70381098
C 5.369208931 2.047074234 -1.311358498
C 3.956942454 1.797016146 -1.062754804
C 2.875117934 2.606540986 -1.338896182
C 1.547370853 2.284404502 -0.912613391
C 0.928610347 1.079488305 -0.526994234
C -0.382098046 1.362395799 -0.126709161
C -0.602133041 2.752136146 -0.25788398
C -1.68458453 3.607257988 0.118557724
C -2.881649954 3.123867076 0.606248359
C -3.946878895 3.853475186 1.267358363
C -4.926561068 2.975584517 1.628465685
C -4.553066652 1.654348687 1.160355843
C -5.325882262 0.509219428 1.076390313
C -4.899806657 -0.597440653 0.283101092
C -5.826145741 -1.474790156 -0.433583149
C -5.090200992 -2.162535989 -1.348150736
C -3.696682175 -1.79193567 -1.135057693
C -2.595907883 -2.283480789 -1.806042062
C -1.272738494 -2.222006927 -1.271163354
C -0.039777817 -2.418345 -1.924349492
C 0.962792082 -2.453964997 -0.956429083
C 0.354476657 -2.327456354 0.313720375
C 0.960446653 -2.610391726 1.587903514
C 2.298878375 -2.353478884 1.788901642
C 3.170602651 -2.965268981 2.791262556
C 4.439991702 -2.57031946 2.502309089

C 4.350567558 -1.648658994 1.363898332
H 3.12183007 0.110373032 0.040858286
H 0.755545393 4.25240337 -0.912433596
H -2.891841765 1.00817447 0.096335534
H -1.71820439 -1.937186589 0.759623109
H 7.179462652 0.996566054 -0.689196374
H 5.759698073 2.885684844 -1.865183355
H 1.353265809 0.0891001 -0.572572113
H -1.07356726 0.627538156 0.250694399
H -3.934452324 4.913709425 1.464606703
H -5.845085683 3.210036347 2.142040037
H -6.895886149 -1.508339378 -0.291374985
H -5.449218793 -2.888570803 -2.061589955
H 0.089598933 -2.527060403 -2.989286495
H 2.020784011 -2.59675316 -1.099434059
H 2.856560875 -3.646055752 3.568421457
H 5.348338257 -2.84924399 3.015245522
C 6.844348697 -1.29248034 1.1861263
C 3.07806985 3.952888396 -1.992101902
C -1.45372793 5.09911818 -0.001952991
C -6.736452875 0.515197731 1.617259297
C -2.742510176 -2.974807766 -3.138050684
C 0.171071066 -3.378969577 2.625746071
H 7.584079405 -0.7563265 0.589515409
H 7.040215626 -1.058795689 2.239629194
H 7.023854351 -2.363929435 1.050018419
H 3.008145569 4.771765832 -1.262359365
H 4.056338775 4.03046179 -2.465857105
H 2.322021568 4.127817153 -2.765118575
H -0.66039795 5.439467983 0.675242682
H -1.14752786 5.350804111 -1.025860988
H -2.347974528 5.695168498 0.199304704
H -6.826440826 1.182057295 2.477647054
H -7.466644743 0.841291238 0.865746976
H -7.027574808 -0.48453222 1.949686703
H -3.776057718 -2.963111033 -3.483103245
H -2.140919748 -2.459125918 -3.895385171
H -2.390798826 -4.010420988 -3.093981673
H 0.434412329 -3.065196675 3.638011481
H -0.904599543 -3.241393869 2.496372145
H 0.370639728 -4.46042919 2.546397506

28M$_2$ (0.333 nN at 5-6, C_1)

N 3.909884526 0.542468479 -0.616143721
N 0.669230347 3.159117633 -0.643078587
N -3.192237686 1.544226676 0.716194025

N -3.644467945 -0.968130882 -0.286241295
N -1.092197062 -2.06895851 -0.022022156
N 2.994033706 -1.440575642 0.835536316
C 5.379665567 -0.924890722 0.646653851
C 5.16787126 0.074227584 -0.289671958
C 6.099802686 0.974518204 -0.951204214
C 5.384427191 1.959715218 -1.567076105
C 3.966534351 1.73148304 -1.322757887
C 2.890892373 2.568255722 -1.534049899
C 1.58052291 2.207811338 -1.076541544
C 0.959960015 0.959676194 -0.889106034
C -0.302174355 1.173292238 -0.32065104
C -0.482838827 2.560655002 -0.141053998
C -1.489555283 3.334509209 0.511853494
C -2.688395429 2.817777514 0.963624704
C -3.69898268 3.474609184 1.771776838
C -4.741524964 2.616966023 1.96427971
C -4.457472904 1.384284609 1.259450352
C -5.305870572 0.322435945 0.990921832
C -4.922605803 -0.701927267 0.080498356
C -5.853292739 -1.479349472 -0.735545359
C -5.106909063 -2.14754682 -1.658749565
C -3.710361161 -1.860329663 -1.362075459
C -2.593712421 -2.39789731 -1.975229168
C -1.306241892 -2.353199684 -1.361470211
C -0.044158227 -2.680260462 -1.908177622
C 0.90899582 -2.562628196 -0.902271646
C 0.248415563 -2.184969371 0.295419411
C 0.79030009 -2.133193858 1.620560409
C 2.152150053 -1.969141167 1.809194293
C 2.959622161 -2.380082216 2.956322005
C 4.262957561 -2.161464594 2.619915557
C 4.259345861 -1.524319661 1.301641151
H 3.096035503 0.06853597 -0.218405176
H 0.859561828 4.146503651 -0.609507566
H -2.827724771 0.826091295 0.097483296
H -1.854259818 -1.734151787 0.560106133
H 7.17437585 0.885697421 -0.921733382
H 5.789652776 2.794022914 -2.117426906
H 1.363220014 -0.000401315 -1.169569764
H -0.986872563 0.390551652 -0.043942879
H -3.6225304 4.475277442 2.164895191
H -5.644420611 2.815260738 2.518927412
H -6.929054179 -1.467990653 -0.644148667
H -5.464884699 -2.806768221 -2.434909017
H 0.135290526 -2.971448571 -2.930606372
H 1.969286037 -2.737413695 -0.969647873

H 2.59086924 -2.835718655 3.862905567
H 5.138432588 -2.374490541 3.215104578
C 6.770716364 -1.279194184 1.106856775
C 3.08673869 3.958037231 -2.087402913
C -1.177401918 4.798167912 0.720722146
C -6.725707755 0.346950235 1.506219068
C -2.695827424 -3.102234188 -3.308707373
C -0.131179371 -2.326331525 2.798363277
H 7.52920466 -0.890482443 0.42480367
H 6.98177117 -0.871664198 2.10323027
H 6.901878686 -2.364275767 1.162510889
H 3.071038068 4.717776118 -1.292844514
H 4.045077902 4.055491325 -2.599000569
H 2.297660318 4.208285151 -2.803699254
H -0.255285073 4.929298853 1.299524626
H -1.044912204 5.307874237 -0.242625051
H -1.976577594 5.319271979 1.244119815
H -6.791109248 0.867434133 2.464360892
H -7.408792249 0.848236136 0.809018032
H -7.097434204 -0.668572616 1.663276713
H -1.928961688 -2.721042967 -3.991003832
H -2.527082425 -4.179655434 -3.203930456
H -3.663683212 -2.945856618 -3.795454117
H -0.864967742 -1.511009193 2.841020653
H -0.690683217 -3.264852446 2.724672244
H 0.420515015 -2.30986833 3.744841927

28H (1.0 nN at 1-4, C_1)

N 3.9957082413 -1.3699481226 0.0495121178
N 0.0660871495 -3.4662767298 0.0091353622
N -3.9075297378 -1.3855738241 -0.011782339
N -4.0024623550 1.3965275526 -0.0872626211
N -0.0579223895 3.4540208137 0.2642860805
N 3.9145475295 1.4032813998 -0.037215971
C 5.9954225649 0.0578212658 -0.2478606069
C 5.3624541586 -1.1539679021 -0.1401545097
C 5.9696591617 -2.4787999455 -0.1791059823
C 5.0007541783 -3.4062105150 -0.0182053353
C 3.7013230478 -2.7355685318 0.1136281490
C 2.4887737478 -3.3655250979 0.2105982083
C 1.1895576038 -2.7229476465 0.3009347622
C 0.7247502918 -1.4547179984 0.6855958640
C -0.6853973092 -1.4605131367 0.6092508220
C -1.1022647087 -2.7249129753 0.1755719333
C -2.3750517524 -3.3651494284 -0.0259615524
C -3.6188658156 -2.7706066944 -0.0528496176

C -4.8821310400 -3.5049172044 -0.1388369986
C -5.8806507559 -2.5898475044 -0.1405248126
C -5.2482654105 -1.2724079105 -0.0731554199
C -6.0094440426 -0.0398353773 -0.0882439816
C -5.3839066974 1.1778589391 -0.0884343087
C -5.9916471744 2.5022541666 -0.0714675679
C -5.0107006191 3.4306992403 -0.0358734441
C -3.7025595535 2.7618685504 -0.0444101636
C -2.4832177024 3.3869008775 0.0015064333
C -1.1819519723 2.7498877941 -0.1145035996
C -0.7149472599 1.5367409307 -0.6474319654
C 0.6954670321 1.5367427018 -0.5766922681
C 1.1122500964 2.7417823164 0.0034313775
C 2.3890588361 3.3631837879 0.2431774438
C 3.6340362801 2.7762492262 0.1511650166
C 4.9039783569 3.5048297823 0.2037946937
C 5.8960587734 2.5982016171 0.0244221692
C 5.2553630239 1.2914711843 -0.1067767913
H 3.3620373832 -0.5755375548 0.0621747856
H 0.0969438435 -4.4093234806 -0.3356155613
H -3.3690852104 0.6024253322 -0.0477883762
H -0.0873657555 4.3561886635 0.7052435151
H 7.0259176872 -2.6615209693 -0.295772669
H 5.1483033228 -4.4727550127 0.0048984574
H 1.3343182931 -0.6322509567 1.0261758462
H -1.3495990504 -0.6532685855 0.8674849532
H -5.0083771428 -4.5745631987 -0.1708334825
H -6.9404868498 -2.7828558748 -0.1866959541
H -7.0542778617 2.6843627360 -0.081699468
H -5.1564741935 4.4976273277 -0.0207438103
H -1.3266352731 0.7602963763 -1.0803519015
H 1.3620749790 0.7689700436 -0.9327103006
H 5.0339845367 4.5677001252 0.3269930628
H 6.9590898767 2.7827867717 0.0052867218
C 7.4795433779 0.1181804088 -0.6045566581
C 2.4345524662 -4.8811375814 0.1745306575
C -2.2772455664 -4.8661370057 -0.2294407471
C -7.5263299767 -0.1992739321 -0.1101745962
C -2.4225709426 4.8893719460 0.1966306185
C 2.2979033591 4.8306978651 0.6169408133
H 7.6599032277 0.9807897434 -1.252815402
H 8.2159572494 0.1867172668 0.2221730312
H 7.7567918874 -0.7641381311 -1.1870477622
H 1.7537933278 -5.2593655017 0.9448846323
H 2.0852197742 -5.2537617327 -0.7989225844
H 3.4050185162 -5.3365468946 0.3562762618
H -1.7571357893 -5.0899748345 -1.169737183

H -1.7184458781 -5.3329458367 0.5886639211
H -3.2475993750 -5.3472700781 -0.2868671472
H -7.8254642436 -0.7620067512 -1.0025444094
H -7.8500498892 -0.7866305876 0.7571339909
H -8.1411232179 0.7146081759 -0.107643373
H -2.0484708545 5.1477393754 1.1976031970
H -1.7573749921 5.3521221491 -0.5409988146
H -3.3957467715 5.3640168185 0.1006039600
H 1.7403992979 5.3936507788 -0.1395170306
H 1.7797212679 4.9457416201 1.5776169725
H 3.2720891523 5.2946663524 0.7319405865

28H (1.0 nN at 1-5, C_1)

N 3.979052507 -1.491043783 0.103999504
N 0.103547586 -3.499773722 -0.410652843
N -3.768426408 -1.39282573 0.14733248
N -3.92848949 1.4737516 0.068293949
N -0.125110594 3.619600742 -0.234690339
N 3.825896361 1.340874967 0.081689683
C 5.934177506 -0.012183629 0.300561634
C 5.34550227 -1.242480001 0.217500283
C 5.990011663 -2.55116487 0.194231394
C 5.040218152 -3.499873963 0.04223663
C 3.724700299 -2.85892788 -0.011656244
C 2.519255597 -3.489388887 -0.140364983
C 1.238415117 -2.831821739 -0.016533741
C 0.804118502 -1.617967615 0.546604086
C -0.603584336 -1.584880116 0.482561904
C -1.045369661 -2.770178425 -0.122351535
C -2.329081064 -3.364296764 -0.346341707
C -3.546889395 -2.759565956 -0.136247175
C -4.825587233 -3.465088136 -0.103095009
C -5.772460057 -2.553679207 0.227643358
C -5.089699807 -1.2663068 0.351617736
C -5.811538965 -0.038979451 0.574805465
C -5.256262979 1.195268972 0.390292764
C -5.941165306 2.483583778 0.394080665
C -5.058396656 3.444869661 0.050754534
C -3.737293728 2.841349138 -0.151432758
C -2.583818179 3.525575852 -0.422560526
C -1.271717312 2.89110152 -0.473926304
C -0.84867563 1.569415163 -0.704530514
C 0.555962169 1.525024845 -0.581094392
C 1.019492404 2.807956152 -0.273400633
C 2.324458495 3.378368356 -0.052130798
C 3.546658034 2.735577315 0.074217475

C 4.815145853 3.452483 0.236355757<br>C 5.79956147 2.527871547 0.331290599<br>C 5.162511675 1.217040619 0.237407089<br>H 3.329642055 -0.714456623 0.020094463<br>H 0.111149717 -4.398090737 -0.860307755<br>H -3.249243819 0.723528676 0.027111096<br>H -0.130319945 4.594676373 0.011868861<br>H 7.051807934 -2.714212677 0.27781138<br>H 5.208756626 -4.56288515 -0.003776387<br>H 1.433338272 -0.870503902 1.004559351<br>H -1.255003447 -0.81457038 0.860192615<br>H -4.984365955 -4.519462214 -0.26172766<br>H -6.829445731 -2.724465657 0.357088775<br>H -6.98719481 2.624227403 0.611104364<br>H -5.274295519 4.495934191 -0.038564419<br>H -1.475768215 0.737426325 -0.986129675<br>H 1.19014089 0.666921274 -0.725529373<br>H 4.95817095 4.519586398 0.264373076<br>H 6.855708324 2.709860059 0.452315879<br>C 7.446215217 0.114039391 0.446094505<br>C 2.461188841 -4.97552711 -0.41706799<br>C -2.288925474 -4.804949048 -0.814556193<br>C -7.275348704 -0.1704356 0.92869489<br>C -2.653804394 5.035641986 -0.606925769<br>C 2.286377258 4.893956901 0.062151636<br>H 7.692847138 0.678059688 1.353127673<br>H 8.001879117 -0.83910761 0.510030159<br>H 7.868776154 0.668815683 -0.399983095<br>H 1.817927835 -5.487670879 0.308032737<br>H 2.064102873 -5.177956478 -1.421818378<br>H 3.442269267 -5.443438585 -0.376015673<br>H -1.764377222 -4.87535842 -1.775621822<br>H -1.763492705 -5.440925297 -0.093141898<br>H -3.280735351 -5.217516377 -0.971443636<br>H -7.866070994 -0.536045695 0.080119351<br>H -7.410428422 -0.879478977 1.75147845<br>H -7.70454449 0.778682663 1.248289595<br>H -2.276196494 5.572991956 0.274936143<br>H -2.043575834 5.340156216 -1.464723048<br>H -3.679032705 5.406523895 -0.78906448<br>H 1.784971858 5.329918579 -0.808470454<br>H 1.732346257 5.19266146 0.960989557<br>H 3.269472553 5.34415272 0.131974991
**28H** (1.0 nN at 2-5, C_1)<br><br>N 3.937208062 -1.60308435 0.026206672

N 0.164895236 -3.697131083 -0.070721048
N -3.705438835 -1.361520182 0.017901913
N -3.937214355 1.603057117 -0.026106534
N -0.164900777 3.697146685 0.070459805
N 3.705459639 1.361522234 -0.01818787
C 5.804759796 -0.007744619 -0.203885089
C 5.280368022 -1.266800445 -0.121508513
C 6.004097414 -2.537680924 -0.116378265
C 5.117582664 -3.545373242 0.037138247
C 3.768415977 -2.989830764 0.121461408
C 2.602310419 -3.694469662 0.221162131
C 1.313205406 -3.024930291 0.275031839
C 0.91022187 -1.721049888 0.627444101
C -0.488565869 -1.635451657 0.47721649
C -0.960790292 -2.87502813 0.031069333
C -2.262225023 -3.410638115 -0.243145345
C -3.471619988 -2.747677035 -0.188739405
C -4.760822466 -3.432116914 -0.276858469
C -5.723294331 -2.498965281 -0.097676827
C -5.042016517 -1.214339964 0.065673372
C -5.804714439 0.007690351 0.204179144
C -5.280342011 1.266757256 0.121859018
C -6.0040759 2.537635019 0.117022746
C -5.117592622 3.545345799 -0.036560045
C -3.768441089 2.989813792 -0.121219099
C -2.602354449 3.694467772 -0.221038885
C -1.313248374 3.024948473 -0.275173834
C -0.910288577 1.721097211 -0.627725076
C 0.488515151 1.635495686 -0.477635674
C 0.960779452 2.875056463 -0.031488462
C 2.262234236 3.410677189 0.242613626
C 3.47162876 2.747707827 0.188263024
C 4.760828203 3.432157202 0.276358462
C 5.723311473 2.498969642 0.097438134
C 5.042043798 1.214317525 -0.065745814
H 3.222121027 -0.889095081 -0.021309756
H 0.153356441 -4.649792647 -0.391261032
H -3.222122037 0.889058438 0.021171685
H -0.153338204 4.649779203 0.391085721
H 7.073397603 -2.638652521 -0.199899859
H 5.347696958 -4.596584382 0.094008196
H 1.542306304 -0.935899925 1.01267768
H -1.116067928 -0.784804782 0.687499866
H -4.925391451 -4.487563506 -0.416148698
H -6.789470272 -2.657325715 -0.089395368
H -7.073359448 2.638589075 0.200786213
H -5.347716645 4.596564582 -0.09325294

H -1.542401793 0.935960849 -1.012939082<br>H 1.115997608 0.784859197 -0.688019428<br>H 4.925395367 4.487622563 0.415504472<br>H 6.789488393 2.657324812 0.089252156<br>C 7.297944458 0.16353886 -0.387658962<br>C 2.59216322 -5.218318039 0.198888924<br>C -2.248618723 -4.886207478 -0.600785476<br>C -7.297854604 -0.163622244 0.388282515<br>C -2.59221772 5.218313613 -0.198554017<br>C 2.24863236 4.886275112 0.600137233<br>H 7.5095025 0.861282089 -1.203915465<br>H 7.773506602 0.561359214 0.516491267<br>H 7.789682559 -0.776320552 -0.633591521<br>H 1.935448549 -5.599102816 0.99002904<br>H 2.20163329 -5.591679594 -0.759182186<br>H 3.571502892 -5.7104801 0.337258988<br>H -1.626357344 -5.050546704 -1.488755919<br>H -1.836350967 -5.48407128 0.220237706<br>H -3.233399282 -5.274415149 -0.835237845<br>H -7.773670288 -0.56111833 -0.515878596<br>H -7.509211006 -0.861641292 1.2043536<br>H -7.789498835 0.776166932 0.634677706<br>H -2.201544063 5.591546715 0.759508464<br>H -1.935629697 5.599214149 -0.989743386<br>H -3.571584034 5.710485086 -0.336703728<br>H 1.836225998 5.484052946 -0.220877496<br>H 1.626489455 5.050666176 1.48818291<br>H 3.23343086 5.274542807 0.834410537
**28H** (1.0 nN at 2-6, C_1)<br><br>N 3.831540049 -1.524152596 0.231487899<br>N 0.092189886 -3.629159242 -0.24104822<br>N -3.76955585 -1.443437465 -0.077352581<br>N -3.880818747 1.427723919 0.188680194<br>N -0.052922917 3.683904472 -0.051364114<br>N 3.753931448 1.525133193 -0.110548363<br>C 5.768137836 0.042048234 0.081141639<br>C 5.190463355 -1.194453272 0.214054609<br>C 5.891229359 -2.476038698 0.347038826<br>C 4.986547123 -3.476150542 0.411831964<br>C 3.642392128 -2.915186603 0.333751461<br>C 2.47464606 -3.625146687 0.318458151<br>C 1.180205462 -2.968092728 0.276026164<br>C 0.704966463 -1.730147746 0.743841995<br>C -0.684740148 -1.680112662 0.510888139<br>C -1.068863062 -2.868607045 -0.122019084

C -2.32193739 -3.413654604 -0.551100907
C -3.541458114 -2.781628812 -0.472775915
C -4.828993844 -3.439635719 -0.684052303
C -5.790925565 -2.536553974 -0.37593552
C -5.103175985 -1.296390597 -0.016246357
C -5.821925611 -0.092161726 0.314309291
C -5.23127277 1.138518944 0.383799895
C -5.882634837 2.425249833 0.60033827
C -4.955296219 3.40229384 0.509643856
C -3.645205743 2.805156806 0.243077392
C -2.469675014 3.490755255 0.101253852
C -1.177149617 2.891181701 -0.137853121
C -0.71992673 1.601725153 -0.462313124
C 0.686531187 1.64026639 -0.537852328
C 1.106470231 2.952203352 -0.278553474
C 2.386503896 3.607820198 -0.27515756
C 3.585184449 2.933784312 -0.234358394
C 4.896374026 3.566454594 -0.271290041
C 5.817228658 2.577294652 -0.172790559
C 5.075985615 1.31072785 -0.066838225
H 3.120802331 -0.816681613 0.101266463
H 0.143765918 -4.532481609 -0.678916088
H -3.220061016 0.673077608 0.050760776
H -0.06952201 4.651958163 0.216731695
H 6.960952167 -2.593379334 0.38210281
H 5.194445326 -4.529549547 0.510195794
H 1.286323302 -0.982516086 1.261151679
H -1.368076059 -0.900795216 0.800457932
H -4.989034673 -4.462664148 -0.983482844
H -6.85915993 -2.682818194 -0.401670609
H -6.935573062 2.560340812 0.784073294
H -5.141091216 4.457658846 0.615627294
H -1.328557097 0.738776215 -0.678365008
H 1.348725857 0.826849154 -0.780367548
H 5.08675706 4.624247234 -0.363865166
H 6.889254146 2.689245739 -0.165111829
C 7.280798174 0.149877221 0.08198563
C 2.421470484 -5.151781113 0.25675597
C -2.248740755 -4.827366095 -1.090466706
C -7.312922531 -0.234833527 0.520580285
C -2.459075747 4.999876297 0.230303318
C 2.30450784 5.135281776 -0.276571869
H 7.640895096 0.60470538 -0.847175882
H 7.628382292 0.777243733 0.910041972
H 7.770020696 -0.816185356 0.179752028
H 1.564192391 -5.503013508 0.841501308
H 2.271431226 -5.489837611 -0.779332169

H 3.285959155 -5.720339356 0.63343917
H -1.569753891 -4.87011763 -1.950645647
H -1.875740202 -5.521134354 -0.327947682
H -3.212272179 -5.19187384 -1.432773144
H -7.828159009 -0.47017724 -0.418407259
H -7.53043155 -1.044005838 1.224885734
H -7.759590926 0.673605269 0.922860218
H -1.942488503 5.322742864 1.14467745
H -1.949417982 5.457830036 -0.625865629
H -3.459274489 5.423712073 0.265796422
H 1.56943753 5.454500144 -1.023250859
H 1.949651326 5.479585991 0.70474735
H 3.217159216 5.708895659 -0.485612736

28H (1.0 nN at 3-5, C_1)

N 3.88081866 -1.427733574 -0.188650432
N 0.05291659 -3.683902988 0.051393345
N -3.753935088 -1.525125405 0.110539521
N -3.831534855 1.524159752 -0.231512435
N -0.092183425 3.629156521 0.241035768
N 3.769559471 1.443425973 0.077359962
C 5.821928707 0.092145642 -0.314291611
C 5.231273663 -1.138534438 -0.383771447
C 5.882632973 -2.425269634 -0.600293492
C 4.955291579 -3.402310046 -0.509589142
C 3.645201994 -2.805166102 -0.243033058
C 2.469669475 -3.490760268 -0.101205304
C 1.177143207 -2.891181514 0.137884665
C 0.719919011 -1.601719385 0.462323061
C -0.686538967 -1.640259841 0.537856577
C -1.106477995 -2.95219955 0.278569841
C -2.386512389 -3.607814225 0.275165309
C -3.585191933 -2.933776576 0.234351331
C -4.896383206 -3.566444144 0.271264844
C -5.817235369 -2.577282243 0.172757128
C -5.075988582 -1.310717151 0.066818964
C -5.768136931 -0.042035491 -0.081160606
C -5.190459033 1.194463872 -0.214078835
C -5.891221806 2.476050458 -0.347068217
C -4.986536978 3.476159762 -0.411864946
C -3.642383362 2.915193075 -0.333781964
C -2.474635503 3.625150529 -0.318486638
C -1.18019662 2.968093967 -0.276048136
C -0.704957733 1.73014926 -0.743864717
C 0.684746925 1.680109478 -0.510900992
C 1.068868954 2.868603118 0.122008906

C 2.321943599 3.413649982 0.551090767<br>C 3.541463496 2.781621923 0.472768668<br>C 4.828999836 3.439631028 0.684034117<br>C 5.79093045 2.536545739 0.375924518<br>C 5.103179562 1.29637929 0.016249118<br>H 3.220063345 -0.673084157 -0.05073566<br>H 0.069516928 -4.651959708 -0.216691671<br>H -3.120798643 0.816688781 -0.101283672<br>H -0.143760559 4.532477704 0.678906166<br>H 6.935571003 -2.560365585 -0.784026048<br>H 5.141083451 -4.457676929 -0.615559826<br>H 1.328548208 -0.738767124 0.678365576<br>H -1.348734872 -0.826840261 0.780360415<br>H -5.086769906 -4.624236696 0.363832626<br>H -6.889261064 -2.68923169 0.165063909<br>H -6.96094428 2.59339388 -0.382133462<br>H -5.194432684 4.529558987 -0.510232098<br>H -1.286313239 0.982521383 -1.261181354<br>H 1.368083027 0.900790464 -0.80046636<br>H 4.989041538 4.462663371 0.983450919<br>H 6.859164808 2.682811177 0.401652089<br>C 7.312925875 0.234814061 -0.520561798<br>C 2.459069383 -4.999883146 -0.230232725<br>C -2.304518796 -5.13527625 0.276580306<br>C -7.28079762 -0.149860829 -0.082001777<br>C -2.421457422 5.151784488 -0.256780398<br>C 2.24874975 4.827363226 1.090452687<br>H 7.530435893 1.043959319 -1.22489827<br>H 7.828156589 0.470196504 0.418419041<br>H 7.759598941 -0.673639676 -0.92280257<br>H 1.949403125 -5.457823492 0.625938218<br>H 1.942491229 -5.322763286 -1.144607301<br>H 3.459268511 -5.423719929 -0.265709508<br>H -1.949652291 -5.479581758 -0.704734656<br>H -1.569456932 -5.454495258 1.023267574<br>H -3.217172328 -5.708889532 0.48561344<br>H -7.628386961 -0.777211538 -0.910068235<br>H -7.64089102 -0.604705239 0.847152845<br>H -7.770018044 0.816204863 -0.179746939<br>H -2.271438643 5.489839074 0.779310924<br>H -1.564165905 5.503015071 -0.841507354<br>H -3.285936265 5.720345573 -0.633482539<br>H 1.875756027 5.521132078 0.327930862<br>H 1.569758432 4.870118464 1.950627967<br>H 3.212280799 5.191867798 1.432763546
**28H** (1.0 nN at 3-6, C_1)

N 3.816873078 -1.349889574 0.008416664
N -0.047284381 -3.713690167 -0.129942318
N -3.868888533 -1.636429393 0.002393972
N -3.816861207 1.349877563 -0.008319587
N 0.047232491 3.713788633 0.128476828
N 3.868898805 1.636361644 -0.001576218
C 5.83766984 0.120074889 0.00747004
C 5.193786005 -1.088878986 -0.01165024
C 5.833080633 -2.398720545 -0.072018064
C 4.88231937 -3.352796493 -0.106328492
C 3.557825203 -2.730668565 -0.053830869
C 2.376532675 -3.428919468 -0.069642187
C 1.042646938 -2.886993493 0.075349874
C 0.519571346 -1.643311745 0.465485543
C -0.885352574 -1.742709845 0.477287738
C -1.244019718 -3.04402599 0.095649731
C -2.512010011 -3.71493164 -0.032251565
C -3.711689844 -3.044545105 -0.037235906
C -5.028983812 -3.668331183 -0.094471291
C -5.941587245 -2.666656934 -0.082006561
C -5.187243609 -1.408232588 -0.025656134
C -5.837648141 -0.120128276 -0.005271056
C -5.193754989 1.088833974 0.01289553
C -5.833049295 2.398692908 0.072943652
C -4.882303098 3.352828383 0.105792939
C -3.557817903 2.730714103 0.052798528
C -2.376558279 3.429033955 0.067584113
C -1.04263726 2.886999948 -0.076767565
C -0.519452656 1.643098726 -0.466031761
C 0.885480503 1.742469175 -0.477378544
C 1.244041284 3.04397312 -0.096291439
C 2.512000095 3.714885378 0.031899728
C 3.711671381 3.04448444 0.037669513
C 5.028930837 3.668283412 0.095596844
C 5.941543095 2.666608774 0.083969132
C 5.187240073 1.40817241 0.02737612
H 3.153896859 -0.586386918 0.026303654
H 0.016993739 -4.664725796 -0.448760616
H -3.153861133 0.586401003 -0.026431254
H -0.017156391 4.664945427 0.446916992
H 6.896074701 -2.567751378 -0.090761698
H 5.065892976 -4.412144057 -0.149286639
H 1.087814577 -0.776331696 0.763482423
H -1.590836616 -0.983708947 0.765786577
H -5.234261528 -4.726399817 -0.129410422
H -7.014991823 -2.763322668 -0.113314578

H -6.896033377 2.567698769 0.092419831<br>H -5.065887832 4.41219179 0.148288165<br>H -1.087607581 0.77597341 -0.763782971<br>H 1.5910496 0.983323561 -0.76526896<br>H 5.234187718 4.726358822 0.130444222<br>H 7.0149271 2.763285387 0.115931227<br>C 7.351583571 0.167213905 0.000363782<br>C 2.404946715 -4.93549114 -0.249406847<br>C -2.464511494 -5.23154233 -0.181952433<br>C -7.351555882 -0.16728347 0.003238143<br>C -2.405002256 4.93581234 0.245682694<br>C 2.464465885 5.231550855 0.18105584<br>H 7.721694951 0.723061752 -0.868044747<br>H 7.729938392 0.670812433 0.896881209<br>H 7.803396458 -0.821516195 -0.029531816<br>H 1.855234266 -5.434569859 0.55743039<br>H 1.944283766 -5.226213721 -1.203608603<br>H 3.409402379 -5.346748776 -0.255663796<br>H -2.010077771 -5.495063235 -1.14665823<br>H -1.836423914 -5.662164806 0.606345656<br>H -3.435128213 -5.753253595 -0.142553546<br>H -7.730810444 -0.670362288 -0.893196682<br>H -7.720778715 -0.723649558 0.871686789<br>H -7.803356215 0.821418295 0.034224441<br>H -1.945355464 5.227628192 1.200046014<br>H -1.854351621 5.433852077 -0.561146755<br>H -3.40941032 5.347209719 0.250232234<br>H 1.836741881 5.661900665 -0.607681143<br>H 2.009602548 5.495428944 1.145460529<br>H 3.435107182 5.753232437 0.141893562
**$28M_{1a}$** (1.0 nN at 1-4, C_1)<br><br>N -3.5462535696 1.6655739855 0.4549484823<br>N 0.5622553706 3.3185505856 -0.3814320995<br>N 4.0049603325 0.6543003092 -0.2773509956<br>N 3.2975073141 -1.5508218784 0.8409034336<br>N -0.9437197719 -2.0696049166 0.0800333826<br>N -3.8023349847 -0.8736576668 -0.3028804478<br>C -5.6498444432 0.3814062439 0.7440815803<br>C -4.8463153756 1.5402119908 0.9402436787<br>C -5.1763100121 2.7853911072 1.5155862268<br>C -4.0865859491 3.6371952216 1.3515445436<br>C -3.0580643030 2.9252858164 0.6772324739<br>C -1.7683536443 3.4425968497 0.2872109059<br>C -0.6570665591 2.7075291116 -0.0585206902<br>C -0.3769572233 1.2774893351 -0.126708432

C 0.9402248734 1.0939917490 -0.4256469173
C 1.5889725445 2.3872255125 -0.5831668551
C 2.8812451895 2.7621206801 -0.8811724928
C 4.0285355171 1.8851896657 -0.8045485897
C 5.4087496216 2.2463816223 -1.1386115174
C 6.2012828558 1.1975333432 -0.7682172193
C 5.3296855899 0.1856468599 -0.2010676831
C 5.6662408478 -0.9933958758 0.4532460730
C 4.6443364356 -1.8174543280 1.0296233955
C 4.7049521788 -2.9058283365 1.9278380816
C 3.3914232769 -3.2459191104 2.2842855126
C 2.5155798869 -2.3922011758 1.5757826139
C 1.0602747933 -2.3363967880 1.5174730951
C 0.4289705369 -2.2423359105 0.3065400201
C 0.9999820034 -2.4982338869 -1.0163025162
C -0.0005207418 -2.5611464671 -1.9253318484
C -1.2781765266 -2.3002286745 -1.2638675445
C -2.5327330177 -2.3638839083 -1.8036979751
C -3.7344224380 -1.9502962606 -1.1026334929
C -5.0435082110 -2.5739928263 -1.2288638054
C -5.9058809286 -1.8479564665 -0.4547097226
C -5.1484120193 -0.7428756651 0.0972346007
H -3.1295991756 0.8661752257 -0.029933174
H 0.7240239559 4.3071967533 -0.3065268177
H 3.0514768767 -0.6698584681 0.3597154549
H -1.6353819162 -1.8322482311 0.7739607951
H -6.1143333835 3.0284329296 1.9891693310
H -4.0193258809 4.6551946237 1.6972540047
H -1.0998331863 0.4885646432 0.0189426260
H 1.4343708231 0.1513931258 -0.5862423625
H 5.7403847067 3.1704368932 -1.584606908
H 7.2746018488 1.1298487832 -0.8638907503
H 5.6075018834 -3.3820381055 2.2788604167
H 3.0883790357 -4.0482135908 2.9382008967
H 2.0527155551 -2.6610141505 -1.1876232381
H 0.1080729851 -2.7652342054 -2.9781238266
H -5.2679042383 -3.4600669787 -1.8023330504
H -6.9614813703 -2.0264098586 -0.3135417289
C -7.0868426832 0.3990570828 1.2495632696
C -1.6142850044 4.9501984307 0.2922519908
C 3.1258962417 4.2272860822 -1.1696998088
C 7.1001463290 -1.4381520160 0.6933691659
C 0.2833577795 -2.3897004126 2.8066407151
C -2.7407624595 -2.8593341330 -3.2164885124
H -7.1301174274 0.9364583694 2.2010213928
H -7.8452145077 0.8635212074 0.5887182574
H -7.4189342568 -0.6228365068 1.4422322429

H -0.9850386682 5.2962390478 1.1226617845<br>H -1.1552915011 5.2925807378 -0.6433245468<br>H -2.5776015086 5.4517746588 0.3675379382<br>H 3.2088470228 4.8172552743 -0.2464852289<br>H 4.0483388147 4.3769338994 -1.7298202872<br>H 2.3128581166 4.6496346236 -1.7701498358<br>H 7.3306737376 -1.3410251310 1.7610706418<br>H 7.1952499103 -2.5003500389 0.4455603515<br>H 7.8947618349 -0.9058238202 0.1457751235<br>H 0.1079584798 -1.3851179988 3.2123866615<br>H -0.6882694364 -2.8757021054 2.6710992272<br>H 0.8396837867 -2.9471186738 3.5650747084<br>H -3.4931426963 -3.6549560676 -3.2365082606<br>H -3.1036622155 -2.0584445651 -3.8699428987<br>H -1.8279395975 -3.2689585914 -3.6491683398
**28M$_{1a}$** (1.0 nN at 1-5, C_1)<br><br>N -3.95099943 2.10135097 -0.157113606<br>N 0.256026888 3.431519388 -0.333997115<br>N 3.773238899 0.565312559 0.035194267<br>N 3.532432873 -2.060854032 1.026923282<br>N -0.606550789 -2.939207009 0.797427871<br>N -3.953305626 -0.450502517 -0.161464711<br>C -6.018337628 0.811868704 0.165236943<br>C -5.329114492 2.042765554 -0.002346057<br>C -5.778899783 3.381570152 0.04059674<br>C -4.663817935 4.209691995 -0.07695312<br>C -3.509096062 3.382916079 -0.198230549<br>C -2.124668228 3.772005029 -0.301748508<br>C -1.042102952 2.922531921 -0.270245902<br>C -0.85171329 1.467431309 -0.185213992<br>C 0.480885003 1.186478651 -0.217882453<br>C 1.235701559 2.432240494 -0.301674851<br>C 2.566554939 2.74242225 -0.367064378<br>C 3.69416843 1.826014617 -0.414051132<br>C 4.981046778 2.260564785 -0.963892634<br>C 5.85194525 1.232310296 -0.804366682<br>C 5.119195643 0.157642882 -0.158036974<br>C 5.657346288 -1.037638264 0.273212124<br>C 4.905930563 -2.088370974 0.874581642<br>C 5.34049618 -3.334477647 1.359042208<br>C 4.212578701 -4.044174254 1.793185299<br>C 3.081370352 -3.243243592 1.553929246<br>C 1.666443667 -3.531170492 1.754969993<br>C 0.795690617 -3.07874908 0.795029519<br>C 1.204221495 -2.718252243 -0.56873941

C 0.116431861 -2.419865127 -1.30057123
C -1.083758912 -2.470918774 -0.455403633
C -2.29676523 -2.021798293 -0.873761733
C -3.556061493 -1.723231308 -0.143019449
C -4.655428557 -2.573986288 0.268932367
C -5.74951703 -1.753712668 0.45240103
C -5.319808846 -0.400658813 0.180733995
H -3.462625112 1.196505351 -0.259887258
H 0.462195361 4.4090682 -0.423768174
H 3.004131585 -1.21572523 0.811723409
H -1.179597332 -3.002585096 1.621364999
H -6.802454561 3.699910486 0.162809423
H -4.674401166 5.286364225 -0.055113287
H -1.633991147 0.725644334 -0.102751214
H 0.940733598 0.210655659 -0.18218188
H 5.178788245 3.209508916 -1.435991507
H 6.885426216 1.212546727 -1.111173788
H 6.361656877 -3.681256787 1.373533091
H 4.197942937 -5.047091457 2.188000866
H 2.221711278 -2.771400631 -0.91887789
H 0.110565205 -2.155825858 -2.344344516
H -4.619159483 -3.648428164 0.364778582
H -6.742278402 -2.059634692 0.748653998
C -7.519268885 0.86968963 0.383244298
C -1.829228188 5.251498693 -0.420984818
C 2.902794333 4.222844526 -0.424218507
C 7.13343552 -1.319186953 0.126874319
C 1.338832951 -4.341888468 2.988574033
C -2.425666766 -1.549398982 -2.320928602
H -7.733648064 1.084774991 1.436774896
H -8.032405246 1.648558238 -0.215174829
H -7.976820277 -0.090896727 0.140598586
H -1.278887018 5.624149218 0.45297699
H -1.225604603 5.461983112 -1.313261772
H -2.73942252 5.840857781 -0.510615547
H 2.340703049 4.774321712 0.339221119
H 3.95593914 4.403336248 -0.217604169
H 2.667169473 4.666361395 -1.401514888
H 7.29174408 -2.22318112 -0.469329804
H 7.670143682 -0.498950247 -0.34260019
H 7.579349916 -1.493986759 1.11138772
H 1.206615788 -3.711425139 3.876697513
H 0.429538713 -4.934734307 2.852660774
H 2.178793431 -5.032601781 3.216768818
H -3.467601949 -1.629751784 -2.636779439
H -2.141160879 -0.493923874 -2.417373135
H -1.821691422 -2.139563056 -3.012051944

$28M_{1a}$ (1.0 nN at 2-5, C_1) An undesired dihedral angle inverted in the geometry optimization of this structure, see Figure S...

N -3.839916403 2.317529626 -0.083616062
N 0.073046359 3.409279231 -1.16603341
N 3.379366089 0.438642712 0.090778521
N 3.179027598 -2.385829213 0.91353465
N -0.645343073 -3.299106126 -0.149089507
N -3.669932501 -0.250822342 -0.340356528
C -5.435618046 0.780388832 0.973885548
C -5.01505441 2.099520844 0.612172929
C -5.51197473 3.378469789 0.94562836
C -4.609361763 4.335021611 0.45314904
C -3.542536164 3.642581797 -0.164151738
C -2.267407607 4.078190449 -0.718174745
C -1.175725559 3.258139703 -0.550084103
C -0.989698552 2.108303381 0.357322297
C 0.289096628 1.668031363 0.279838925
C 1.013076386 2.467363972 -0.699367664
C 2.315809515 2.431958837 -1.098023208
C 3.391297016 1.651805166 -0.480989251
C 4.720779863 2.261379024 -0.385384371
C 5.510696236 1.393661946 0.293123855
C 4.698830741 0.22160739 0.587989435
C 5.178917762 -0.927879393 1.183791244
C 4.472663605 -2.161860467 1.339909418
C 4.925972605 -3.371499141 1.898105993
C 3.889819618 -4.310935943 1.80024315
C 2.799683353 -3.688728064 1.170465412
C 1.453763729 -4.173443412 0.925283502
C 0.738201937 -3.518294565 -0.047373043
C 1.320115371 -2.853113549 -1.212935592
C 0.338695131 -2.259453987 -1.923641465
C -0.925196295 -2.403167557 -1.206804747
C -2.010357075 -1.600168772 -1.39727209
C -3.127858431 -1.460612384 -0.479245081
C -3.833631409 -2.42216894 0.358242838
C -4.840532166 -1.733214912 0.988198178
C -4.732312914 -0.346117704 0.569834199
H -3.357328202 1.502574137 -0.490634966
H 0.279122117 4.119019947 -1.846175034
H 2.619724637 -1.633754602 0.518709093
H -1.27381338 -3.431245183 0.626773897
H -6.414047941 3.578301594 1.50324832
H -4.676561472 5.404563073 0.573016637
H -1.755882576 1.727658303 1.012096814

H 0.752403864 0.864685829 0.83117119
H 4.988911017 3.23944641 -0.753270463
H 6.549538676 1.536338015 0.542912789
H 5.905023317 -3.543004479 2.31559875
H 3.915872953 -5.338069714 2.126811892
H 2.366239098 -2.911991068 -1.467926614
H 0.453922786 -1.7221877 -2.849852958
H -3.640987794 -3.484447839 0.407818202
H -5.569226264 -2.141310689 1.672639525
C -6.631194232 0.665052888 1.879785043
C -2.180117253 5.376805568 -1.5024767
C 2.73944329 3.424628935 -2.164358318
C 6.599376117 -0.970378196 1.704888965
C 0.918626846 -5.272349617 1.818770981
C -1.968501395 -0.561014715 -2.499531201
H -6.419347134 1.105973637 2.859507014
H -7.482565022 1.206181338 1.455351593
H -6.927499433 -0.373636513 2.02316676
H -1.208855068 5.858705593 -1.340558689
H -2.280416948 5.190575712 -2.580515211
H -2.95443392 6.127184301 -1.250625517
H 2.838887626 4.446147077 -1.771022251
H 3.694670731 3.142925561 -2.608360435
H 2.006357955 3.450839142 -2.978868112
H 7.215541802 -1.633646375 1.088517
H 7.064769797 0.01097141 1.727146862
H 6.609901265 -1.365318591 2.724411277
H 0.858896432 -4.957076793 2.867134088
H -0.078839686 -5.581001605 1.492955424
H 1.559387631 -6.183263031 1.799664558
H -2.984020457 -0.304327781 -2.804005351
H -1.490032471 0.363643594 -2.150200095
H -1.425291693 -0.916688183 -3.376934977

28M$_{1a}$ (1.0 nN at 2-6, C_1) An undesired dihedral angle inverted in the geometry optimization of this structure, see Figure S…

N -3.177168885 1.783841785 0.257368343
N 0.793196591 3.89168979 -0.070577876
N 4.12177816 1.221262683 0.022731047
N 3.198641716 -1.158379422 0.700252437
N -1.253590222 -2.652154056 -0.145464426
N -3.741814862 -1.516399375 -0.467648358
C -5.283705537 0.325928511 0.324857607
C -4.428656561 1.413712761 0.754636966
C -4.813697773 2.443524605 1.626185478
C -3.812818913 3.421836132 1.638621754

C -2.791863612 3.019469447 0.770697525
C -1.568190426 3.756187506 0.511420927
C -0.418044317 3.187465919 0.034002712
C -0.07333567 1.816901963 -0.337344622
C 1.262795595 1.731265587 -0.55915027
C 1.868572461 3.040995909 -0.346681559
C 3.174144583 3.449181336 -0.349067673
C 4.271479604 2.505171334 -0.310507634
C 5.686323726 2.805744379 -0.530090389
C 6.376946776 1.648855455 -0.316071611
C 5.399846808 0.632134234 0.039136948
C 5.599741289 -0.692842248 0.370085205
C 4.51614434 -1.578371821 0.675392414
C 4.49930096 -2.965061115 0.898264565
C 3.154825524 -3.361111837 1.028680224
C 2.354545463 -2.214298687 0.886129172
C 0.90431289 -2.010925115 0.853195855
C 0.120558125 -2.798771643 0.069590739
C 0.504500509 -3.981441524 -0.710781163
C -0.602929255 -4.511371496 -1.279394569
C -1.747563432 -3.659218121 -0.959301855
C -3.047416688 -3.75141221 -1.383501177
C -3.99176849 -2.692224962 -1.095162356
C -5.409174498 -2.801056011 -1.438708927
C -6.022313009 -1.700511291 -0.946199735
C -4.992398438 -0.861631959 -0.321466126
H -2.715085279 1.266674653 -0.473038379
H 0.911848107 4.839954084 0.238902057
H 2.997981115 -0.166641753 0.53182722
H -1.872841333 -1.900134741 0.147414566
H -5.72712262 2.474636598 2.195033636
H -3.805433079 4.323774504 2.227563494
H -0.759775586 0.988302087 -0.400687263
H 1.815865285 0.859747919 -0.867372638
H 6.098698815 3.757937333 -0.824902116
H 7.444153946 1.511350347 -0.400075125
H 5.365971695 -3.605213725 0.95247917
H 2.786563996 -4.353847779 1.232039634
H 1.517930302 -4.339924239 -0.790475749
H -0.652342918 -5.38553781 -1.908652881
H -5.865081943 -3.611957735 -1.983942066
H -7.066634492 -1.457978258 -1.052989863
C -6.738823883 0.604420306 0.671251699
C -1.462065402 5.228832192 0.909250748
C 3.466796315 4.925175362 -0.228701176
C 6.979590862 -1.293357706 0.395729743
C 0.347868859 -0.898357149 1.703306354

C -3.552790353 -4.966420956 -2.153157519
H -6.840276534 0.693998106 1.758021989
H -7.078277026 1.546277909 0.231862216
H -7.403971531 -0.195996059 0.365109642
H -0.76395874 5.341425315 1.750579493
H -1.050453873 5.800460065 0.067213261
H -2.380777544 5.755881616 1.201060728
H 3.342171287 5.278231325 0.804782279
H 4.491459388 5.15649902 -0.520408659
H 2.801262745 5.515139663 -0.869114989
H 7.055413843 -2.104606425 -0.335675377
H 7.7502724 -0.557648841 0.172008727
H 7.18856285 -1.721318229 1.381365602
H 0.884407036 0.043565313 1.553675671
H -0.710370156 -0.709317069 1.507176514
H 0.44761832 -1.157974618 2.764042409
H -4.476240486 -5.335072579 -1.693471033
H -3.795390931 -4.708525684 -3.191424703
H -2.873869926 -5.83747021 -2.18600222

28M$_{1a}$ (1.0 nN at 3-5, C_1)

N -3.304674641 1.602610336 0.548357706
N 0.559803085 3.426210716 -0.781006833
N 3.819307183 0.778719323 -0.362065085
N 2.981420214 -1.484766728 1.044241263
N -1.063026844 -2.23998466 0.059219946
N -3.643526175 -0.953820499 -0.266655636
C -5.323204025 0.251781877 1.037961218
C -4.509110845 1.397710148 1.214035332
C -4.808302299 2.609240721 1.877224813
C -3.803976604 3.522735922 1.577144123
C -2.857447532 2.883811739 0.73067742
C -1.676345718 3.47459751 0.16550268
C -0.599811967 2.778818401 -0.333397036
C -0.297620759 1.356349824 -0.432937114
C 0.993936726 1.209369552 -0.836419039
C 1.596616666 2.519497126 -1.026852348
C 2.898682023 2.864114916 -1.318079635
C 3.965016569 1.924159815 -1.061862831
C 5.374481136 2.162332351 -1.3491226
C 6.075170394 1.138511879 -0.781464097
C 5.101023967 0.26662154 -0.138725352
C 5.353251891 -0.823923247 0.685072757
C 4.337996569 -1.611547667 1.297661784
C 4.465486991 -2.648463806 2.250703276
C 3.186937346 -3.116774554 2.561554845

C	2.257484047	-2.40372325	1.771573543
C	0.833150148	-2.580432674	1.625671254
C	0.282705557	-2.369798494	0.376875145
C	0.968337907	-2.463999166	-0.91308748
C	0.041373567	-2.486139014	-1.905134645
C	-1.280734475	-2.3237524	-1.32311565
C	-2.505841548	-2.318568474	-1.934961017
C	-3.683394223	-1.925729774	-1.199550911
C	-5.027379255	-2.456241059	-1.324120576
C	-5.79668343	-1.799816354	-0.398937809
C	-4.940328475	-0.817333465	0.235787537
H	-2.93157668	0.848658591	-0.027935982
H	0.709445785	4.414943303	-0.679490265
H	2.639792137	-0.69407722	0.499619702
H	-1.811470429	-2.010061703	0.695354659
H	-5.675177717	2.787142702	2.493717676
H	-3.733008373	4.53597773	1.936326
H	-0.980619431	0.545183982	-0.227111112
H	1.508232948	0.287104356	-1.043072598
H	5.773331379	2.998322508	-1.90309205
H	7.145806292	1.005321595	-0.799958417
H	5.397306162	-3.011935903	2.654764757
H	2.936378978	-3.92385959	3.231489219
H	2.034478188	-2.588103512	-1.018278702
H	0.23364773	-2.598218602	-2.960390339
H	-5.337596874	-3.243588099	-1.994018995
H	-6.850724739	-1.94287596	-0.213854693
C	-6.703402099	0.289360804	1.64469133
C	-1.587740269	4.985851171	0.180747196
C	3.232834583	4.310445438	-1.664231487
C	6.772555872	-1.21410764	1.021304404
C	0.00760649	-3.049478599	2.812989023
C	-2.667057265	-2.681453776	-3.388429415
H	-6.65166479	0.603686274	2.691219868
H	-7.347859577	1.00144688	1.118282026
H	-7.174209657	-0.692916001	1.615623181
H	-0.902298004	5.351950999	0.956468846
H	-1.232769969	5.360233465	-0.786806899
H	-2.563332265	5.439553936	0.352280416
H	2.337376008	4.826090032	-2.025696621
H	3.597683463	4.848612523	-0.779441766
H	3.995505368	4.460702093	-2.451743792
H	6.934502551	-2.280116471	0.836239285
H	7.499075697	-0.652838337	0.437072199
H	6.978840178	-1.029416262	2.081318664
H	0.47933236	-2.73573354	3.747694124
H	-0.993328236	-2.606254852	2.793380525

H -0.140844878 -4.1510773 2.873789828
H -3.483045204 -3.401844329 -3.512382999
H -2.918844561 -1.805260126 -3.996447202
H -1.763876267 -3.13678038 -3.79802387

28M$_{1a}$ (1.0 nN at 3-6, C_1)

N -3.079004298 1.296101795 0.313752494
N 1.050399434 3.812796245 1.269584103
N 4.211851439 1.730903152 0.119992133
N 2.973338157 -0.724506335 -0.217058096
N -1.522613469 -2.68538028 0.202495601
N -3.994734018 -1.671573065 0.033903709
C -5.167439816 0.363867711 -0.750304035
C -4.08757424 1.306046993 -0.644259814
C -3.912680861 2.474998802 -1.395562481
C -2.790888309 3.153293355 -0.894109141
C -2.291941568 2.426823363 0.19210642
C -1.231067343 2.847723658 1.105083303
C -0.03375444 3.237584801 0.577763415
C 0.50111459 2.885069321 -0.735576295
C 1.845378886 3.037507484 -0.711078004
C 2.245556841 3.630251676 0.56282867
C 3.539114225 3.914752347 0.945078723
C 4.538065862 2.998595621 0.438221727
C 5.973245675 3.201050279 0.291972393
C 6.495544318 2.022140165 -0.161312276
C 5.385631225 1.081765763 -0.267356263
C 5.403706254 -0.257696389 -0.619067046
C 4.250163165 -1.100202085 -0.612329496
C 4.166359403 -2.457444898 -0.988369737
C 2.848027875 -2.875628316 -0.819027454
C 2.096064676 -1.784622884 -0.318027917
C 0.685908526 -1.698486359 -0.024727169
C -0.134321012 -2.796446942 0.12824093
C 0.135504234 -4.22231072 0.29440949
C -1.046474419 -4.885054116 0.42672854
C -2.137420222 -3.921420839 0.348221061
C -3.506886618 -4.09778725 0.361634857
C -4.378930068 -2.961045619 0.191532083
C -5.830340293 -3.078036581 0.030889554
C -6.29564302 -1.849206499 -0.303441458
C -5.14864813 -0.945526662 -0.32486834
H -2.941672965 0.523239405 0.950151681
H 1.013609994 4.094678591 2.234535553
H 2.817478234 0.234710773 0.096493468
H -2.103758006 -1.858568791 0.056619024

H -4.542270529 2.792138626 -2.210984132
H -2.398847389 4.10404427 -1.218652232
H -0.103091162 2.490190978 -1.536793801
H 2.548377154 2.809679075 -1.493930031
H 6.501586759 4.123289843 0.481999819
H 7.531418384 1.824638887 -0.391876029
H 4.982209441 -3.052326084 -1.367303637
H 2.444927962 -3.8363831 -1.08998883
H 1.121674141 -4.651437136 0.360298481
H -1.177964559 -5.944013058 0.585906506
H -6.408108448 -3.980851433 0.147070666
H -7.32353683 -1.582082096 -0.490230126
C -6.392765659 0.914682116 -1.445113538
C -1.523713158 2.929883127 2.583394306
C 3.920753956 4.97744811 1.957838733
C 6.695951387 -0.91841965 -1.032324301
C 0.034227919 -0.341345843 0.077575032
C -4.202721848 -5.450740454 0.460011402
H -6.714925026 1.852970757 -0.985683176
H -6.159494461 1.127448765 -2.494914587
H -7.223269355 0.212847677 -1.43822464
H -2.262585228 2.17728845 2.872854932
H -0.622979962 2.756475597 3.181783843
H -1.938694272 3.906336243 2.864824718
H 3.040536956 5.557726719 2.252446982
H 4.35981835 4.540774743 2.862392776
H 4.661670419 5.713147265 1.571883957
H 6.616006409 -1.314593937 -2.049937331
H 7.531191846 -0.220980935 -1.007873889
H 6.931214179 -1.757647486 -0.370454439
H -0.71027859 -0.192070156 -0.717909063
H -0.489302576 -0.229355977 1.035725941
H 0.751369398 0.47369541 -0.005566204
H -4.782034718 -5.511915707 1.38938167
H -4.916174972 -5.551549235 -0.364772219
H -3.586387758 -6.358982446 0.417113204

28M$_{1a}$ (1.0 nN at 5-6, C_1)

N -3.3595961 1.571585472 0.582460621
N 0.568398271 3.316176913 -0.518259561
N 3.856156918 0.633259574 -0.401243919
N 3.061965377 -1.427669694 0.993150634
N -1.059346838 -2.003439229 0.043318054
N -3.654662153 -0.931602928 -0.404869981
C -5.420675049 0.229997962 0.834327682
C -4.599096716 1.341387555 1.164718475

C -4.90500332 2.493418501 1.918830417<br>C -3.86038728 3.402906531 1.757090764<br>C -2.890671558 2.817073698 0.904810224<br>C -1.66799617 3.405525623 0.428677824<br>C -0.610347329 2.697361275 -0.086228606<br>C -0.353905969 1.269029769 -0.226568915<br>C 0.926380172 1.087254644 -0.649346736<br>C 1.564742821 2.382208499 -0.819139595<br>C 2.84690519 2.724400766 -1.173911556<br>C 3.943250273 1.795240869 -1.063344942<br>C 5.328902363 2.03600905 -1.461599368<br>C 6.06719114 0.988268156 -0.990113226<br>C 5.147078317 0.102937169 -0.294436797<br>C 5.428393437 -0.996074853 0.504560698<br>C 4.400537949 -1.702305398 1.19489909<br>C 4.454757843 -2.696068839 2.19702129<br>C 3.139950396 -2.976950387 2.594734616<br>C 2.274363445 -2.187272332 1.80575314<br>C 0.822233832 -2.136146283 1.713851476<br>C 0.267918771 -2.194428632 0.452268369<br>C 0.968277404 -2.610761453 -0.769327399<br>C 0.0853498 -2.689984448 -1.793627771<br>C -1.237226745 -2.300350084 -1.323202831<br>C -2.426083583 -2.303694342 -2.021074674<br>C -3.636994788 -1.851007904 -1.389621567<br>C -4.982426087 -2.326740232 -1.666518809<br>C -5.813061856 -1.700201442 -0.778792188<br>C -4.991500246 -0.782565659 -0.009911434<br>H -2.973120899 0.869333039 -0.046033305<br>H 0.746638618 4.30070904 -0.422293178<br>H 2.8119424 -0.608736166 0.423776835<br>H -1.829364146 -1.656737354 0.599737346<br>H -5.799105615 2.639599836 2.504026583<br>H -3.784946895 4.376714394 2.212060134<br>H -1.057952995 0.475597354 -0.033278211<br>H 1.40039758 0.149757387 -0.886763073<br>H 5.695644476 2.884330853 -2.018023173<br>H 7.131997712 0.849436237 -1.100135124<br>H 5.354345448 -3.151218809 2.581808554<br>H 2.825733871 -3.70674011 3.323890465<br>H 2.016890708 -2.860174529 -0.797288108<br>H 0.304553888 -3.002829242 -2.8018641<br>H -5.247378128 -3.061015139 -2.4121646<br>H -6.883475486 -1.815948896 -0.698832564<br>C -6.841790268 0.259333309 1.336761009<br>C -1.528972031 4.908247298 0.530710681<br>C 3.1530305 4.167422858 -1.497156583

C 6.844779806 -1.44886447 0.740165968
C 0.052948365 -2.005906269 3.013666181
C -2.521493389 -2.881516709 -3.427903741
H -6.859289812 0.45923987 2.41261935
H -7.41801095 1.051250513 0.846734906
H -7.342784259 -0.693413731 1.168523724
H -0.791322705 5.201057125 1.288952651
H -1.209619471 5.332742828 -0.429344583
H -2.477924733 5.380327355 0.782619713
H 3.408280323 4.744016297 -0.597794851
H 4.000875247 4.247240175 -2.17981654
H 2.300348633 4.651759351 -1.983820597
H 6.932824473 -2.529655095 0.594807592
H 7.545548424 -0.954489998 0.068463115
H 7.14979526 -1.230573947 1.769721214
H -0.41489734 -1.015156581 3.091203506
H -0.746770951 -2.750866359 3.09164121
H 0.722175436 -2.108991206 3.892330515
H -1.563766575 -2.803371108 -3.947718302
H -2.779175392 -3.94828342 -3.390308924
H -3.277302999 -2.397435278 -4.077280518

28M$_{1b}$ (1.0 nN at 1-4, C_1)

N 4.0847774293 0.6192458648 -0.4590176777
N 0.6744256479 3.2297315216 -0.3138216056
N -3.4419180186 1.6742504632 0.4954547185
N -3.9741497020 -0.8580710109 -0.2619132612
N -0.9853838927 -2.2322140090 0.1800985260
N 3.2881893216 -1.6070843219 0.6407230227
C 5.6749805603 -1.0197071765 0.3967083052
C 5.3783863126 0.1385217582 -0.2945498258
C 6.2447778987 1.1798049312 -0.8274170457
C 5.4707144261 2.2291525631 -1.2193422529
C 4.0677010955 1.9086019653 -0.9571062502
C 2.9620365198 2.7284925696 -1.0412311401
C 1.6389386633 2.3061148608 -0.6624037211
C 1.0163267572 1.0423011352 -0.5662963239
C -0.3110011584 1.2307091125 -0.1593045563
C -0.5357084698 2.6114987969 0.0183273426
C -1.6295316208 3.3881366791 0.5043780998
C -2.9190881329 2.9432260980 0.7745294249
C -3.9872712536 3.7741564506 1.3273595717
C -5.1168242150 3.0189737417 1.3481079729
C -4.7629295087 1.7142387008 0.8037229595
C -5.7188157355 0.6674973724 0.5432966746
C -5.3056645062 -0.5266029098 -0.0019179604

C -6.0988788620 -1.6141257377 -0.5496760292<br>C -5.2685059111 -2.4782097977 -1.1882741642<br>C -3.8891918674 -2.0213605233 -1.0294264890<br>C -2.7592207877 -2.5926499512 -1.5615947062<br>C -1.3983151875 -2.3348867292 -1.1366939358<br>C -0.2272872862 -2.3819445335 -1.9161063408<br>C 0.8771961899 -2.3086677475 -1.0638884726<br>C 0.4107146812 -2.2378441554 0.2632552328<br>C 1.1343847361 -2.4448442730 1.4788225072<br>C 2.5143642253 -2.3189289410 1.5589758058<br>C 3.4059181967 -2.9430842588 2.5303565821<br>C 4.6829306004 -2.6460127950 2.1490351193<br>C 4.5870308660 -1.7641107624 0.9886796108<br>H 3.3080826091 0.0463940325 -0.1207653433<br>H 0.8421429933 4.2198248713 -0.2647577219<br>H -3.2674852849 -0.1262034288 -0.1449621491<br>H -1.6199349725 -2.2224289414 0.9639992711<br>H 7.3211859274 1.1207734856 -0.8704563766<br>H 5.8244344061 3.1602752337 -1.6320928998<br>H 1.4653019540 0.0950043348 -0.8122204565<br>H -1.0486139003 0.4637430409 0.0072935929<br>H -3.9043421138 4.7935458254 1.6675067844<br>H -6.0940095581 3.3168008281 1.6948297145<br>H -7.1718197202 -1.6895109738 -0.4689210024<br>H -5.5538411081 -3.3884254134 -1.6906829823<br>H -0.2039327009 -2.4628930121 -2.9907576770<br>H 1.9223402314 -2.3349769521 -1.3268784125<br>H 3.1096805837 -3.5743174974 3.3545032342<br>H 5.5978015549 -2.9632875740 2.6265177230<br>C 7.0967936316 -1.4548515885 0.7199624394<br>C 3.1190499511 4.1781761815 -1.4372418990<br>C -1.3037641200 4.8545040797 0.7162221956<br>C -7.1830205406 0.9948426594 0.8141334983<br>C -2.8788839231 -3.6060372158 -2.6791594431<br>C 0.3394832198 -2.9043046302 2.6756935421<br>H 7.2035605825 -2.5311486652 0.5468478257<br>H 7.9096020532 -0.9651650485 0.1542632488<br>H 7.3122625491 -1.2823504881 1.7823633665<br>H 3.0396490936 4.8474762202 -0.5686644556<br>H 4.0884984343 4.3697390026 -1.8965118937<br>H 2.3483561978 4.4721737603 -2.1581852306<br>H -0.4541297043 4.9711756363 1.3984846153<br>H -1.0400257857 5.3283270390 -0.2379195595<br>H -2.1378800185 5.4146102334 1.1261947156<br>H -7.3407248551 1.1457601790 1.8891187054<br>H -7.4468422274 1.9367150040 0.3209282977<br>H -7.9378868421 0.2598665115 0.4890289088

H -2.4729436725 -4.5804124752 -2.3877238186<br>H -3.9122939139 -3.7349286017 -3.0012503043<br>H -2.3126478015 -3.2706796075 -3.5555498797<br>H -0.3730852829 -2.1252784129 2.9768043381<br>H -0.2332546291 -3.8114478139 2.4542450990<br>H 0.9808036603 -3.0942264903 3.5352155017
**28M$_{1b}$** (1.0 nN at 1-5, C_1)<br><br>N 4.124576234 0.692330441 -0.418472506<br>N 0.790251471 3.218684868 -0.711727852<br>N -3.112852902 1.530799779 0.588946111<br>N -3.804796947 -0.982271765 -0.301788824<br>N -1.163558912 -2.233381278 -0.291516143<br>N 3.132901855 -1.490938628 0.630237239<br>C 5.504627865 -0.795458499 0.910661207<br>C 5.355658321 0.289413269 0.069726049<br>C 6.297431434 1.312477839 -0.361460532<br>C 5.606935288 2.281670386 -1.025074077<br>C 4.188554386 1.931223066 -1.024214842<br>C 3.100309028 2.714724854 -1.340859443<br>C 1.762005054 2.293489045 -1.031184794<br>C 1.159877876 1.024353966 -0.878905977<br>C -0.15337533 1.208991681 -0.436382392<br>C -0.389516322 2.593458585 -0.302021146<br>C -1.452720879 3.353178249 0.254776606<br>C -2.646038881 2.839258141 0.746160389<br>C -3.623795133 3.603391741 1.514719489<br>C -4.648055067 2.76019432 1.809479083<br>C -4.326150275 1.479825504 1.192406351<br>C -5.257281766 0.395798447 1.109547859<br>C -5.016193663 -0.709195753 0.325421529<br>C -5.952080522 -1.704959978 -0.166075674<br>C -5.333535868 -2.449090784 -1.122973605<br>C -3.945597885 -2.029301687 -1.219947721<br>C -2.940152029 -2.571737987 -1.985813058<br>C -1.550328133 -2.397374922 -1.612401299<br>C -0.353577809 -2.491401782 -2.352759531<br>C 0.729400233 -2.374008984 -1.467934659<br>C 0.224761271 -2.234792653 -0.158281598<br>C 0.856776379 -2.373154993 1.122080738<br>C 2.211807212 -2.216284803 1.392726342<br>C 2.91366579 -2.790662587 2.539174319<br>C 4.224519071 -2.439323743 2.42844712<br>C 4.337923539 -1.575031718 1.257279215<br>H 3.306892861 0.116314903 -0.203808007<br>H 0.957335782 4.209560883 -0.667565365

H -3.084775274 -0.254920176 -0.296178398
H -1.824674573 -2.157478957 0.467159638
H 7.357861256 1.296923827 -0.164306269
H 6.017942747 3.185736699 -1.445494888
H 1.614238649 0.072785435 -1.100381301
H -0.87484981 0.44277298 -0.209153161
H -3.538560844 4.633746872 1.820761358
H -5.541495722 2.985790602 2.370473349
H -6.976937489 -1.800224675 0.155006801
H -5.762631202 -3.267957837 -1.678122021
H -0.296057212 -2.627999345 -3.421052025
H 1.781675058 -2.421899246 -1.702271414
H 2.484292993 -3.431115352 3.293874687
H 5.028804372 -2.718268235 3.091410517
C 6.832560617 -1.11194751 1.581976606
C 3.289436131 4.130930721 -1.828789106
C -1.199943501 4.844568852 0.340529898
C -6.596464699 0.564004757 1.787218315
C -3.292422641 -3.437098047 -3.182140669
C -0.069646896 -2.786182009 2.246097644
H 7.260017245 -2.041493558 1.188058475
H 7.612740749 -0.332662761 1.498620953
H 6.678603621 -1.264146201 2.655471295
H 3.18399606 4.863975156 -1.015958816
H 4.279901427 4.27630961 -2.261807505
H 2.552713759 4.378368784 -2.599979862
H -0.35748163 5.067870996 1.006303754
H -0.961859344 5.244920889 -0.652435958
H -2.067442261 5.39083329 0.699124218
H -6.465514571 0.821948424 2.843158535
H -7.18815593 1.363041033 1.325976457
H -7.183760936 -0.3541314 1.750489196
H -2.916259966 -4.45899751 -3.061932255
H -4.38199725 -3.488576084 -3.376456043
H -2.829436142 -3.031116424 -4.089864207
H -0.80253987 -1.990717371 2.435912342
H -0.62303147 -3.698383535 1.999270294
H 0.466326893 -2.938821839 3.180113796

28M$_{1b}$ (1.0 nN at 2-5, C_1)

N 4.162755798 0.871014615 -0.430921759
N 0.908606421 3.540402918 -0.286729217
N -3.167415947 1.663488652 0.320647761
N -3.898666373 -1.142853399 -0.39286294
N -1.226588943 -2.382846369 -0.097626232
N 3.071223619 -1.485336689 0.392003686

C 5.502879432 -1.016961268 0.301429174
C 5.379916528 0.228962654 -0.267268593
C 6.390056747 1.232914081 -0.57715543
C 5.762577411 2.417220231 -0.815760978
C 4.321112723 2.219005839 -0.704276735
C 3.298549069 3.139823891 -0.755202738
C 1.927130402 2.692852814 -0.665988653
C 1.313580177 1.42654272 -0.868502652
C -0.047012629 1.536887248 -0.566487234
C -0.314512017 2.864963457 -0.189818859
C -1.48226249 3.542363531 0.257086967
C -2.729924028 2.988890533 0.526369303
C -3.819600614 3.759420817 1.119264576
C -4.871815499 2.91952319 1.267258908
C -4.452598443 1.622274807 0.74561723
C -5.386062946 0.529007221 0.658922957
C -5.126882782 -0.690723632 0.085270133
C -6.078962823 -1.749765649 -0.225726634
C -5.442106497 -2.733034619 -0.907506503
C -4.032820851 -2.393999329 -1.022757481
C -3.036146237 -3.141142342 -1.593201106
C -1.64543835 -2.80188397 -1.345258829
C -0.487417554 -2.905207077 -2.135833686
C 0.618661681 -2.547527002 -1.347815606
C 0.159530769 -2.255563237 -0.047431821
C 0.859993568 -2.252244876 1.198854331
C 2.232444197 -2.113229327 1.320009958
C 3.047828687 -2.718596446 2.36613636
C 4.350331995 -2.521099558 2.014681321
C 4.33789166 -1.69324481 0.814047698
H 3.308878501 0.374479555 -0.191311888
H 1.049448632 4.502705853 -0.032041536
H -3.106279165 -0.509753724 -0.444612974
H -1.852964608 -2.267374274 0.684369295
H 7.454689706 1.059331281 -0.574911592
H 6.234435562 3.357798264 -1.051574087
H 1.78599575 0.529834664 -1.235556404
H -0.791346809 0.75920699 -0.616450056
H -3.794347152 4.795122185 1.414401638
H -5.838694476 3.157720985 1.679606094
H -7.126452606 -1.730890438 0.025284634
H -5.879533682 -3.644735319 -1.280748213
H -0.471704057 -3.202861775 -3.172265877
H 1.660058595 -2.540893696 -1.627514213
H 2.683306911 -3.287549476 3.207811255
H 5.231715096 -2.859801675 2.537887626
C 6.860253273 -1.597478074 0.608678826

C 3.579064026 4.634232467 -0.782113535
C -1.27658725 5.030498161 0.483754928
C -6.780427977 0.804189029 1.185011802
C -3.343488421 -4.361657151 -2.449042682
C 0.034784719 -2.558582691 2.427116101
H 7.639909592 -1.131798157 0.002707337
H 7.13124027 -1.458214403 1.662778054
H 6.87937343 -2.672034895 0.404858533
H 3.146648537 5.116229833 0.106836134
H 4.648278219 4.915458059 -0.770225006
H 3.123266858 5.106643277 -1.660367519
H -0.619821094 5.212270232 1.343778298
H -0.813677131 5.48355198 -0.399842359
H -2.20549271 5.562730151 0.657046516
H -6.739451455 1.147885172 2.223465976
H -7.286444716 1.581418112 0.601863039
H -7.406893715 -0.085822133 1.169809728
H -2.56629904 -5.117744845 -2.307962031
H -4.314938523 -4.85002466 -2.234766244
H -3.351219938 -4.099953293 -3.514882143
H -0.71398275 -1.770004336 2.577726462
H -0.496596936 -3.511849957 2.333228333
H 0.648235649 -2.581422616 3.326005902

28M$_{1b}$ (1.0 nN at 2-6, C_1)

N 3.916829514 0.744256658 -0.43532832
N 0.731596499 3.359900648 -0.694589553
N -3.171867958 1.654874505 0.558118297
N -3.766888608 -0.906193374 -0.279049357
N -1.118374905 -2.402215428 0.039856582
N 2.956930076 -1.529877016 0.888046682
C 5.334492071 -0.860168852 0.755920549
C 5.150731086 0.218587038 -0.080158903
C 6.137002297 1.124201622 -0.6613666
C 5.484495196 2.145806295 -1.276192619
C 4.048269893 1.953264281 -1.120761411
C 3.025527195 2.815416535 -1.439705181
C 1.670388517 2.438233621 -1.103580632
C 1.045995468 1.176040053 -1.015267125
C -0.251608147 1.357389192 -0.520974138
C -0.452959965 2.73109468 -0.295421712
C -1.503009723 3.479844646 0.312120518
C -2.707053523 2.961951419 0.760865298
C -3.725625921 3.717070845 1.487565907
C -4.772419416 2.877871142 1.697100904
C -4.417337817 1.602100829 1.082388788

C -5.346473985 0.519806086 0.929821576
C -5.035639733 -0.60196517 0.197957335
C -5.927513327 -1.595657716 -0.383972868
C -5.218117047 -2.3607851 -1.255184572
C -3.818475327 -1.964916357 -1.194370037
C -2.739995186 -2.520500548 -1.82723709
C -1.397880746 -2.363224975 -1.314283334
C -0.164126812 -2.341495849 -1.97997314
C 0.851933154 -2.348694599 -1.014507481
C 0.252611366 -2.410392235 0.25815152
C 0.868397365 -2.680725026 1.525673926
C 2.199388349 -2.365403676 1.731060632
C 3.078685472 -2.92357784 2.753829077
C 4.337305241 -2.488348525 2.474193216
C 4.231162083 -1.596172681 1.315861057
H 3.065373959 0.303666893 -0.098613999
H 0.916841115 4.344282166 -0.601542145
H -3.038139453 -0.194892998 -0.201770845
H -1.833640751 -2.42267365 0.750312458
H 7.205776433 1.00047998 -0.59033753
H 5.932380305 2.984253296 -1.785960592
H 1.475467909 0.233826533 -1.315535575
H -0.978799975 0.589782157 -0.32025376
H -3.657985232 4.742902461 1.812467487
H -5.699950314 3.100553265 2.201122727
H -6.984019696 -1.669002422 -0.181021501
H -5.593110205 -3.178788189 -1.850128686
H -0.037158735 -2.317902034 -3.050546086
H 1.917414327 -2.343773576 -1.171913654
H 2.786102315 -3.603899862 3.53932059
H 5.245596552 -2.723769428 3.007880088
C 6.723634755 -1.265708235 1.187982852
C 3.250927764 4.231170824 -1.954576194
C -1.219831171 4.958414089 0.477799571
C -6.743189493 0.711509678 1.470948911
C -2.902338384 -3.399306194 -3.040635731
C 0.051101861 -3.370939825 2.608319651
H 7.494223908 -0.761484315 0.603871351
H 6.900688849 -1.025865068 2.243491226
H 6.871756714 -2.343771118 1.06898005
H 3.16843264 4.957426464 -1.132093477
H 4.219187173 4.442271513 -2.441619464
H 2.47490641 4.482226513 -2.685884782
H -0.35296073 5.122653241 1.128977042
H -1.002977501 5.414782874 -0.495797868
H -2.061992131 5.498465501 0.900052363
H -6.715250762 0.951437462 2.539277918

H -7.267248634 1.530895224 0.965821006
H -7.344259386 -0.191845327 1.360766454
H -2.568465556 -4.424361548 -2.846986375
H -3.938509687 -3.42205532 -3.382700841
H -2.296318647 -3.015425202 -3.869716525
H -0.903562345 -2.84828629 2.744651007
H -0.195252065 -4.43738314 2.424547506
H 0.570550139 -3.316103246 3.565742411

28M1b (1.0 nN at 3-5, C_1)

N 3.967497826 0.662467792 -0.54748036
N 0.772663092 3.38741653 -0.325979277
N -3.149637683 1.727966402 0.572542323
N -3.73209371 -0.997843824 -0.470335867
N -1.095512569 -2.328734911 -0.051810421
N 3.054958255 -1.504812695 0.675005275
C 5.442829866 -0.970659859 0.483689159
C 5.225689967 0.128721641 -0.318177625
C 6.159457847 1.077752974 -0.911564697
C 5.452632353 2.13770913 -1.391157913
C 4.033221959 1.917426224 -1.124680508
C 2.978590208 2.794660305 -1.23364779
C 1.664538816 2.434090136 -0.776800946
C 1.013758829 1.188295308 -0.640631404
C -0.255793231 1.414864321 -0.103411834
C -0.414842888 2.798857704 0.110178115
C -1.466876197 3.55428177 0.709310657
C -2.718137577 3.011922453 0.962720609
C -3.820106689 3.706800757 1.61210495
C -4.885217634 2.862660692 1.599759267
C -4.446023473 1.635697848 0.929739784
C -5.344693426 0.552616362 0.623330415
C -5.008047802 -0.591083904 -0.067367154
C -5.916393574 -1.598502899 -0.600335461
C -5.210483724 -2.496323073 -1.333305822
C -3.798263464 -2.166452076 -1.260109607
C -2.750645331 -2.870823285 -1.807155696
C -1.396041681 -2.642160463 -1.363657264
C -0.167409398 -2.769716868 -2.034909457
C 0.861527959 -2.523426226 -1.117790825
C 0.281026848 -2.274743626 0.143360137
C 0.874875302 -2.291029951 1.44284736
C 2.233340014 -2.10960669 1.62574594
C 3.061109423 -2.603271129 2.71956687
C 4.358490938 -2.354688294 2.374495233
C 4.325280615 -1.622421606 1.111126156

H 3.153866159 0.165803868 -0.188817732
H 0.974427874 4.372406197 -0.287803353
H -2.954088034 -0.348018301 -0.413098986
H -1.795757926 -2.220488763 0.666602657
H 7.231572154 0.960868045 -0.931304232
H 5.861832636 3.01791996 -1.860949106
H 1.397496769 0.22665526 -0.943896551
H -1.003478624 0.680803772 0.1368747
H -3.787654741 4.7041248 2.022578142
H -5.872211747 3.055340295 1.989377687
H -6.983546386 -1.605428295 -0.452385038
H -5.602468454 -3.359750939 -1.847374127
H -0.05234106 -3.005693662 -3.080554193
H 1.924824349 -2.547438475 -1.289756266
H 2.713009633 -3.128642635 3.596076412
H 5.246257678 -2.608891439 2.933869769
C 6.835287189 -1.415756071 0.848373759
C 3.193971443 4.217227013 -1.688469559
C -1.127414576 5.010908284 1.017212374
C -6.786053507 0.758947125 1.042764243
C -2.977760453 -3.911890013 -2.894398888
C -0.022647906 -2.616446413 2.609321143
H 7.589139659 -0.927723103 0.22805469
H 7.068102579 -1.183650097 1.894894948
H 6.948707529 -2.497067975 0.721165853
H 3.198950319 4.919633772 -0.842619117
H 4.147919208 4.334966919 -2.203562639
H 2.403741798 4.531201377 -2.378736777
H -0.156171421 5.057388891 1.523150726
H -1.033177092 5.569054774 0.075697713
H -1.826161919 5.582648447 1.64592017
H -6.845707503 0.972034884 2.115064403
H -7.241046674 1.604983056 0.515807669
H -7.400725905 -0.120499993 0.859143058
H -2.882693934 -4.975572682 -2.591185936
H -3.965328861 -3.786834592 -3.343582876
H -2.246540095 -3.758427908 -3.696097936
H -0.769567691 -1.823024711 2.743564551
H -0.560063435 -3.558313584 2.455153784
H 0.54000587 -2.680105783 3.540017369

28M$_{1b}$ (1.0 nN at 5-6, C_1)

N 3.91442575 0.522318435 -0.592419833
N 0.66587625 3.159753214 -0.518444619
N -3.215492319 1.514690248 0.697481212
N -3.751847311 -0.889071403 -0.394192263

N	-1.106001979	-2.100821756	0.048488587
N	3.031044766	-1.499911525	0.819650677
C	5.417106753	-1.011958076	0.542380788
C	5.182948386	0.024318564	-0.34334355
C	6.09293655	0.935156969	-1.015523839
C	5.361697887	1.948851375	-1.564785287
C	3.953708135	1.724748989	-1.265599991
C	2.874587908	2.576527597	-1.421998079
C	1.579397339	2.21414039	-0.941381618
C	0.961773749	0.954004087	-0.743144635
C	-0.29705984	1.165189718	-0.188011822
C	-0.492392346	2.555758409	-0.022257168
C	-1.514740681	3.305915244	0.601746933
C	-2.739735038	2.78111082	1.015917519
C	-3.764939681	3.499195978	1.76232015
C	-4.841162997	2.669499487	1.864277066
C	-4.491776241	1.444117437	1.162875477
C	-5.417387361	0.405687747	0.840633857
C	-5.060005427	-0.64087823	0.012853651
C	-5.892309132	-1.555157069	-0.740164283
C	-5.10302719	-2.22937519	-1.628778399
C	-3.72514513	-1.848656731	-1.40398883
C	-2.581861954	-2.36777474	-1.980898685
C	-1.311762591	-2.320028523	-1.314918461
C	-0.037483584	-2.615012675	-1.85528125
C	0.90432095	-2.550861239	-0.838386961
C	0.24883792	-2.228312988	0.37985874
C	0.841812791	-2.192631273	1.679442713
C	2.227857488	-2.060414591	1.800281536
C	3.074289368	-2.543028409	2.882629375
C	4.369826354	-2.329141495	2.498454633
C	4.318467189	-1.629794368	1.222222447
H	3.116731389	0.040731614	-0.170355678
H	0.849570252	4.148791045	-0.499760774
H	-3.062841018	-0.14623483	-0.220110558
H	-1.855318112	-1.900541295	0.695834688
H	7.166465784	0.833048591	-1.04256658
H	5.750676838	2.796050052	-2.106716774
H	1.372128099	-0.004474861	-1.016704316
H	-1.009642862	0.414441865	0.099242967
H	-3.686378633	4.494964751	2.169013435
H	-5.784435112	2.876533247	2.345929666
H	-6.9614655	-1.648772645	-0.633632195
H	-5.417136999	-2.980681687	-2.336450269
H	0.155498383	-2.8518864	-2.888701539
H	1.964906304	-2.718617827	-0.914945818
H	2.739024509	-3.043473318	3.778525139

H 5.265509054 -2.588693019 3.042855618
C 6.819940736 -1.41008919 0.921494269
C 3.068387492 3.981853214 -1.93622817
C -1.22437309 4.777484274 0.796433433
C -6.847667586 0.557218062 1.297156841
C -2.661845142 -3.076491593 -3.326603056
C 0.024374287 -2.337568874 2.946686355
H 7.559131818 -0.964495371 0.253650117
H 7.061510025 -1.092827226 1.943108312
H 6.943777457 -2.49674061 0.879385429
H 3.104772366 4.715365438 -1.117931313
H 4.002063165 4.080355514 -2.491456562
H 2.25186553 4.269314132 -2.60691444
H -0.302793176 4.92462805 1.371340151
H -1.097907855 5.273337064 -0.174728127
H -2.029643012 5.29325646 1.312906448
H -6.892452159 0.713595149 2.379944539
H -7.335037678 1.41609934 0.822301656
H -7.436612323 -0.333358003 1.074038509
H -2.582416846 -4.164321646 -3.214043429
H -3.577506445 -2.857364384 -3.908201798
H -1.823292877 -2.761375243 -3.956279815
H -0.700614682 -1.516438928 3.020742519
H -0.541865422 -3.275480266 2.963353222
H 0.659903281 -2.284136792 3.851696941

28M₂ (1.0 nN at 1-4, C_1)

N 4.0523700883 0.5550538933 -0.5120992231
N 0.6723004342 3.2048202234 -0.4842732754
N -3.4149293167 1.6812986254 0.5368726255
N -3.8136567620 -0.8845442306 -0.2123493110
N -1.0178974604 -2.0732222168 0.1033824793
N 3.2032321236 -1.5626248381 0.7142640434
C 5.6003514562 -1.0400920985 0.4927532702
C 5.3334779054 0.0754242163 -0.2906830423
C 6.2222573443 1.0755972332 -0.8552245256
C 5.4661434245 2.1137435189 -1.3180400344
C 4.0593910412 1.8199289088 -1.0711423145
C 2.9595074669 2.6491776288 -1.1940296826
C 1.6326675624 2.2577638888 -0.8124234041
C 0.9961206590 1.0073046081 -0.6784182640
C -0.3259013478 1.2204726467 -0.2621535911
C -0.5340660091 2.6099277986 -0.1186664793
C -1.6161834800 3.4097497264 0.3680935767
C -2.8806077651 2.9559085989 0.7102968981
C -3.9490788870 3.7279656484 1.3233857519

C -5.0436273781 2.9318608894 1.4840994817
C -4.7370441342 1.6194613180 0.9597417039
C -5.5858705197 0.5339612947 0.7755268054
C -5.1175144372 -0.6171013372 0.0771456855
C -5.9906516769 -1.5744610087 -0.5991472568
C -5.1949535799 -2.3535381889 -1.3786681122
C -3.8153547925 -1.9400150757 -1.1293025690
C -2.6833898240 -2.4728629971 -1.7199074763
C -1.3547385611 -2.3165317311 -1.2183353279
C -0.1412240140 -2.5629049239 -1.8996643766
C 0.9043775622 -2.4554967421 -0.9897450505
C 0.3564336161 -2.1726487931 0.2845564627
C 1.0077594441 -2.2572658139 1.5549695886
C 2.3880234153 -2.1598588651 1.6718011808
C 3.2375783087 -2.7046239996 2.7294249007
C 4.5310262101 -2.4830463995 2.3559314794
C 4.4921647708 -1.7140530452 1.1108610330
H 3.2640201277 0.0048541871 -0.1565901926
H 0.8511574021 4.1937185846 -0.4495426131
H -3.0088676640 0.8621685208 0.0867885468
H -1.7125115259 -1.8301992098 0.7973445340
H 7.2987940956 1.0055698424 -0.8667782827
H 5.8397652789 3.0200804845 -1.7670982043
H 1.4299578354 0.0465839832 -0.9035977687
H -1.0491329510 0.4417828513 -0.0794237046
H -3.8793531582 4.7629551239 1.6142225190
H -5.9939819099 3.2202893496 1.9037142051
H -7.0671602515 -1.6037020813 -0.5202105361
H -5.5078078149 -3.1603958075 -2.0230575945
H -0.0566928305 -2.7988333991 -2.9480626803
H 1.9584830943 -2.5915362237 -1.1659315406
H 2.8995850991 -3.2391315138 3.6044217267
H 5.4240397608 -2.7740416107 2.8887582775
C 7.0125100318 -1.4415513050 0.8966192416
C 3.1425704140 4.0844646085 -1.6295248670
C -1.3042647250 4.8841627930 0.5195298615
C -7.0283626042 0.5859316938 1.2683675743
C -2.8017870613 -3.3034664455 -2.9767801799
C 0.1439530632 -2.5445025714 2.7559321271
H 7.0845112597 -2.5326239267 0.9479742585
H 7.8322838278 -1.1092615026 0.2340089386
H 7.2484480675 -1.0590206126 1.8983820178
H 3.0757218873 4.7766267836 -0.7781759978
H 4.1152703017 4.2457920757 -2.0935262542
H 2.3758975496 4.3723480780 -2.3567300915
H -0.4629773953 5.0428266668 1.2050092824
H -1.0368503322 5.3237582857 -0.4501548069

H -2.1493956121 5.4532506557 0.8978614038
H -7.3301904511 -0.4078212790 1.6128194285
H -7.1027341986 1.2507464863 2.1332031446
H -7.8072290880 0.9178074554 0.5501366079
H -3.8352105151 -3.3807909587 -3.3109895914
H -2.2354800401 -2.836048912 -3.7904215784
H -2.3964592196 -4.3099220794 -2.8340879247
H -0.5306662317 -1.6996765812 2.9469313954
H -0.4770023575 -3.4334904242 2.6025353370
H 0.7422418538 -2.6817250664 3.6564126602

28M₂ (1.0 nN at 1-5, C_1)

N 4.08923071 0.665133204 -0.495465408
N 0.780247207 3.182648486 -0.74940499
N -3.105802012 1.500093994 0.644952537
N -3.708902897 -1.055805975 -0.269365519
N -1.172524705 -2.130671639 -0.285976782
N 3.085709544 -1.438706338 0.675090369
C 5.460714612 -0.777777077 0.900585144
C 5.314736833 0.260530287 -0.007478178
C 6.270066982 1.246500447 -0.485341889
C 5.587115457 2.21182695 -1.167475149
C 4.165993814 1.886418705 -1.138743609
C 3.078066342 2.674929306 -1.448927663
C 1.743027826 2.258191013 -1.123489957
C 1.138148829 0.994437133 -0.99229973
C -0.165866846 1.173948797 -0.509197885
C -0.386582611 2.554977121 -0.323814604
C -1.425965522 3.313487079 0.29708784
C -2.598334816 2.789618492 0.806669423
C -3.557398734 3.471297839 1.656680472
C -4.567295869 2.613013204 1.967977556
C -4.325794499 1.355609874 1.293037644
C -5.196620186 0.288287386 1.156235669
C -4.926100373 -0.776004716 0.252751725
C -5.951337481 -1.603030721 -0.381174821
C -5.330465331 -2.326544635 -1.352055743
C -3.908503496 -2.013280229 -1.273448156
C -2.883986013 -2.575503423 -2.014068788
C -1.515478403 -2.455369266 -1.587964099
C -0.295355354 -2.720359924 -2.254618002
C 0.753177628 -2.533949229 -1.34749558
C 0.196958765 -2.185713316 -0.09199515
C 0.7762893 -2.166436767 1.223147828
C 2.126733245 -2.018179526 1.508901684
C 2.789643577 -2.451821847 2.741252195

C 4.114957413 -2.176896458 2.605649044
C 4.280950004 -1.483536111 1.326395882
H 3.264406629 0.108897951 -0.254644202
H 0.95410098 4.170639162 -0.67520566
H -2.783003703 0.763073107 0.02548688
H -1.883083266 -1.810392922 0.364944554
H 7.331436763 1.216644088 -0.293085823
H 6.008858176 3.097117719 -1.616674851
H 1.579639958 0.044995075 -1.250948738
H -0.859470007 0.375914661 -0.301685223
H -3.461121524 4.486005738 2.006038866
H -5.425086147 2.824369274 2.585385318
H -7.004811439 -1.589779104 -0.146058932
H -5.789231351 -3.034601743 -2.024515176
H -0.203648138 -3.016572899 -3.287954978
H 1.809844125 -2.669141628 -1.517596344
H 2.320751766 -2.954409785 3.573057999
H 4.893620319 -2.38284406 3.323767797
C 6.829106599 -1.102498219 1.483566566
C 3.269588343 4.086744208 -1.947218643
C -1.154790059 4.794724538 0.448974204
C -6.548989353 0.339811595 1.829532479
C -3.199191747 -3.37287864 -3.265272884
C -0.204664172 -2.387194725 2.353267534
H 7.442181054 -1.628431434 0.741164154
H 7.430908325 -0.23874534 1.833374179
H 6.727345018 -1.778160624 2.335048152
H 3.173077205 4.822956365 -1.136322499
H 4.258546777 4.225685697 -2.385926857
H 2.52747527 4.333557249 -2.712927054
H -0.285726749 4.981082186 1.092136939
H -0.954019394 5.248893589 -0.529522326
H -2.003296485 5.328850544 0.870195467
H -6.500776563 0.880188507 2.777402794
H -7.304342343 0.831627247 1.203854375
H -6.906852693 -0.668225123 2.052082173
H -2.717960018 -2.909179846 -4.13477109
H -2.806790994 -4.392332667 -3.188430544
H -4.281069287 -3.434799516 -3.48776479
H -0.907173291 -1.544676319 2.405634895
H -0.794314878 -3.297509913 2.20417244
H 0.292659255 -2.438260756 3.319947868

28M₂ (1.0 nN at 2-5, C_1)

N 4.169034293 0.871553987 -0.581680462
N 0.93324203 3.499386014 -0.390412019

N -3.105889388 1.555043211 0.457762744
N -3.803320726 -1.254955149 -0.304743715
N -1.271826378 -2.20077699 -0.27555437
N 3.010686738 -1.401722876 0.319818707
C 5.449113876 -0.979387508 0.321020928
C 5.370176889 0.231502269 -0.338374053
C 6.398811879 1.215277382 -0.641768318
C 5.788272291 2.397394282 -0.945290821
C 4.345542586 2.21197606 -0.879021908
C 3.321558127 3.135351694 -0.945177032
C 1.955556648 2.682215958 -0.844118954
C 1.353837742 1.419319158 -1.068339617
C 0.004270889 1.492498744 -0.703787961
C -0.269426928 2.800514075 -0.260143589
C -1.415527989 3.444340244 0.296749772
C -2.619865254 2.859441783 0.65742032
C -3.661723491 3.532195137 1.40813659
C -4.688095284 2.671418991 1.632012956
C -4.380076725 1.405619237 1.006481561
C -5.261006529 0.341361317 0.889996035
C -5.01163454 -0.834258597 0.130450402
C -6.067879836 -1.723740099 -0.362921412
C -5.46452477 -2.667577339 -1.130960161
C -4.033236424 -2.401856159 -1.08632347
C -3.041381016 -3.160606689 -1.682437494
C -1.660685611 -2.897556632 -1.400705225
C -0.466438582 -3.304159924 -2.047822804
C 0.614708051 -2.839625924 -1.298329732
C 0.103443367 -2.182466131 -0.147975559
C 0.734471525 -1.950414639 1.112534117
C 2.108224912 -1.858619235 1.281619348
C 2.848035216 -2.33442353 2.445606617
C 4.172678585 -2.243604941 2.133649467
C 4.251244572 -1.585720388 0.832507459
H 3.3029126 0.387469157 -0.357231543
H 1.070732772 4.44873048 -0.089416519
H -2.695759095 0.811350049 -0.091122869
H -1.972014098 -1.727979145 0.293322059
H 7.461272562 1.03755483 -0.584978224
H 6.276892639 3.327757012 -1.188063598
H 1.823774587 0.544326267 -1.487859393
H -0.698059763 0.67726674 -0.782574603
H -3.610732413 4.548107376 1.760326178
H -5.599210661 2.886383595 2.16459042
H -7.125320041 -1.609557173 -0.18199767
H -5.939810555 -3.474128071 -1.667627567
H -0.416622254 -3.87442731 -2.961934077

H 1.666178406 -2.993594149 -1.480762904
H 2.417704469 -2.760122398 3.339514341
H 5.0137664 -2.538485781 2.743055285
C 6.784040553 -1.538957185 0.744911539
C 3.603787569 4.62904954 -0.972910531
C -1.229066925 4.927188544 0.566395319
C -6.632390958 0.490954105 1.52166644
C -3.39517176 -4.299597972 -2.626608152
C -0.171587722 -1.953914784 2.320591106
H 7.592492556 -1.153675821 0.119986864
H 7.019792606 -1.284520216 1.78596319
H 6.79326926 -2.629440501 0.662947441
H 3.145050736 5.100880425 -1.849533599
H 3.174552668 5.112772072 -0.083389041
H 4.673790164 4.908018453 -0.965320837
H -0.542389513 5.104447647 1.404377215
H -0.816276733 5.419813638 -0.321197802
H -2.163300684 5.433988201 0.790307883
H -6.570558652 0.992027005 2.490197458
H -7.321148212 1.066975534 0.891384655
H -7.081217044 -0.48633809 1.703628413
H -3.588322173 -3.919647851 -3.636860512
H -2.563910158 -5.005006206 -2.692393345
H -4.289547748 -4.880295384 -2.32483421
H -0.842150406 -1.085188662 2.280747773
H -0.799202656 -2.850139222 2.355741044
H 0.396974834 -1.872476182 3.246206359

28M$_2$ (1.0 nN at 2-6, C_1)

N 3.867417342 0.703187874 -0.527521103
N 0.698940949 3.272382902 -0.825510702
N -3.1401906 1.616753364 0.66069153
N -3.641486332 -0.953514266 -0.201931224
N -1.128931989 -2.236206859 0.038850821
N 2.888405041 -1.446124071 0.938858833
C 5.26797038 -0.799647033 0.801018526
C 5.095598092 0.211837513 -0.123169811
C 6.085782379 1.077885123 -0.755550813
C 5.435382465 2.053065361 -1.446386524
C 3.999709012 1.867791299 -1.288905829
C 2.971307252 2.70746631 -1.646247545
C 1.622606123 2.341110105 -1.268230795
C 1.005425934 1.087145437 -1.129441211
C -0.270194937 1.275791427 -0.57042783
C -0.457890095 2.652980555 -0.353291667
C -1.467471642 3.423564482 0.31422646

C -2.639940591 2.908807076 0.821487359
C -3.63280208 3.597778001 1.632275844
C -4.655685454 2.744658874 1.90725712
C -4.385071016 1.475841371 1.256212425
C -5.239298792 0.40344491 1.083130914
C -4.897944134 -0.661054269 0.196247233
C -5.869448078 -1.448952399 -0.564789773
C -5.166417816 -2.151029913 -1.495322198
C -3.755718464 -1.872850158 -1.257064824
C -2.664297991 -2.415989845 -1.896578611
C -1.35529666 -2.370437399 -1.320045939
C -0.102251623 -2.592521575 -1.920867392
C 0.868549581 -2.560143176 -0.916963657
C 0.214878721 -2.362098505 0.319173207
C 0.778076129 -2.508665939 1.637747206
C 2.103912856 -2.188595523 1.843541791
C 2.956570775 -2.644986831 2.941507232
C 4.223619625 -2.252083078 2.646905405
C 4.152388746 -1.476737105 1.399516742
H 3.012953062 0.278715369 -0.176153336
H 0.886555324 4.258039445 -0.752019604
H -2.776346443 0.858822777 0.092993789
H -1.874266499 -1.952173876 0.664074197
H 7.154094464 0.965404687 -0.661828115
H 5.886449918 2.857439893 -2.00613658
H 1.420299661 0.136245809 -1.426364917
H -0.950351296 0.474461323 -0.328468627
H -3.55573499 4.61894286 1.967943853
H -5.54094874 2.963858128 2.482001357
H -6.940845007 -1.42059099 -0.4338972
H -5.562227562 -2.82662705 -2.238323081
H 0.064146224 -2.758421753 -2.973379093
H 1.932157669 -2.696226462 -1.015376562
H 2.636159705 -3.236573483 3.785911813
H 5.118527874 -2.438262116 3.221464603
C 6.650355702 -1.162912772 1.289176383
C 3.187439081 4.092601719 -2.240905718
C -1.163615491 4.894939594 0.487528621
C -6.634482995 0.445779299 1.661215278
C -2.797147255 -3.130893817 -3.215173352
C -0.084308613 -3.091060575 2.747076897
H 7.427936325 -0.732340782 0.656750676
H 6.823842348 -0.807142501 2.31224858
H 6.793616316 -2.248068878 1.291667706
H 2.401595731 4.302144754 -2.974334407
H 3.112876539 4.862381261 -1.458349987
H 4.150434735 4.276757448 -2.749560265

H -0.25244684 5.046948605 1.079159095<br>H -1.016473583 5.376650461 -0.487665077<br>H -1.971926711 5.42890775 0.982035643<br>H -6.662343944 1.016379906 2.592192102<br>H -7.354934729 0.902132569 0.970443909<br>H -6.988309715 -0.562876584 1.889772689<br>H -3.819540949 -3.091876603 -3.590614904<br>H -2.153617294 -2.657480291 -3.965392198<br>H -2.486134732 -4.17729409 -3.134699564<br>H 0.37246504 -2.904165236 3.720340728<br>H -1.0635121 -2.59906062 2.756062815<br>H -0.285007438 -4.180853856 2.677302979
**28M₂** (1.0 nN at 3-5, C_1)<br><br>N 3.932559829 0.607893311 -0.653232378<br>N 0.763147647 3.312987728 -0.449330821<br>N -3.115863768 1.704266387 0.636858538<br>N -3.618904417 -1.042959459 -0.432257519<br>N -1.120717928 -2.171359346 -0.136565397<br>N 2.980968868 -1.43950463 0.696190816<br>C 5.37394145 -0.929884881 0.547725041<br>C 5.182746778 0.103712114 -0.350652653<br>C 6.128006633 1.021583291 -0.974546552<br>C 5.427144461 2.046942225 -1.536452395<br>C 4.004060531 1.834031447 -1.291111754<br>C 2.944688656 2.701703603 -1.43276807<br>C 1.640879021 2.352082587 -0.935600451<br>C 1.009128498 1.113488635 -0.738595591<br>C -0.226347629 1.341787092 -0.116540067<br>C -0.381305917 2.728669367 0.080392893<br>C -1.392789324 3.498194291 0.752961029<br>C -2.625593302 2.955641515 1.044320801<br>C -3.698270682 3.543973989 1.820517391<br>C -4.750178631 2.681499533 1.855891737<br>C -4.414600153 1.495350983 1.082674201<br>C -5.241296372 0.437268151 0.752110752<br>C -4.870501326 -0.646782667 -0.109908786<br>C -5.859880506 -1.45259839 -0.832342221<br>C -5.171919502 -2.307627 -1.633449957<br>C -3.75519749 -2.088772773 -1.37559555<br>C -2.703227095 -2.811366725 -1.914788123<br>C -1.377174826 -2.680543767 -1.394083711<br>C -0.131152526 -3.064810683 -1.936753378<br>C 0.858970758 -2.760013461 -1.002790019<br>C 0.227838904 -2.223727594 0.146720239<br>C 0.758446228 -2.070859498 1.470040314

C 2.112808032 -1.909711863 1.681610351<br>C 2.893344503 -2.277385286 2.86057022<br>C 4.205474229 -2.096814992 2.535558172<br>C 4.233169004 -1.515453288 1.191207356<br>H 3.113242539 0.124853075 -0.286652526<br>H 0.95736044 4.300388116 -0.437617133<br>H -2.689814117 1.067803794 -0.023243805<br>H -1.866677757 -1.727965724 0.394962029<br>H 7.201179213 0.91464901 -0.953495924<br>H 5.844647297 2.902841431 -2.042943667<br>H 1.375719324 0.144698857 -1.041943168<br>H -0.896871053 0.563097842 0.205803431<br>H -3.644490247 4.509767187 2.297121935<br>H -5.690629857 2.843782405 2.355825276<br>H -6.931111386 -1.343571275 -0.760403758<br>H -5.58470284 -3.041828556 -2.308214223<br>H 0.017913445 -3.513478816 -2.905697106<br>H 1.920045425 -2.928983318 -1.078784178<br>H 2.501681891 -2.684570589 3.780584223<br>H 5.067758065 -2.29612565 3.154196969<br>C 6.754051162 -1.328797151 1.002656318<br>C 3.150541032 4.104769777 -1.946382839<br>C -1.019664372 4.935416082 1.10044256<br>C -6.68036304 0.463390643 1.227668262<br>C -2.909391166 -3.767259755 -3.0796657<br>C -0.206412717 -2.198081022 2.61953973<br>H 7.525282814 -0.914987312 0.350121083<br>H 6.960002688 -0.976871793 2.02123085<br>H 6.870098007 -2.417129424 1.003398965<br>H 3.146062163 4.841384495 -1.13020798<br>H 4.10666284 4.208006246 -2.460916718<br>H 2.360419287 4.383684966 -2.651312313<br>H -0.022491821 4.95198929 1.55608608<br>H -0.969407225 5.541067692 0.184606776<br>H -1.678459977 5.485583862 1.791604981<br>H -6.791964402 1.055191937 2.136988608<br>H -7.355780916 0.884776889 0.472601201<br>H -7.029358193 -0.546198156 1.457844867<br>H -3.867757461 -3.578941952 -3.564906325<br>H -2.131284701 -3.593699824 -3.830439483<br>H -2.868732394 -4.848106603 -2.832006983<br>H -0.90920526 -1.354580947 2.615760982<br>H -0.797834811 -3.116406242 2.548089136<br>H 0.310921347 -2.177954074 3.578837434
28M2 (1.0 nN at 3-6, C_1)

**28M₂**

$28M_2$ (1.0 nN at 5-6, C_1)

N 3.942146139 0.605966091 -0.411540359
N 0.596164623 3.271432998 -0.637061958
N -3.334463044 1.604603218 0.561348709
N -3.676427331 -0.925323297 -0.343252391
N -1.084684284 -2.01370854 0.075517963
N 3.039804907 -1.428802223 0.92691624
C 5.426437034 -1.065852406 0.584046765
C 5.195601044 0.038539872 -0.282532068
C 6.083460433 0.873148788 -1.001085042
C 5.342544619 1.932350849 -1.52770944
C 3.986384466 1.759957057 -1.136813125
C 2.86951312 2.652703859 -1.273091479
C 1.596043599 2.320601147 -0.869715271
C 0.968448293 1.041586961 -0.574266103
C -0.311015751 1.255557145 -0.16086429
C -0.57728747 2.685642484 -0.150556495
C -1.639788277 3.425119618 0.309790755
C -2.859707083 2.863828386 0.821000361
C -3.821564862 3.493091448 1.650822536
C -4.866709882 2.595882315 1.864322268
C -4.571183492 1.40804655 1.162161179
C -5.407887658 0.293665138 0.886108489
C -5.002777318 -0.751121352 0.068834168
C -5.84893715 -1.67800525 -0.662963473
C -5.041502626 -2.33324898 -1.551045596
C -3.685653979 -1.868090014 -1.307977647
C -2.492816012 -2.341921939 -1.953805357
C -1.286164605 -2.323866406 -1.283603997
C 0.03084069 -2.696775511 -1.778533396
C 0.935533642 -2.587783902 -0.774594885
C 0.254210559 -2.173160584 0.454922919
C 0.826909241 -2.098175593 1.710374385
C 2.273709301 -2.101554121 1.794826408
C 3.110246287 -2.830999522 2.738985149
C 4.410130452 -2.619751144 2.361860183
C 4.377862257 -1.706466609 1.233033669
H 3.158427527 0.13704794 0.0576191
H 0.765880387 4.26213721 -0.636345625
H -2.958805348 0.876319045 -0.044712909
H -1.847028285 -1.669465406 0.643898547
H 7.146391343 0.720616658 -1.106158538
H 5.728883872 2.747639392 -2.117095631
H 1.420933356 0.070093967 -0.705210118

H -1.008614145 0.481484787 0.115956376
H -3.738305032 4.487283004 2.058218625
H -5.75577623 2.77174056 2.449055802
H -6.918988526 -1.779546398 -0.56192912
H -5.328364732 -3.080524302 -2.275403369
H 0.234674444 -3.011456049 -2.789339983
H 1.992028465 -2.79158279 -0.818794386
H 2.754229514 -3.458504621 3.542261568
H 5.295060028 -3.027370133 2.827375721
C 6.856501602 -1.450372563 0.857348924
C 3.150380762 4.0689385 -1.71341404
C -1.506610866 4.93146366 0.304159077
C -6.81988494 0.35478475 1.411299405
C -2.620049879 -2.949324498 -3.345338013
C 0.05978748 -1.988586158 3.013827128
H 7.424589866 -1.498765356 -0.076237244
H 7.344719028 -0.710585482 1.500975445
H 6.920623019 -2.425425217 1.34057347
H 3.36394322 4.728127875 -0.861304099
H 4.016297237 4.10960282 -2.376277396
H 2.300401558 4.484289905 -2.264122733
H -0.746864303 5.277971944 1.016682569
H -1.21945005 5.290375392 -0.692497168
H -2.450023055 5.416260722 0.552681845
H -6.815478819 0.565848283 2.485394157
H -7.390544588 1.151687948 0.922968126
H -7.340777161 -0.590346394 1.262073975
H -1.668267554 -2.902464179 -3.879519069
H -2.896090091 -4.010079534 -3.27838571
H -3.376591888 -2.467453223 -3.995608745
H -0.457384442 -1.021971341 3.078826144
H -0.700847639 -2.771648871 3.110770085
H 0.73716088 -2.042853021 3.890990361

28H (0 nN, C_1) H-substituent on the 6-position

N 3.974231918 -1.464493316 0.082274147
N 0.098668866 -3.507494644 -0.328923719
N -3.797169734 -1.38935202 0.088583025
N -3.945305287 1.467219769 0.043090561
N -0.099631475 3.542351063 -0.203553135
N 3.833610334 1.326801497 -0.02024134
C 5.932406266 0.026998121 0.167570914
C 5.3423018 -1.207538444 0.140270284
C 5.99034458 -2.515468674 0.143892413
C 5.039423027 -3.471641151 0.063812147
C 3.720007601 -2.837022179 0.026754631

C 2.515249324 -3.477415182 -0.051096987
C 1.228443678 -2.824579343 0.056808997
C 0.784892055 -1.601628752 0.589220204
C -0.623257874 -1.576409376 0.514047043
C -1.055488828 -2.774982985 -0.068888253
C -2.336871228 -3.373127652 -0.306705547
C -3.560732581 -2.762936504 -0.154704728
C -4.840826522 -3.468474512 -0.164951892
C -5.802581528 -2.551041278 0.096045267
C -5.127642097 -1.259873501 0.226748637
C -5.861803251 -0.032826033 0.412674201
C -5.290193515 1.202147562 0.290085664
C -5.949697655 2.504329546 0.325893029
C -5.028450871 3.464068076 0.09199621
C -3.716520204 2.839565689 -0.087074954
C -2.532741753 3.491495436 -0.285933618
C -1.256569498 2.826069319 -0.406706571
C -0.859235313 1.509801816 -0.705109394
C 0.546927397 1.455178756 -0.652692966
C 1.021683718 2.729625294 -0.326055888
C 2.308262725 3.294277763 -0.136177668
C 3.532476757 2.70177707 -0.024585555
C 4.764276879 3.4709387 0.118648872
C 5.780060679 2.577166787 0.194794587
C 5.168888536 1.245010038 0.111428456
H 3.321176955 -0.690858928 -0.002852913
H 0.114076255 -4.41322989 -0.763349723
H -3.27698751 0.705909582 0.015941332
H -0.06861978 4.508852419 0.076562292
H 7.054478692 -2.676163044 0.197725383
H 5.211569514 -4.534968984 0.05299093
H 1.408564304 -0.838879909 1.029326014
H -1.28037564 -0.80020969 0.868506541
H -4.993489265 -4.525905622 -0.307331626
H -6.864393719 -2.720836886 0.17796341
H -7.003024017 2.657091807 0.494011879
H -5.214556218 4.524237655 0.046765792
H -1.507758842 0.692588835 -0.980254604
H 1.172022432 0.601520999 -0.851515709
H 4.817507266 4.549818818 0.146822917
H 6.833606516 2.780982664 0.303131512
C 7.433737287 0.165897253 0.251134718
C 2.461525053 -4.974896347 -0.263228349
C -2.282511318 -4.827215062 -0.73155085
C -7.343411734 -0.165182181 0.680003402
C -2.487185617 5.003540856 -0.318759887
H 7.827337633 0.724160578 -0.605564819

H 7.729140309 0.706089772 1.157693365
H 7.935127777 -0.801040332 0.270569599
H 1.807991454 -5.453141152 0.475240207
H 2.078334422 -5.222669869 -1.263318647
H 3.441310972 -5.440829644 -0.186177511
H -1.748764703 -4.922599503 -1.685482759
H -1.758370072 -5.435867731 0.013777185
H -3.26866902 -5.254122644 -0.883716565
H -7.875462183 -0.563433039 -0.192137121
H -7.527687118 -0.847737261 1.515929206
H -7.798984785 0.790592282 0.936593928
H -2.072348911 5.419140325 0.610327098
H -1.86542612 5.352161671 -1.150747876
H -3.474005433 5.443448701 -0.449440732
H 2.321406496 4.381472908 -0.060649439

28H (1.0 nN, C_1) H-substituent on the 6-position

N 4.045763395 -1.406602612 0.066214779
N 0.103919319 -3.453123219 -0.040439855
N -3.883148478 -1.339925517 0.009844447
N -4.029671006 1.448865692 -0.053712908
N -0.08506671 3.46686336 0.137994773
N 3.923450554 1.305366166 -0.057376868
C 6.042782045 0.0339495 -0.16785878
C 5.415095377 -1.185404612 -0.079033322
C 6.027633376 -2.50851954 -0.107649844
C 5.055213428 -3.440269027 0.01026997
C 3.751184933 -2.771943945 0.10889922
C 2.531418019 -3.392697239 0.171583268
C 1.238845991 -2.735319905 0.272906124
C 0.792180709 -1.474043764 0.698264235
C -0.61875469 -1.457259066 0.624271752
C -1.054401099 -2.701051662 0.152316673
C -2.338710987 -3.321551174 -0.054284764
C -3.582483851 -2.724238609 -0.05194038
C -4.84324316 -3.466898473 -0.122632305
C -5.848434103 -2.561109066 -0.095911221
C -5.226909468 -1.238210009 -0.028954115
C -6.008688517 -0.017344112 -0.028732596
C -5.405978822 1.211864104 -0.035658561
C -6.031806441 2.528683654 -0.016145333
C -5.062332756 3.470790366 -0.00272868
C -3.747847726 2.818426161 -0.030222061
C -2.530889887 3.448691505 -0.02501706
C -1.248030398 2.789027671 -0.167743645
C -0.848985359 1.529877967 -0.648950003

C 0.558715411 1.468543658 -0.611983184<br>C 1.039871856 2.682174458 -0.10895283<br>C 2.337786764 3.231240693 0.100904413<br>C 3.582436493 2.662875453 0.085033472<br>C 4.809114371 3.45312079 0.188095829<br>C 5.849946569 2.590681708 0.075026205<br>C 5.270401497 1.249600048 -0.066122081<br>H 3.4113584 -0.610813046 0.040675382<br>H 0.121350569 -4.387218254 -0.409477444<br>H -3.384869639 0.664563046 -0.010097159<br>H -0.050406743 4.391135055 0.535245864<br>H 7.088279198 -2.685859932 -0.189983167<br>H 5.204464055 -4.506799401 0.032578252<br>H 1.415428221 -0.67046853 1.059357429<br>H -1.271082559 -0.648215324 0.906809871<br>H -4.963473869 -4.536889196 -0.158935169<br>H -6.907138549 -2.763456778 -0.121557656<br>H -7.096783126 2.696658289 -0.010803601<br>H -5.219011011 4.536569016 0.006281726<br>H -1.504053892 0.759332064 -1.02527031<br>H 1.180727725 0.651264531 -0.936578197<br>H 4.841902023 4.525835638 0.314640581<br>H 6.904308493 2.817598404 0.110610267<br>C 7.535312959 0.110369085 -0.482446708<br>C 2.460488645 -4.905065151 0.093367772<br>C -2.250440492 -4.818266179 -0.297384498<br>C -7.523418058 -0.198279366 -0.034879496<br>C -2.456066033 4.950928892 0.146538003<br>H 7.736517251 1.01642084 -1.060967074<br>H 8.253290799 0.111158766 0.362673517<br>H 7.820279689 -0.72963594 -1.122448212<br>H 1.789732706 -5.300669404 0.863912022<br>H 2.089213654 -5.243010046 -0.884545938<br>H 3.430323636 -5.374812317 0.238834149<br>H -1.730951172 -5.018155782 -1.243419829<br>H -1.694730062 -5.310917514 0.507555461<br>H -3.222327424 -5.293058228 -0.371305034<br>H -7.823361106 -0.77214403 -0.919728599<br>H -7.83325686 -0.781154591 0.840519886<br>H -8.148377157 0.70895643 -0.036682233<br>H -2.018892765 5.218847919 1.118608915<br>H -1.837515035 5.403695458 -0.636727578<br>H -3.434186775 5.424652867 0.106658158<br>H 2.341680073 4.305213837 0.288643225
**28H** (0 nN, C_1) *i*Pr-substituent on the 6-position

N 3.853777015 -1.389475881 -0.104740014
N 0.042287008 -3.558442816 0.355767767
N -3.844753402 -1.477847602 -0.115388407
N -3.93141455 1.375995211 -0.200725359
N -0.093547083 3.458434727 0.455267284
N 3.761780807 1.453279865 -0.215103295
C 5.760533292 0.057998915 -0.696465445
C 5.180192099 -1.157720588 -0.467491205
C 5.825324007 -2.466487092 -0.466957099
C 4.921625976 -3.395140644 -0.086766265
C 3.627418027 -2.74628876 0.136303817
C 2.458088958 -3.38875653 0.432871939
C 1.156252428 -2.760666141 0.478924172
C 0.683610075 -1.442272072 0.598589811
C -0.72356255 -1.475116213 0.521850101
C -1.127967876 -2.805601982 0.356423286
C -2.387650006 -3.471099361 0.206816117
C -3.606198496 -2.866351965 -0.000948695
C -4.870632654 -3.57860669 -0.169862262
C -5.82879338 -2.644035085 -0.3807768
C -5.162494179 -1.342946779 -0.33731604
C -5.885723743 -0.103940864 -0.477022823
C -5.292537348 1.12278808 -0.373776248
C -5.942640785 2.428898321 -0.349385618
C -5.00808798 3.375945183 -0.118399919
C -3.691957351 2.741818061 -0.02784428
C -2.50168727 3.385672788 0.165162598
C -1.199633775 2.778092416 0.008695762
C -0.715627123 1.639852379 -0.662554432
C 0.69133766 1.67067371 -0.611739057
C 1.086312341 2.819572367 0.094862048
C 2.358790732 3.437449293 0.323061879
C 3.567519355 2.826122164 0.055994602
C 4.851423408 3.514836076 -0.063484894
C 5.769630162 2.585158377 -0.427274371
C 5.069745717 1.304191629 -0.482163383
H 3.207310489 -0.611965765 -0.033160932
H 0.081734856 -4.549798031 0.195738227
H -3.282901348 0.601786998 -0.101413152
H -0.137856233 4.304862349 0.990614513
H 6.860413059 -2.642548627 -0.709338493
H 5.102169165 -4.452496875 0.012254398
H 1.279115582 -0.559583489 0.771346628
H -1.395003715 -0.639141245 0.604524414
H -5.021009963 -4.645376913 -0.138666239
H -6.880972803 -2.81209525 -0.546929217
H -6.999496506 2.594318918 -0.477739129

H -5.185428784 4.436210047 -0.050226038
H -1.316405326 0.910131519 -1.182559247
H 1.37609069 0.973537729 -1.065096582
H 5.032815288 4.571389452 0.052641445
H 6.818916598 2.742925619 -0.620947504
C 7.215304256 0.148106681 -1.095020894
C 2.459136936 -4.890834522 0.633433843
C -2.30978093 -4.984510295 0.256062568
C -7.377233314 -0.216226215 -0.692288147
C -2.483023935 4.854067692 0.532121174
C 2.388291827 4.890255639 0.809596113
H 7.340362358 0.823090304 -1.947736147
H 7.835608112 0.534455604 -0.277347077
H 7.61827252 -0.820611153 -1.389313581
H 1.817451167 -5.163787898 1.478361548
H 2.096589202 -5.427616138 -0.254822855
H 3.452867506 -5.274356363 0.856592449
H -1.790293969 -5.37619225 -0.627858976
H -1.762138707 -5.310897412 1.146475266
H -3.289059838 -5.451432749 0.286928668
H -7.597319168 -0.813653855 -1.583644656
H -7.86744699 -0.703924593 0.1580356
H -7.847139096 0.756348183 -0.831546492
H -2.077076876 5.004029417 1.543021351
H -1.864073815 5.431123046 -0.1655692
H -3.477680393 5.293975258 0.537629041
H 3.438590334 5.13266278 0.964137619
C 1.854075952 5.876517319 -0.245785479
H 0.800407864 5.69496192 -0.478517291
H 1.951942415 6.90430196 0.118804603
H 2.420580838 5.78341671 -1.176350452
C 1.727505248 5.113673344 2.194837401
H 2.253553282 5.915167216 2.720922243
H 0.682262973 5.438998063 2.128737198
H 1.773055274 4.213772673 2.813373138

28H (1.0 nN, C_1) *i*Pr-substituent on the 6-position

N 3.95628218 -1.364692417 0.029946264
N 0.037247735 -3.491385178 0.057868198
N -3.93880064 -1.434245318 -0.033875139
N -4.023865969 1.342323206 -0.114742733
N -0.090503881 3.414126809 0.338460069
N 3.879646831 1.417482919 -0.046726989
C 5.948655802 0.060329438 -0.323453384
C 5.31907081 -1.150151465 -0.188927658
C 5.927285159 -2.474460228 -0.220166704

C 4.963748066 -3.400139398 -0.022158997
C 3.665979961 -2.729365845 0.122719941
C 2.457691586 -3.362926368 0.247549062
C 1.154466499 -2.72747977 0.318390043
C 0.679449045 -1.4464959 0.645389167
C -0.72999171 -1.466636469 0.567263616
C -1.136790115 -2.752067748 0.188445122
C -2.403064836 -3.406679304 -0.00125778
C -3.648962742 -2.818416614 -0.053539997
C -4.909756346 -3.555185417 -0.147243204
C -5.909104739 -2.640722123 -0.174072609
C -5.27832333 -1.322511374 -0.111537306
C -6.036301334 -0.088436381 -0.137925513
C -5.406457395 1.126953521 -0.130208157
C -6.010910632 2.452484437 -0.106652585
C -5.028485694 3.377748561 -0.046359327
C -3.721099593 2.705923102 -0.046687284
C -2.502753516 3.330179574 0.029918685
C -1.192315822 2.709408767 -0.092595648
C -0.689639913 1.538859474 -0.687533075
C 0.719223603 1.572150216 -0.603124589
C 1.101958358 2.755867407 0.048230956
C 2.371446764 3.386862752 0.305933172
C 3.613262262 2.79255381 0.155706701
C 4.891120965 3.512547692 0.141816059
C 5.868048639 2.59982532 -0.084239539
C 5.216219026 1.297462362 -0.173987032
H 3.324336247 -0.569600821 0.06056108
H 0.075802924 -4.447041834 -0.249330619
H -3.393289332 0.545770286 -0.075069464
H -0.147745191 4.284649842 0.832101796
H 6.980749253 -2.658490975 -0.357610616
H 5.112757432 -4.466146142 0.012207411
H 1.28047793 -0.603827811 0.94957241
H -1.399991518 -0.653692453 0.788982379
H -5.034207372 -4.625317854 -0.171194231
H -6.968069884 -2.834926384 -0.234081078
H -7.072880827 2.637424416 -0.129146138
H -5.172696316 4.444620213 -0.022035243
H -1.278304144 0.773902261 -1.169783657
H 1.409806913 0.84452531 -0.996524361
H 5.039658369 4.575556376 0.240356618
H 6.929002934 2.780942358 -0.160774501
C 7.425838015 0.115237544 -0.708156744
C 2.41488204 -4.879607583 0.260117296
C -2.296905874 -4.912017703 -0.161680817
C -7.553160986 -0.245296118 -0.170171226

C -2.450083517 4.827229585 0.271185588
C 2.3498498 4.867742309 0.712622001
H 7.5911469 0.956731987 -1.387608079
H 8.174749245 0.215105585 0.104233288
H 7.69831504 -0.784487448 -1.265276336
H 1.73208956 -5.236832339 1.038634502
H 2.075051863 -5.287859451 -0.702345248
H 3.387150605 -5.320874127 0.465766452
H -1.770635377 -5.161154624 -1.09203671
H -1.74069408 -5.351950257 0.672977808
H -3.265042152 -5.399011014 -0.208102457
H -7.847523735 -0.804268757 -1.066536388
H -7.882212277 -0.836144432 0.692732997
H -8.167400857 0.668877565 -0.166500552
H -2.079789435 5.056088279 1.281317607
H -1.784201978 5.316947679 -0.448672818
H -3.424928784 5.300840268 0.189647925
H 3.38319896 5.141039823 0.911167821
C 1.865074507 5.78179536 -0.428576753
H 0.829844853 5.568917576 -0.711636415
H 1.927793207 6.830445262 -0.11977704
H 2.487990181 5.644512675 -1.316683211
C 1.606968466 5.159491333 2.045055664
H 2.13870428 5.940860783 2.594632272
H 0.590410908 5.543814013 1.894130364
H 1.554526351 4.273988669 2.683670969

28H (0 nN, C_1) Ph-substituent on the 6-position

N 3.92928212 -1.417968728 0.175470888
N 0.071691881 -3.534657822 -0.112945295
N -3.842679852 -1.432083966 -0.137117089
N -3.953343577 1.426764814 -0.055999498
N -0.082825698 3.530371867 0.197902227
N 3.828036786 1.421720767 0.059734846
C 5.903103156 0.056302616 0.042004173
C 5.299551406 -1.168481022 0.11823558
C 5.942952319 -2.477416154 0.162468544
C 4.987908547 -3.428669191 0.233977694
C 3.669782656 -2.790623665 0.235706791
C 2.468554744 -3.441200255 0.246883241
C 1.172974253 -2.796839513 0.253340517
C 0.691335889 -1.530021797 0.624720946
C -0.711262344 -1.535601234 0.473592543
C -1.100607809 -2.794984186 0.000145115
C -2.355491439 -3.42412046 -0.292354743
C -3.587434541 -2.813010431 -0.304451032

C -4.860681147 -3.513957724 -0.460292363
C -5.840510161 -2.583806005 -0.366532287
C -5.17815598 -1.293232997 -0.176343186
C -5.918862868 -0.062171988 -0.067539238
C -5.322444444 1.166228487 -0.01642161
C -5.970361486 2.470573426 0.078374056
C -5.018999893 3.427882368 0.103896311
C -3.699155179 2.79986024 0.019142363
C -2.499328251 3.452410824 0.031751926
C -1.209703152 2.817220816 -0.125746615
C -0.761147143 1.584431227 -0.636468362
C 0.648480917 1.584503753 -0.603781216
C 1.073388137 2.812223839 -0.079047339
C 2.349324505 3.429704683 0.130213882
C 3.576427817 2.807739044 0.117878934
C 4.847382119 3.530606399 0.123201409
C 5.828934834 2.59858621 0.06571498
C 5.166798452 1.292527353 0.043543539
H 3.27195192 -0.646298582 0.126881449
H 0.121215897 -4.476660434 -0.458538334
H -3.29263264 0.657683695 -0.067488686
H -0.080978426 4.447083768 0.613799771
H 7.007431325 -2.642330603 0.144537461
H 5.157183635 -4.491193518 0.281298912
H 1.2802413 -0.712497719 1.011167995
H -1.39224639 -0.736782252 0.710158568
H -5.003230289 -4.573535262 -0.596389466
H -6.904891709 -2.746342555 -0.426419702
H -7.035293077 2.628846989 0.117559414
H -5.191963569 4.489173312 0.164472651
H -1.380797812 0.798876576 -1.039852754
H 1.307300625 0.803634831 -0.945829417
H 4.953994855 4.603826469 0.130253931
H 6.893725489 2.767515713 0.040155698
C 7.408832875 0.167616185 -0.022614646
C 2.429443346 -4.955278758 0.208887785
C -2.259049192 -4.905762426 -0.597787251
C -7.424808163 -0.184469493 -0.030371575
C -2.450625707 4.951426943 0.242330886
H 7.721031074 0.73474067 -0.906188825
H 7.806991174 0.686228476 0.856987871
H 7.891916077 -0.806963445 -0.075517789
H 1.713281462 -5.341410975 0.942495466
H 2.133758124 -5.32937615 -0.781683488
H 3.394813112 -5.401238803 0.437034877
H -1.667092294 -5.070129028 -1.506905802
H -1.775097328 -5.442675242 0.225765434

H -3.22910936 -5.361628916 -0.766944003<br>H -7.812007419 -0.60562472 -0.965338066<br>H -7.744253937 -0.844711561 0.783089274<br>H -7.911801804 0.77775265 0.120798603<br>H -2.063223819 5.204258928 1.239108834<br>H -1.798993423 5.428702572 -0.498706057<br>H -3.430685016 5.415643365 0.16019376<br>C 2.319111515 4.904292164 0.382347667<br>C 2.856848745 5.436273491 1.563169867<br>C 1.73602409 5.777569754 -0.551630241<br>C 2.819457101 6.810594209 1.802903139<br>H 3.299002006 4.761683615 2.288180558<br>C 1.704197944 7.150705352 -0.312659039<br>H 1.324824443 5.37055716 -1.46992765<br>C 2.24456041 7.670293993 0.86626017<br>H 3.235226739 7.206830989 2.722067156<br>H 1.265557826 7.814976865 -1.048411403<br>H 2.217011092 8.737870408 1.051927495
**28H** (1.0 nN, C_1) Ph-substituent on the 6-position<br><br>N 4.01546958 -1.413445612 0.144062685<br>N 0.068096758 -3.454878089 -0.163686281<br>N -3.914068036 -1.355824945 -0.089959612<br>N -4.028785737 1.423813485 -0.063201589<br>N -0.084478263 3.441156405 0.260955842<br>N 3.900615423 1.341253835 0.108519099<br>C 6.017899696 0.02296384 0.118316782<br>C 5.395458969 -1.197384285 0.130307517<br>C 6.002088848 -2.52240364 0.122041119<br>C 5.018895403 -3.449826938 0.115202086<br>C 3.71307802 -2.777141751 0.127134106<br>C 2.489458933 -3.394206565 0.101195768<br>C 1.195387878 -2.738600397 0.178576957<br>C 0.736663991 -1.484458404 0.616978743<br>C -0.672331575 -1.473945719 0.525877717<br>C -1.096641613 -2.712162679 0.027788231<br>C -2.37432286 -3.332469843 -0.206891827<br>C -3.61936301 -2.736832434 -0.195893537<br>C -4.881566847 -3.471611045 -0.298647454<br>C -5.883899324 -2.562138844 -0.246544998<br>C -5.257006502 -1.24611287 -0.131179039<br>C -6.026438712 -0.018494586 -0.089844269<br>C -5.408247859 1.202038464 -0.054204155<br>C -6.019378963 2.523880858 0.0137569<br>C -5.039862887 3.453530612 0.064698539<br>C -3.732110832 2.787448229 0.016124581

C -2.511045417 3.406362996 0.058114317
C -1.221898725 2.756553533 -0.098001696
C -0.782979724 1.542274817 -0.657826607
C 0.627418463 1.523950953 -0.620014908
C 1.069166988 2.720876637 -0.04087468
C 2.355920884 3.319261335 0.192102979
C 3.598327928 2.71751898 0.1781398
C 4.850794002 3.475441609 0.214625476
C 5.862794727 2.575088142 0.169649699
C 5.24710013 1.247923353 0.121618455
H 3.384551845 -0.615852493 0.113669968
H 0.093629812 -4.384586035 -0.542879945
H -3.393150056 0.63046099 -0.052762565
H -0.073875129 4.341791664 0.710588356
H 7.064480695 -2.706158134 0.119331798
H 5.16208748 -4.517271305 0.110695877
H 1.349525235 -0.682400485 0.998772231
H -1.332070106 -0.673362815 0.814887117
H -5.005185701 -4.539419463 -0.372673223
H -6.943587207 -2.757931317 -0.285515686
H -7.082399499 2.703584337 0.02419616
H -5.185086841 4.519563758 0.116123418
H -1.413871806 0.77825544 -1.085352476
H 1.274560501 0.747362875 -0.993073768
H 4.927316197 4.551018627 0.232571854
H 6.921327721 2.781178064 0.164779301
C 7.53424588 0.183461247 0.116401172
C 2.418465426 -4.902136935 -0.036769441
C -2.281768776 -4.821159564 -0.491403448
C -7.543063738 -0.178729036 -0.100332361
C -2.432396757 4.901507103 0.2936187
H 7.846135816 0.760862242 -0.761961771
H 7.846949878 0.755434047 0.998054286
H 8.145695276 -0.73362808 0.11467451
H 1.744528877 -5.330413674 0.713815828
H 2.050456476 -5.197946544 -1.02949858
H 3.387251075 -5.378896908 0.08925376
H -1.748937269 -4.994442 -1.435162837
H -1.737501997 -5.336913344 0.307023065
H -3.25316177 -5.292685871 -0.591502529
H -7.853114361 -0.711213429 -1.007404881
H -7.858991555 -0.793250809 0.75092683
H -8.153945573 0.737860276 -0.062075952
H -2.046714945 5.127997154 1.297492501
H -1.764971788 5.377910813 -0.433675079
H -3.40186621 5.387928863 0.215981621
C 2.31715763 4.789521292 0.480738944

C 2.821388217 5.293324155 1.688142327<br>C 1.755182633 5.683525375 -0.446136987<br>C 2.771775613 6.661231713 1.961116513<br>H 3.248626679 4.602619912 2.406876481<br>C 1.712074971 7.050276166 -0.174598929<br>H 1.367802399 5.297408498 -1.383588943<br>C 2.218798129 7.542106847 1.030991233<br>H 3.161515303 7.035830295 2.900540018<br>H 1.290379309 7.731164661 -0.905032914<br>H 2.182028461 8.604583327 1.242423434
**$28M_{1a}$** (0 nN, C_1) H-substituent on the 6-position<br><br>N -3.34628945 1.547621348 0.598700902<br>N 0.575540709 3.325383342 -0.535401906<br>N 3.884781314 0.663031025 -0.393074095<br>N 3.095084187 -1.419907554 0.959919896<br>N -1.008107454 -2.004000502 -0.013054608<br>N -3.658083915 -0.982977058 -0.34475591<br>C -5.404704271 0.204234476 0.897381794<br>C -4.577998029 1.322199398 1.199001239<br>C -4.880939358 2.484153565 1.937406451<br>C -3.843479257 3.396856565 1.747249418<br>C -2.881314027 2.803032316 0.893622842<br>C -1.667258055 3.397086816 0.400943956<br>C -0.601351507 2.697574748 -0.107937327<br>C -0.332743049 1.272036964 -0.244834526<br>C 0.951375851 1.09983437 -0.660637732<br>C 1.580791047 2.398958885 -0.82729586<br>C 2.86492366 2.750145245 -1.169988341<br>C 3.968710503 1.833419533 -1.041226865<br>C 5.361046398 2.101177596 -1.400457352<br>C 6.10481322 1.063391459 -0.917491333<br>C 5.182333449 0.15552057 -0.253312645<br>C 5.464021828 -0.941493135 0.547752795<br>C 4.429735531 -1.669788636 1.206915829<br>C 4.469014807 -2.65591472 2.217135347<br>C 3.146910172 -2.95697512 2.57579527<br>C 2.294727161 -2.185714887 1.755445811<br>C 0.8538789 -2.156523855 1.595705414<br>C 0.333595432 -2.179094655 0.329948644<br>C 0.995842362 -2.581051908 -0.914315802<br>C 0.060733103 -2.701913307 -1.88888504<br>C -1.239867098 -2.334314639 -1.344983004<br>C -2.46343775 -2.3731331 -1.952570505<br>C -3.659840405 -1.914586653 -1.319388616<br>C -5.011661596 -2.387374013 -1.571612017

C -5.826500136 -1.746774362 -0.681381621<br>C -4.989276364 -0.821710122 0.065361184<br>H -2.964190023 0.838003788 -0.023429384<br>H 0.748508286 4.310077809 -0.431621371<br>H 2.852435701 -0.600570019 0.386112235<br>H -1.756378225 -1.660596313 0.574535356<br>H -5.768998395 2.635440925 2.530315095<br>H -3.768015619 4.378698479 2.184654244<br>H -1.034115651 0.475225803 -0.056768728<br>H 1.434021318 0.167008367 -0.897258793<br>H 5.727490769 2.961771575 -1.937867875<br>H 7.174582964 0.944583036 -0.99892604<br>H 5.363186413 -3.09288277 2.634107573<br>H 2.824650072 -3.68843657 3.29967067<br>H 2.049799598 -2.799686248 -0.984247321<br>H 0.213307522 -3.02260826 -2.907791923<br>H -5.286010523 -3.127774908 -2.307682141<br>H -6.896508245 -1.854030383 -0.58652485<br>C -6.819184865 0.245899516 1.417894777<br>C -1.545615007 4.902554914 0.489747524<br>C 3.161939765 4.194284791 -1.49764816<br>C 6.881428508 -1.367328825 0.82321576<br>C -0.026498242 -2.090541574 2.813817118<br>H -6.82134171 0.455230203 2.492183052<br>H -7.397903741 1.036555131 0.928768668<br>H -7.327418507 -0.705618139 1.265310802<br>H -0.838642319 5.212071893 1.270315476<br>H -1.197077846 5.31833935 -0.463532236<br>H -2.50792182 5.3673172 0.701614353<br>H 3.421195961 4.774807216 -0.602052093<br>H 4.003760833 4.276229901 -2.187736085<br>H 2.303689593 4.673019724 -1.979800136<br>H 7.151760294 -1.14744868 1.862159259<br>H 6.995110046 -2.445596589 0.676715399<br>H 7.592097027 -0.856505973 0.174550236<br>H -0.235575587 -1.051612042 3.099535995<br>H -0.982997756 -2.595320818 2.646927515<br>H 0.467622872 -2.562752745 3.667335508<br>H -2.528655545 -2.79861117 -2.946465434
**28M$_{1a}$** (1.0 nN, C_1) H-substituent on the 6-position<br><br>N -3.57098558 1.663897879 0.473501276<br>N 0.552251712 3.302104839 -0.336927899<br>N 4.007187586 0.645361132 -0.293893622<br>N 3.334978135 -1.564912859 0.828059765<br>N -0.913888058 -2.067156461 0.149287593

N -3.827380426 -0.878272963 -0.277733943
C -5.697747004 0.402726124 0.691997273
C -4.880219801 1.547179016 0.932864503
C -5.208640412 2.788346899 1.514592166
C -4.106913441 3.631996189 1.378154938
C -3.074757371 2.918293516 0.714269144
C -1.776880923 3.429108466 0.339031795
C -0.669486586 2.692790712 -0.015416543
C -0.393628588 1.263381973 -0.098335318
C 0.921879205 1.076954884 -0.404740125
C 1.573647234 2.3706373 -0.555259219
C 2.862713712 2.750683236 -0.865921194
C 4.014142002 1.878560958 -0.814009515
C 5.388243723 2.248877454 -1.171775165
C 6.192724575 1.20406202 -0.820868265
C 5.337225239 0.183355883 -0.241707566
C 5.693003338 -0.992898252 0.403314068
C 4.683918051 -1.824136159 0.998490944
C 4.76466376 -2.90370113 1.90397328
C 3.457047169 -3.244590414 2.287213708
C 2.569131115 -2.400162813 1.587211967
C 1.111953925 -2.328047613 1.558418151
C 0.460664656 -2.272575829 0.358191123
C 0.99650825 -2.615604438 -0.961479796
C -0.026954125 -2.698149541 -1.843070234
C -1.274803941 -2.350718648 -1.168922804
C -2.526083625 -2.390513655 -1.710558252
C -3.746663308 -1.928100475 -1.11136609
C -5.066513892 -2.49548036 -1.351262907
C -5.95072386 -1.773283514 -0.601435111
C -5.191561401 -0.714838479 0.042033687
H -3.149820499 0.862050836 -0.002132614
H 0.716249176 4.290376725 -0.262755814
H 3.076661324 -0.686761903 0.346748678
H -1.587063159 -1.78164186 0.843637624
H -6.153449117 3.036259806 1.971894014
H -4.038330128 4.646517268 1.73369495
H -1.120279746 0.477415372 0.042602844
H 1.412678354 0.133402195 -0.573484762
H 5.706456417 3.175707743 -1.621626557
H 7.26440844 1.142501286 -0.936748967
H 5.674588828 -3.373216289 2.244618742
H 3.168182312 -4.040339168 2.95540409
H 2.039797106 -2.824121048 -1.140325974
H 0.025236609 -2.973098981 -2.885379242
H -5.271951531 -3.344555625 -1.98548059
H -7.019543102 -1.913171767 -0.536893703

C -7.149380434 0.434795742 1.153051627
C -1.61230488 4.935708506 0.360431924
C 3.094616585 4.220617784 -1.141899035
C 7.131578126 -1.434008638 0.618672479
C 0.36783883 -2.301522999 2.867182331
H -7.206608717 0.92951176 2.127140771
H -7.87814975 0.948101378 0.495526429
H -7.510981422 -0.586066233 1.290024414
H -0.974093742 5.266564794 1.190015786
H -1.15779728 5.285758047 -0.574526228
H -2.570992465 5.443474752 0.448683979
H 3.14775293 4.806519146 -0.213596777
H 4.027338813 4.387843592 -1.678850007
H 2.288846779 4.635492937 -1.757654918
H 7.371186067 -1.35983154 1.686268852
H 7.229906856 -2.490025509 0.346555107
H 7.919022441 -0.885702936 0.076829232
H 0.270544136 -1.278510748 3.252035224
H -0.635613648 -2.727364602 2.77023916
H 0.909692869 -2.876531276 3.62331791
H -2.604704734 -2.817270596 -2.705100948

28M$_{1a}$ (0 nN, C$_1$) *i*Pr-substituent on the 6-position

N -3.314376198 1.542537352 0.565047854
N 0.588872123 3.323652738 -0.617631604
N 3.88894534 0.664237307 -0.365318353
N 3.060368508 -1.404289086 0.997006858
N -1.019666447 -2.013744879 -0.063232177
N -3.617024883 -0.982395061 -0.356832039
C -5.324653446 0.159090621 0.978159612
C -4.523470926 1.308795441 1.207547264
C -4.827764658 2.484975655 1.92528876
C -3.81348281 3.409746008 1.684520695
C -2.861322327 2.809347803 0.821569282
C -1.658742109 3.402516343 0.304023239
C -0.589682685 2.697888036 -0.193554324
C -0.317745459 1.270876323 -0.312823224
C 0.971957113 1.097801594 -0.710962801
C 1.601288608 2.395211402 -0.881096265
C 2.892843662 2.741644108 -1.197868309
C 3.991680866 1.824896834 -1.030262478
C 5.391457802 2.084140871 -1.359477947
C 6.120915129 1.051312871 -0.842782377
C 5.180389047 0.156244261 -0.18872956
C 5.439776878 -0.932381302 0.634140742
C 4.390736636 -1.651669612 1.27426101

C 4.407638593 -2.635096376 2.289297043<br>C 3.079913289 -2.936257338 2.619191398<br>C 2.243210756 -2.17097542 1.774402732<br>C 0.808790304 -2.160146912 1.580935166<br>C 0.314456522 -2.164269664 0.301891504<br>C 1.009866122 -2.515353884 -0.935926531<br>C 0.10013937 -2.623991477 -1.93618085<br>C -1.22745933 -2.308854247 -1.415382139<br>C -2.457494876 -2.361615466 -2.023795517<br>C -3.634015327 -1.947536711 -1.297558767<br>C -4.971060386 -2.508406695 -1.419515764<br>C -5.752912612 -1.874969399 -0.493202512<br>C -4.919162474 -0.876563908 0.149391447<br>H -2.942167359 0.825257724 -0.055883369<br>H 0.759982246 4.309049589 -0.516582792<br>H 2.82873706 -0.589568953 0.41341952<br>H -1.778009991 -1.682504735 0.517435098<br>H -5.700836768 2.634814236 2.540473584<br>H -3.742209247 4.402734875 2.096772708<br>H -1.01921974 0.473065801 -0.127966892<br>H 1.459757903 0.164284831 -0.933114825<br>H 5.77315125 2.935611561 -1.900871947<br>H 7.192043089 0.929067064 -0.896581844<br>H 5.292923491 -3.07021601 2.726753586<br>H 2.742552182 -3.66627211 3.337616222<br>H 2.069130804 -2.711148119 -0.993032321<br>H 0.308179363 -2.911419963 -2.953198121<br>H -5.263008657 -3.304516077 -2.087567798<br>H -6.802489716 -2.045388556 -0.306924176<br>C -6.707884152 0.161596235 1.578315078<br>C -1.543370381 4.909488526 0.369823575<br>C 3.200628365 4.181003724 -1.535742487<br>C 6.850233203 -1.354955646 0.949215076<br>C -0.102578764 -2.149919204 2.77795579<br>C -2.645812362 -2.862309577 -3.451101228<br>H -6.660433938 0.418479912 2.640869777<br>H -7.34938162 0.902445265 1.089434187<br>H -7.179660975 -0.81683241 1.494298169<br>H -0.843046658 5.234896356 1.150167161<br>H -1.190497951 5.311739442 -0.587562777<br>H -2.509156749 5.373233321 0.568524486<br>H 3.448520287 4.769061784 -0.641940451<br>H 4.053830393 4.252041325 -2.21305724<br>H 2.352111118 4.658274479 -2.036056029<br>H 7.095474732 -1.123306788 1.991770923<br>H 6.967855783 -2.434662046 0.817289201<br>H 7.576386388 -0.85101621 0.312465477

H -0.355235335 -1.12562228 3.080904949<br>H -1.037072929 -2.682527136 2.575121674<br>H 0.384943858 -2.624234134 3.634135817<br>H -3.724222181 -2.820217018 -3.644751721<br>C -1.993609221 -1.928989064 -4.489140327<br>H -2.241036713 -2.259047115 -5.503592114<br>H -2.350755139 -0.903538536 -4.360536214<br>H -0.904377457 -1.916503935 -4.395363641<br>C -2.212083287 -4.325624866 -3.647149049<br>H -1.136961892 -4.453018559 -3.493275241<br>H -2.728778535 -4.978124888 -2.936986896<br>H -2.449840748 -4.659272174 -4.662754803<br>H 2.182028461 8.604583327 1.242423434
**28M$_{1a}$** (1.0 nN, C$_1$) *i*Pr-substituent on the 6-position<br><br>N -3.523097902 1.65127804 0.41579415<br>N 0.577157905 3.302483479 -0.446431578<br>N 4.019773832 0.64388783 -0.273383151<br>N 3.306438851 -1.543231572 0.87908<br>N -0.923628075 -2.08869853 0.080846426<br>N -3.761449373 -0.893775649 -0.309295237<br>C -5.611163312 0.351995285 0.744354222<br>C -4.819308479 1.524687941 0.909571606<br>C -5.156602494 2.777250095 1.463661477<br>C -4.073508266 3.634375089 1.279931627<br>C -3.043011767 2.917983502 0.614541186<br>C -1.755709693 3.433557695 0.214329472<br>C -0.642236678 2.695080255 -0.115722028<br>C -0.359377975 1.264498702 -0.156581008<br>C 0.959126159 1.078148801 -0.447477524<br>C 1.606077577 2.369472224 -0.625919086<br>C 2.899769951 2.739968621 -0.923529405<br>C 4.04651178 1.865224548 -0.822222267<br>C 5.429280309 2.221809573 -1.151748884<br>C 6.219699241 1.180882048 -0.756116986<br>C 5.344314489 0.178221984 -0.177806536<br>C 5.677255919 -0.988125661 0.500236709<br>C 4.652053211 -1.803696415 1.083441213<br>C 4.707394992 -2.877024831 1.999756117<br>C 3.391578959 -3.214481656 2.350548725<br>C 2.519830719 -2.375097973 1.620448989<br>C 1.064966465 -2.327821731 1.546311216<br>C 0.446385977 -2.249447945 0.327470624<br>C 1.036842651 -2.510335708 -0.984568921<br>C 0.050162235 -2.581808433 -1.907761271<br>C -1.241150708 -2.324759643 -1.267650249

C -2.495644312 -2.39185194 -1.811875506<br>C -3.688873762 -1.984121839 -1.088846714<br>C -4.98767426 -2.641014628 -1.163817053<br>C -5.847384739 -1.914774095 -0.388082865<br>C -5.10169423 -0.779369242 0.118080837<br>H -3.104755605 0.847665578 -0.061240544<br>H 0.737165503 4.292549773 -0.38776021<br>H 3.062582774 -0.671089659 0.381180474<br>H -1.624913182 -1.842087417 0.761956904<br>H -6.093992688 3.021864986 1.937636541<br>H -4.011858745 4.65865523 1.607862626<br>H -1.081452149 0.476976419 0.000379724<br>H 1.455931499 0.134023152 -0.589221879<br>H 5.763233511 3.138109453 -1.611792939<br>H 7.29381071 1.112626006 -0.841842667<br>H 5.607851558 -3.345282668 2.366428071<br>H 3.085258739 -4.007021534 3.014740543<br>H 2.092149745 -2.671371044 -1.140933328<br>H 0.179473549 -2.797314203 -2.954603064<br>H -5.200375803 -3.550029083 -1.706320713<br>H -6.894409015 -2.113780916 -0.214627423<br>C -7.043818755 0.364970602 1.261819571<br>C -1.607434664 4.941413771 0.192859831<br>C 3.145093971 4.199532858 -1.238329506<br>C 7.110381235 -1.423956344 0.759890857<br>C 0.275733763 -2.374842764 2.827971356<br>C -2.727419514 -2.870465363 -3.246788858<br>H -7.087749072 0.931214049 2.19622879<br>H -7.813267156 0.797905711 0.592193254<br>H -7.360242841 -0.654972784 1.488699214<br>H -0.985297511 5.305032444 1.021099717<br>H -1.143708672 5.268968161 -0.745614289<br>H -2.573605689 5.439766095 0.252984261<br>H 3.225036936 4.806785495 -0.326084185<br>H 4.069196757 4.33910572 -1.798359101<br>H 2.333935224 4.610323559 -1.849191939<br>H 7.335749501 -1.296223075 1.82555095<br>H 7.208924803 -2.492320557 0.542236985<br>H 7.906611169 -0.905573605 0.201059324<br>H 0.104993324 -1.368896177 3.232023565<br>H -0.698224609 -2.853209904 2.683474899<br>H 0.820534973 -2.936721592 3.591603508<br>H -3.81541234 -2.885709317 -3.380029907<br>C -2.195920094 -1.867628939 -4.28963555<br>H -2.481401354 -2.183731573 -5.298510514<br>H -2.611497018 -0.872785799 -4.107105769<br>H -1.106531413 -1.783574003 -4.258036962

C -2.233985657 -4.302963376 -3.514536141<br>H -1.14726824 -4.387004692 -3.436490459<br>H -2.672025877 -4.999829198 -2.793683249<br>H -2.524274014 -4.618683626 -4.522141273<br>H 2.182028461 8.604583327 1.242423434
**$28M_{1a}$** (0 nN, C_1) Ph-substituent on the 6-position<br><br>N -3.368513753 1.585725929 0.335918827<br>N 0.554295295 3.363629991 -0.786170937<br>N 3.834704787 0.673360219 -0.660830294<br>N 3.003809492 -1.438162102 0.632556277<br>N -1.104101393 -2.011146206 -0.394755637<br>N -3.707604831 -0.903838563 -0.678832356<br>C -5.407550985 0.225655463 0.678035808<br>C -4.585324709 1.348019362 0.961663974<br>C -4.873152049 2.498783621 1.72566608<br>C -3.842296439 3.415013811 1.527034138<br>C -2.896608033 2.83435275 0.643866058<br>C -1.686266215 3.430587529 0.149784199<br>C -0.627336973 2.733099227 -0.378703298<br>C -0.369103031 1.309060715 -0.552708882<br>C 0.916632258 1.140328465 -0.966146682<br>C 1.555642856 2.43779433 -1.092870604<br>C 2.847207854 2.7862285 -1.409233136<br>C 3.93996107 1.85669961 -1.284150031<br>C 5.33935953 2.119051094 -1.614954928<br>C 6.065912221 1.064373033 -1.141406639<br>C 5.124389386 0.151938676 -0.512096875<br>C 5.383710979 -0.965794353 0.269567808<br>C 4.334443141 -1.700965863 0.892528312<br>C 4.354248074 -2.717783765 1.873586402<br>C 3.027436757 -3.024354905 2.202247061<br>C 2.18865052 -2.226983505 1.390304463<br>C 0.753039178 -2.208374801 1.211016091<br>C 0.24208439 -2.158061919 -0.06043077<br>C 0.919103251 -2.433731575 -1.327033049<br>C -0.008690656 -2.509314747 -2.315446387<br>C -1.325798989 -2.25176037 -1.748384368<br>C -2.562025677 -2.283050364 -2.355439401<br>C -3.737550247 -1.841544417 -1.645471546<br>C -5.08401626 -2.365058743 -1.808225023<br>C -5.864331218 -1.739396732 -0.876044234<br>C -5.01519061 -0.784179125 -0.187548538<br>H -3.002548546 0.886818646 -0.308771687<br>H 0.733574434 4.343773019 -0.653637245<br>H 2.77337791 -0.602499124 0.078297867

H -1.854275739 -1.711415549 0.212599738<br>H -5.747049583 2.638599078 2.341988552<br>H -3.756765604 4.389142057 1.979459728<br>H -1.077171154 0.511458599 -0.391674616<br>H 1.394627725 0.212127101 -1.227737375<br>H 5.722136667 2.987134278 -2.128474996<br>H 7.135751099 0.937368164 -1.207871559<br>H 5.240601355 -3.170531635 2.290364974<br>H 2.691953792 -3.777067715 2.897772736<br>H 1.982165704 -2.59340343 -1.416098498<br>H 0.166904149 -2.715238311 -3.359062288<br>H -5.373372492 -3.1242566 -2.518455073<br>H -6.921341043 -1.885421287 -0.712658641<br>C -6.79671589 0.233636263 1.264429856<br>C -1.551332239 4.932475818 0.272414148<br>C 3.162187041 4.234821343 -1.697596944<br>C 6.793636012 -1.407539857 0.559360574<br>C -0.143114884 -2.257657741 2.418704903<br>C -2.724156789 -2.805705718 -3.73025692<br>H -6.754857493 0.444935934 2.337269577<br>H -7.416749984 1.008402365 0.801089386<br>H -7.288525964 -0.729789231 1.135218102<br>H -0.837794789 5.218458735 1.056123318<br>H -1.204514704 5.367155332 -0.672980377<br>H -2.508542511 5.400236646 0.500375839<br>H 3.421339771 4.788433751 -0.785163065<br>H 4.010222544 4.324780567 -2.379088276<br>H 2.313030634 4.735522836 -2.173321589<br>H 6.902250837 -2.483704749 0.395034396<br>H 7.517861568 -0.890504854 -0.069050902<br>H 7.049869523 -1.207926155 1.605930907<br>H -0.395247392 -1.249327191 2.77118363<br>H -1.07767069 -2.785563233 2.20509573<br>H 0.358183336 -2.767672468 3.245797958<br>C -2.080792203 -3.982861354 -4.150350037<br>H -1.471074159 -4.532341659 -3.440960006<br>C -3.562146077 -2.142397362 -4.643903473<br>H -4.065074591 -1.233741915 -4.330165802<br>C -3.734755874 -2.628797979 -5.938047765<br>H -4.379905402 -2.098859438 -6.629749165<br>C -2.247258822 -4.464082514 -5.448044176<br>H -1.74581092 -5.376403516 -5.750972138<br>C -3.073468984 -3.788560585 -6.347630023<br>H -3.206119386 -4.164903259 -7.355424516
**28M$_{1a}$** (1.0 nN, C_1) Ph-substituent on the 6-position

N -3.579713579 1.70407516 0.187507597<br>N 0.544102481 3.348077928 -0.592535101<br>N 3.97053998 0.661644044 -0.561320469<br>N 3.251380753 -1.572950306 0.489387832<br>N -1.002068777 -2.076099382 -0.274796005<br>N -3.85431314 -0.810600528 -0.642056904<br>C -5.694233048 0.429392831 0.436397004<br>C -4.882057376 1.577253705 0.664507047<br>C -5.205081804 2.810349377 1.268737836<br>C -4.10826107 3.657753698 1.129347411<br>C -3.082608495 2.95479881 0.442191104<br>C -1.787457156 3.470582824 0.070844124<br>C -0.68052231 2.736231297 -0.29099152<br>C -0.409387945 1.307159674 -0.398956415<br>C 0.907637082 1.124278474 -0.7008307<br>C 1.564392532 2.417113405 -0.81849923<br>C 2.860396217 2.793751535 -1.101673633<br>C 4.001337817 1.908978254 -1.048909386<br>C 5.385078128 2.273643612 -1.368173383<br>C 6.171307289 1.210561083 -1.02892277<br>C 5.293036688 0.185142886 -0.495437002<br>C 5.623612526 -1.013515051 0.123856134<br>C 4.596199819 -1.849886981 0.67388985<br>C 4.651718621 -2.963391286 1.54066084<br>C 3.336501681 -3.308105468 1.886051498<br>C 2.465139735 -2.432242901 1.200056754<br>C 1.011066886 -2.377076087 1.141658183<br>C 0.377515564 -2.234566033 -0.063903938<br>C 0.943308048 -2.414685663 -1.399722821<br>C -0.063616111 -2.451665609 -2.305528127<br>C -1.337461153 -2.253737484 -1.619438063<br>C -2.600181701 -2.302763469 -2.162057707<br>C -3.796324295 -1.869766762 -1.465157526<br>C -5.109579518 -2.483179775 -1.605641372<br>C -5.966148883 -1.768684018 -0.816583231<br>C -5.200061745 -0.680511876 -0.238807881<br>H -3.167594858 0.914176978 -0.316268697<br>H 0.710813581 4.333861823 -0.494635223<br>H 3.010937464 -0.676198667 0.0342634<br>H -1.691706089 -1.87530587 0.432667983<br>H -6.14307236 3.049190704 1.74440373<br>H -4.035524084 4.666640177 1.49972903<br>H -1.138341174 0.519575398 -0.276544554<br>H 1.396653261 0.184317975 -0.889178809<br>H 5.722087105 3.209062714 -1.785471394<br>H 7.244487283 1.140443712 -1.124108874<br>H 5.551895662 -3.452627528 1.87939114

H 3.029960175 -4.127825323 2.516367095
H 1.998469992 -2.5382459 -1.587718974
H 0.024010677 -2.587805613 -3.371378356
H -5.326252092 -3.348708513 -2.212693218
H -7.02328856 -1.94077234 -0.679489332
C -7.131875862 0.444383062 0.940238355
C -1.62084913 4.97624962 0.117157229
C 3.110761185 4.26635383 -1.34394354
C 7.055058573 -1.4697901 0.355980921
C 0.2356852 -2.493374321 2.427618163
C -2.790571782 -2.824848958 -3.538569107
H -7.175331954 0.967856945 1.899336892
H -7.888273138 0.920279633 0.285145038
H -7.467190678 -0.579385423 1.117354865
H -0.992518336 5.294196293 0.959350484
H -1.155011215 5.33987881 -0.806868446
H -2.580295576 5.483570625 0.201572927
H 3.171135573 4.830113343 -0.40262014
H 4.045773805 4.433090174 -1.877364473
H 2.311034211 4.705271843 -1.950638477
H 7.146188213 -2.527044806 0.086949662
H 7.852486775 -0.930290742 -0.180561555
H 7.285679461 -1.394532084 1.425533116
H 0.057670351 -1.508595766 2.878009521
H -0.733793595 -2.977383013 2.2728535
H 0.796312763 -3.081221704 3.159306722
C -2.158818835 -4.003833699 -3.970351682
H -1.531004262 -4.550178519 -3.274329611
C -3.647033405 -2.161459981 -4.434665545
H -4.14370024 -1.253083004 -4.110468113
C -3.847225287 -2.648738387 -5.724481025
H -4.505643534 -2.118418896 -6.403235191
C -2.35430031 -4.487469268 -5.263321094
H -1.861260548 -5.401289944 -5.575370143
C -3.197882318 -3.811173979 -6.145909757
H -3.353246663 -4.188964854 -7.14993782
